# Copper‐Mediated Conversion of Complex Ethers to Esters: Enabling Biopolymer Depolymerisation under Mild Conditions

**DOI:** 10.1002/chem.202000088

**Published:** 2020-09-03

**Authors:** Ganyuan Xiao, James R. D. Montgomery, Christopher S. Lancefield, Isabella Panovic, Nicholas J. Westwood

**Affiliations:** ^1^ School of Chemistry and Biomedical Sciences Research Complex University of St Andrews and EaStChem North Haugh St Andrews Fife KY16 9ST UK

**Keywords:** biomass, copper, lignin, mild depolymerization, TEMPO oxidation

## Abstract

Selective processing of the β‐*O*‐4 unit in lignin is essential for the efficient depolymerisation of this biopolymer and therefore its successful integration into a biorefinery set‐up. An approach is described in which this unit is modified to incorporate a carboxylic ester with the goal of enabling the use of mild depolymerisation conditions. Inspired by preliminary results using a Cu/TEMPO/O_2_ system, a protocol was developed that gave the desired β‐*O*‐4‐containing ester in high yield using certain dimeric model compounds. The optimised reaction conditions were then applied to an oligomeric lignin model system. Extensive 2D NMR analysis demonstrated that analogous chemistry could be achieved with the oligomeric substrate. Mild depolymerisation of the ester‐containing oligomer delivered the expected aryl acid monomer.

## Graphical abstract

**Cu‐mediated conversion of complex ethers to esters**: Enabling biopolymer depolymerisation under mild conditions. A Cu/TEMPO system was used to modify lignin's β‐O‐4 unit to give ester‐containing chains that can be easily depolymerized. This chemistry was successfully applied to relevant dimeric and oligomeric substrates.

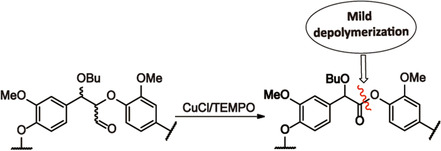

## Introduction

The efficient use of lignocellulosic biomass for the generation of renewable chemicals and biofuels requires the use of the biopolymer lignin. Whilst burning lignin remains one viable option, its high aromatic ring content provides an opportunity for renewable aromatic chemical feedstock production. However, depolymerising lignin to deliver the aromatic components remains challenging. A number of approaches exist including the “lignin‐first” approach.^1^ Alternatively, pretreatment of the lignocellulosic biomass can be used to generate a lignin‐rich product stream that can then be depolymerised in one,^2^ two^3^ or sometimes more^4^ steps. For example, we3b and others3e, 5 have reported α‐oxidation of the highly abundant β‐O‐4 followed by subsequent processing of the β‐O‐4^α‐OX^ units as a means of depolymerising lignin. Others3c, 3d, 6 have used an initial γ‐oxidation of the β‐O‐4 unit as the first step in lignin depolymerisation. Whilst in the majority of the current multistep approaches a relatively mild and controllable initial step (e.g. selective α‐oxidation) is used, it is also important that the last processing step avoids high temperatures and/or harsh chemical treatments. By combining mild conditions in both steps, it is possible to isolate the desired monomers and leave a residual lignin that can be used further.^7^

**Scheme 1:**
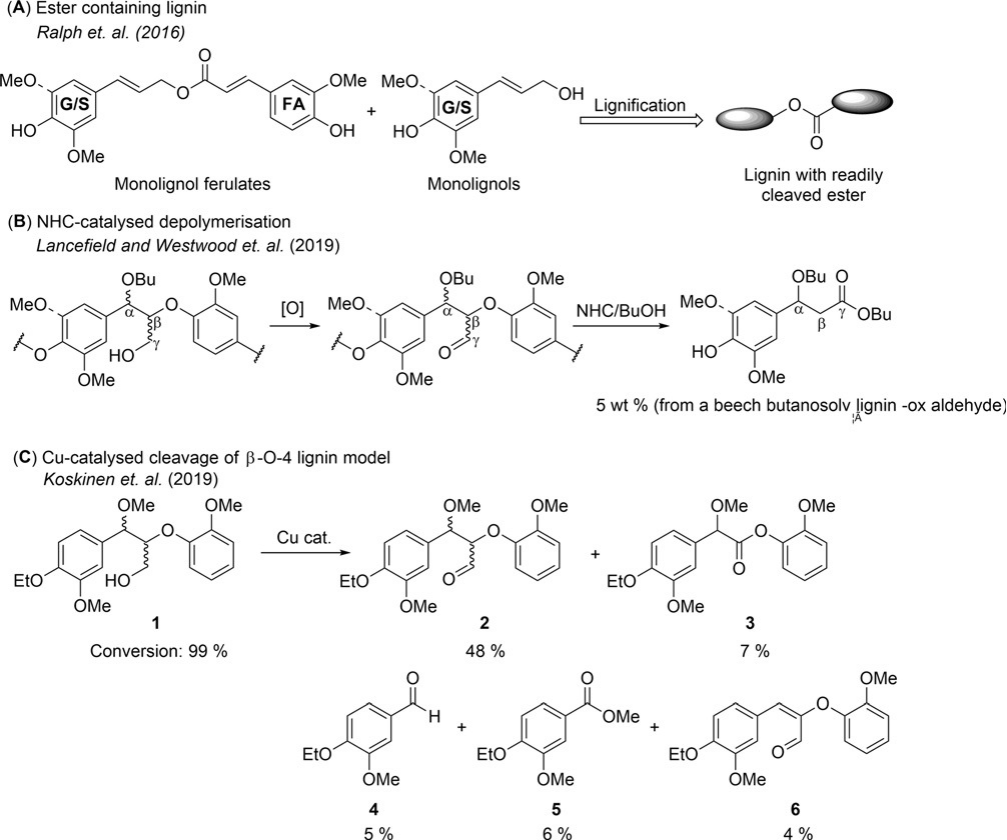
A) Incorporation of monolignol ferulates into lignin introduces chemically labile esters into the polymer backbone.8a, 8b B) 1° alcohol oxidation and subsequent NHC catalysis for the depolymerisation of butanosolv‐derived lignin β‐aryl ether linkages.3a C) Koskinen's experiment:^9^ 0.2 mmol scale in CH_3_CN (0.1 m); Cu cat.=10 mol % CuCl/10 mol % NaBF_4_/10 mol % BiPy (2,2’‐bipyridine)/10 mol % TEMPO (2,2,6,6‐tetramethylpiperidine 1‐oxyl)/10 mol % NMI (*N*‐methylimidazole) under an oxygen atmosphere.

In this context, we have recently become inspired by Bartley and Ralph's reports^8^ of “zip lignins” that contain ester groups (Scheme 1 A). Their work was based on the introduction of chemically labile ester bonds into the lignin polymer by encoding a feruloyl‐coenzyme A monolignol transferase into popular trees (*Populus alba* and *Populus grandidentata*). The presence of the ester group made the wood more prone to depolymerisation under relatively mild, alkaline conditions.^8^ An additional possible advantage of the presence of an ester in the lignin chain is that the use of mild cleavage conditions could result in the formation of smaller lignin chains which have not been condensed/ destroyed during a final depolymerisation step. We have therefore been searching for ways to modify selectively the β‐O‐4 unit to give lignin chains that contain ester functional groups.

Our initial efforts in this area focused on the use of NHC catalysed‐redox esterification but were ultimately derailed by the fact that the phenolic rebound reaction proved inefficient (Scheme S1). In the end we compromised and used butanol as an alternative rebound reactant leading to direct depolymerization and delivery of aromatic monomers from lignin (Scheme 1 B).3a However, during the course of our NHC studies we noticed a report by Koskinen et al.^9^ Their studies on the reaction of β‐O‐4 model compounds under CuCl/TEMPO/O_2_/NaBF_4_/BiPy/NMI/ CH_3_CN conditions included the reaction of the methylated β‐O‐4 model **1** ((α‐OMe)‐β‐O‐4) to give the corresponding γ‐aldehyde **2** as the major product along with a 7 % yield of the non‐cleaved aryl ester **3** (Scheme 1 C). The formation of small amounts of aldehyde **4**, methyl ester **5** and α, β‐unsaturated aldehyde **6** was also reported.

Despite the fact that production of **3** was not the focus of Koskinen's work, this observation was potentially relevant to our quest. In a separate part of our lignin programme we have focussed on a pretreatment protocol that uses butanol as a co‐solvent.^10^ Whilst others^11^ have used butanol in pretreatments, the existing studies did not discuss the almost full conversion of the β‐O‐4 unit to the corresponding (α‐OBu)‐β‐O‐4 unit that we observe under our conditions. This has led us to lignins that are rich in (α‐OBu)‐β‐O‐4 units analogous to the methylated unit ((α‐OMe)‐β‐O‐4) **1** used by Koskinen. We therefore decided to revisit Koskinen's work but using the (α‐OBu)‐β‐O‐4 unit rather than **1**.

## Results and Discussion

**Figure 1:**
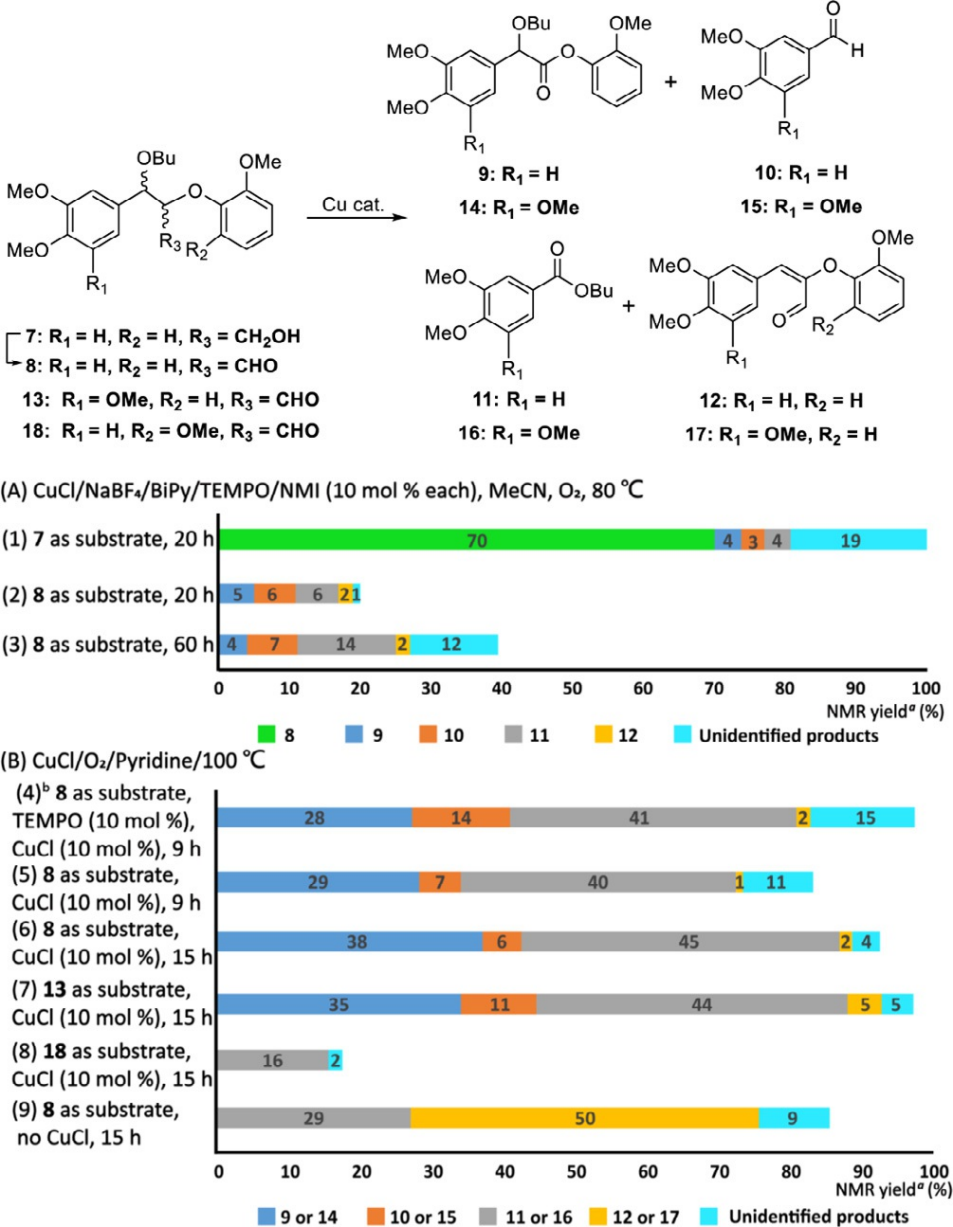
A) Results of Koskinen's conditions (CuCl/NaBF_4_/TEMPO/NMI (10 mol % each)/O_2_/MeCN) for the conversion of **7** and **8** (The method used to calculate conversions and yields is discussed in Figures S1–S3 and full spectra are shown in Figures S4–S6). B) Results of the original Baker's conditions (CuCl/TEMPO (10 mol % each)/O_2_/pyridine) for the conversion of **8** (entry 4) and modified Baker's conditions—CuCl (10 %)/O_2_/pyridine in the absence of TEMPO for the conversion of **8**, **13** and **18** (Figures S10 and S18–S22 for full spectra). [a] Reactions were performed on a 0.1 mmol scale. Conversions and yields were determined by ^1^H NMR using 1,3,5‐trimethoxybenzene as the internal standard. [b] Isolation of products from **8** (150 mg) using this condition gave **9** (31 %), **10** (12 %) and **11** (36 %).

We have previously reported the synthesis of a model of the (α‐OBu)‐β‐O‐4 unit **7** as a mixture of diastereomers.10a This study therefore started with the attempted conversion of **7** under Koskinen's conditions^9^ (20 hour reaction time, Figure 1 A, entry 1). As expected, the outcome was very similar to that of Koskinen's with the major product being the known3a, 12 γ‐aldehyde **8** (70 %, entry 1). Reassuringly, a small quantity of the corresponding ester **9** (4 %) was also formed. It was decided to repeat this reaction starting with **8** in an attempt to generate more of the desired **9**. After 20 hours (entry 2), overall conversion of **8** was low (20 %) and only small amounts of **9** (5 %) were formed. At a 60 hour extended time point (entry 3), whilst conversion of **8** increased to 39 %, the amount of **9** (4 %) remained very low with increased formation of ester **11** (14 %), resulting from cleavage of the C_α_−C_β_ bond in the β‐O‐4 unit, in addition to a range of unidentified products. Given the low yields of **9**, it was decided to abandon the Koskinen protocol.

Baker et al.^13^ have reported catalytic systems for the selective cleavage of lignin models using oxygen as the terminal oxidant. In parallel work, Baker's original protocol (CuCl/TEMPO/O_2_/Py) was being explored by us to achieve oxidative cleavage of **8** (Figures S7–S17 for a more detailed discussion). Efficient conversion (100 %) of **8** in the presence of sub‐stoichiometric amounts of CuCl and TEMPO in pyridine at 100 °C under an oxygen atmosphere (Figure 1 B, entry 4) resulted in the formation of butyl ester **11** as the major product (41 %; 36 % isolated yield). Importantly in the context of the current work, an increased yield of the desired ester **9** (28 %) was observed compared to the Koskinen protocol. Aldehyde **10** (14 %) along with trace amounts of known3a enal **12** (2 %) were also formed in this reaction.

The Baker system contains TEMPO, O_2_ and CuCl. Therefore it was decided to assess the role of each component in the reaction in the hope of biasing the system away from Cα‐Cβ bond cleavage in **8** and towards formation of ester **9**. Initial studies focussed on the use of CuCl under an oxygen atmosphere and in the absence of TEMPO. Results showed that TEMPO was not essential for the formation of **9** (c.f. Figure 1 B, entries 4 and 5) with high conversion (88 %) of **8** to **9** (29 %) and **11** (40 %) being achieved with no TEMPO in the reaction. Extending the reaction time to 15 hours at 100 °C (entry 6) led to a slight increase in the production of **9** (38 % at 95 % conversion of **8**) but more than half of the reaction products still resulted from Cα−Cβ bond cleavage. These conditions also efficiently converted the S‐G (α‐OBu)‐β‐O‐4 aldehyde **13** to the corresponding ester **14** (entry 7, 35 % at 100 % conversion of **13**) with **15**–**17** also being formed. In contrast, when these conditions were applied to the isomeric G‐S (α‐OBu)‐β‐O‐4 aldehyde **18** a much poorer outcome was obtained with an overall conversion of **18** of only 18 % and no observable formation of the analogous aryl ester (entry 8). Butyl ester **11** (16 %) was the only identifiable product from this reaction. To the best of our knowledge, this is the first time that a reactivity difference as a function of the methoxy‐substitution pattern in the β‐O‐4 unit has been observed under this type of reaction conditions.

**Scheme 2:**
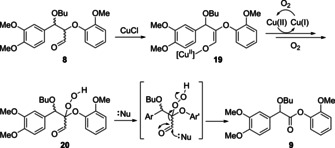
One possible mechanism for the formation of **9** under cat. CuCl/O_2_/pyridine conditions (based on literature findings^9^, 14b). Generic nucleophile Nu: could be water or pyridine.

In the absence of both TEMPO and CuCl (Figure 1 B, entry 9), efficient conversion of **8** (88 %) was still observed at 100 °C but none of the desired ester **9** was formed with the major product resulting from elimination of butanol to give **12**. This result confirmed that the presence of CuCl is essential for the formation of **9** under the Baker conditions. One possible mechanism (Scheme 2) involves the formation of the enolate^9^, ^14^ **19**, which could be converted to form the corresponding radical. The radical could be converted to the hydroperoxide intermediate **20**. Subsequent deformylation would be expected to give **9**. Possible reasons for the apparent lack of reactivity of the G‐S model **18** may be that the combination of the two radicals is more difficult due to increased steric constraints around a β‐carbon centred radical.

**Figure 2:**
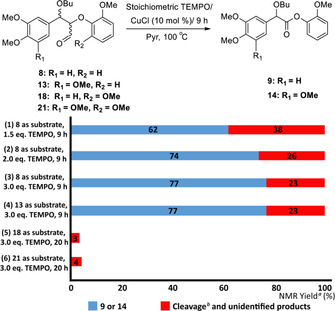
Results of catalytic CuCl/ stoichiometric TEMPO under Ar atmosphere for the conversion **8**, **13**, **18** and **21**. [a] Reactions were performed on a 0.1 mmol scale. Conversions and yields were determined by ^1^H NMR using 1,3,5‐trimethoxybenzene as the internal standard. [b] Additional data on the formation of the small amount of cleavage products are presented in Figures S24–S35.

Whilst the presence of CuCl is not essential for the formation of butyl ester **11** (Figure 1 B, entry 9), the yield of **11** is significantly decreased in its absence, indicating that **11** may be formed by two alternative pathways (Scheme S2). In several of the reactions (Figure 2 B, entries 5, 6 and 7), a small amount of aldehyde **10** (or **15** when **13** was used as the substrate) was formed in the presence of CuCl. Koskinen proposed that under their conditions, the formation of **10** from **1** is likely via 1,4‐addition of H_2_O to enal **12** followed by a rapid retro‐aldol reaction^9^ although other possible mechanisms could be considered (Scheme S3 and Figure S23).

Returning to the original Baker reaction conditions (Figure 1 B, entry 4), it was decided to rerun this reaction in the absence of oxygen to explore the contribution that TEMPO made to the production of **9** from **8** (Figure S**24**). As no oxygen was present and hence no chance of establishing a catalytic cycle involving oxygen, an excess of TEMPO was used. Under as carefully controlled conditions as could be achieved in the absence of a glove box (Figure S25 for a more detailed discussion), the model (α‐OBu)‐β‐O‐4 aldehyde **8** was efficiently converted to ester **9** using 1.5 equivalents of TEMPO, this time as the major product (62 %, Figure 2, entry 1). Increasing the number of equivalents of TEMPO led to a further increase in the yield of **9** (74 %, entry 2 and 77 %, entry 3) and these significant improvements in the formation of the desired ester were also observed for S‐G model **13** (entry 4). Very low conversions were again observed when the G‐S model **18** and the S‐S model **21** (entries 5 and 6) were used.

**Figure 3:**
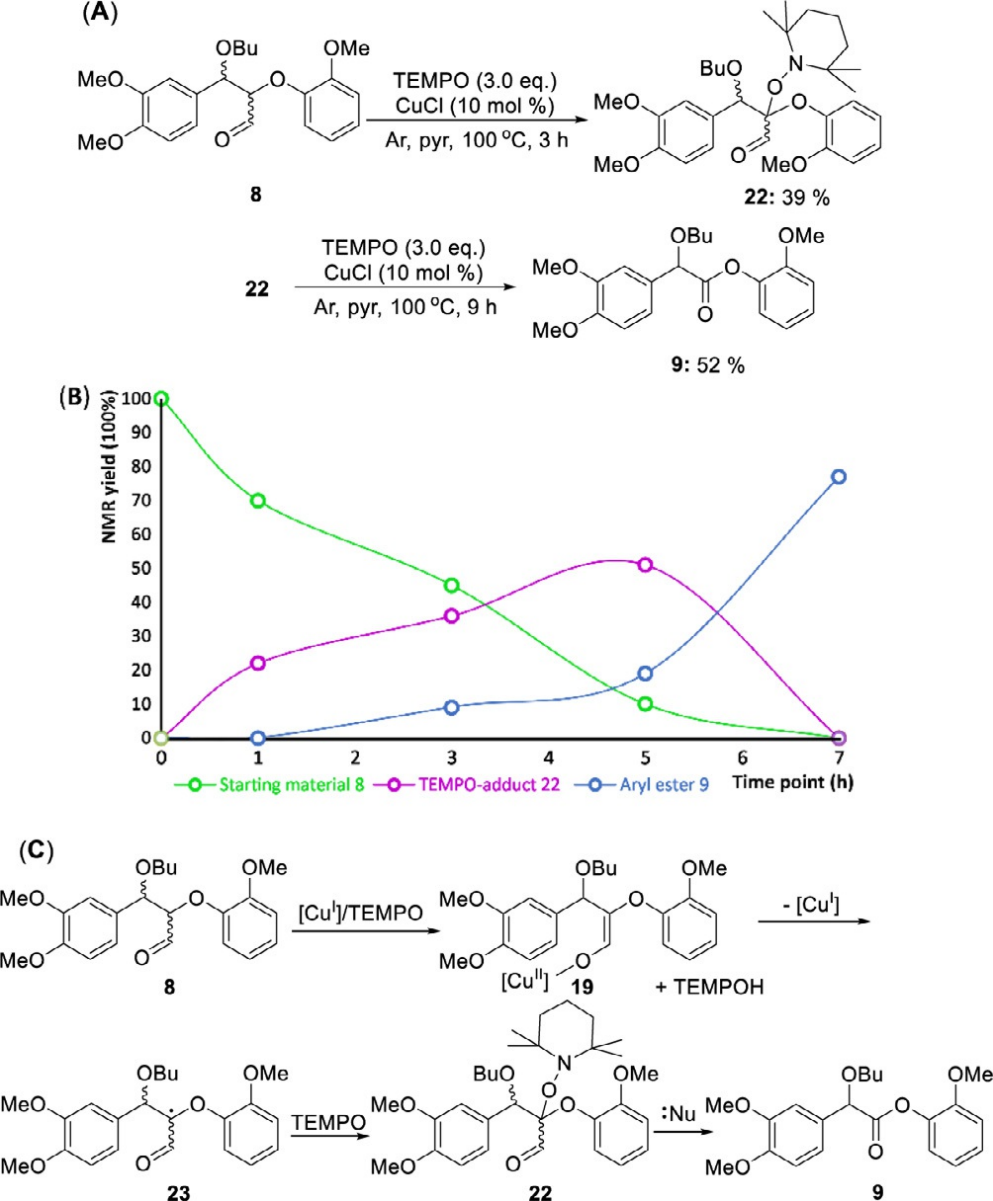
A) The synthesis of TEMPO‐adduct **22**; B) A graphical representation of the relationship between time vs. NMR yields (%) of **8**, **22** and **9** (Figures S36–S40 for a more detailed discussion); C) One possible mechanism for the CuCl/TEMPO‐catalysed oxidation of **8** to form **9**. Generic nucleophile Nu: used in deformylation.

Shortening the reaction time for the conversion of **8** under optimised conditions (CuCl (10 mol %)/TEMPO (3.0 equiv.)/Ar) to 3 hours led to the isolation of the TEMPO‐adduct **22** by column chromatography (39 % yield as a mixture of diastereomers, Figure 3 A). Related α‐ketone β‐TEMPO‐adducts have been reported previously.^9^, 13a, 15 A time course study supported a view that the starting material **8** was first converted to the TEMPO‐adduct **22** en route to ester **9** (Figure 3 B). When **22** was resubmitted to the reaction, ester **9** was obtained as expected (52 % yield, Figure 3 A).

The benzylic oxidation and subsequent retro‐aldol reaction often proposed for these types of reactions when native β‐*O*‐4 substrates are used^5^, ^9^, 13c is blocked in substrate **8** unless the α‐butoxylated lignin undergoes elimination of butanol and subsequent addition of extraneous water. Another possible mechanism could involve the formation of enolate^9^, ^14^ **19** (Figure 3 C) and formation of the corresponding radical **23**. The adduct **22** could then be formed by radical coupling of **23** with TEMPO. Subsequent deformylation, in an analogous manner to the reaction of **20** (Scheme 2), would convert **22** to **9**.

**Scheme 3:**
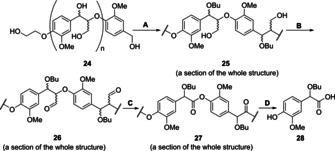
The oligomer synthesis: (A) 12 N. HCl: *n*BuOH (1: 33), 117 °C, 1.5 h, ≈100 %; (B) DMP (1.2 equiv.), DCM, rt., 4.0 h, 75 %; (C) TEMPO (3.0 equiv.), CuCl (10 mol %.), pyridine, 100 °C, 15 h, Ar., 100 %; (D) 0.5 m NaOH in MeOH, rt., 12 h, 7.0 wt % of **28**.

**Figure 4:**
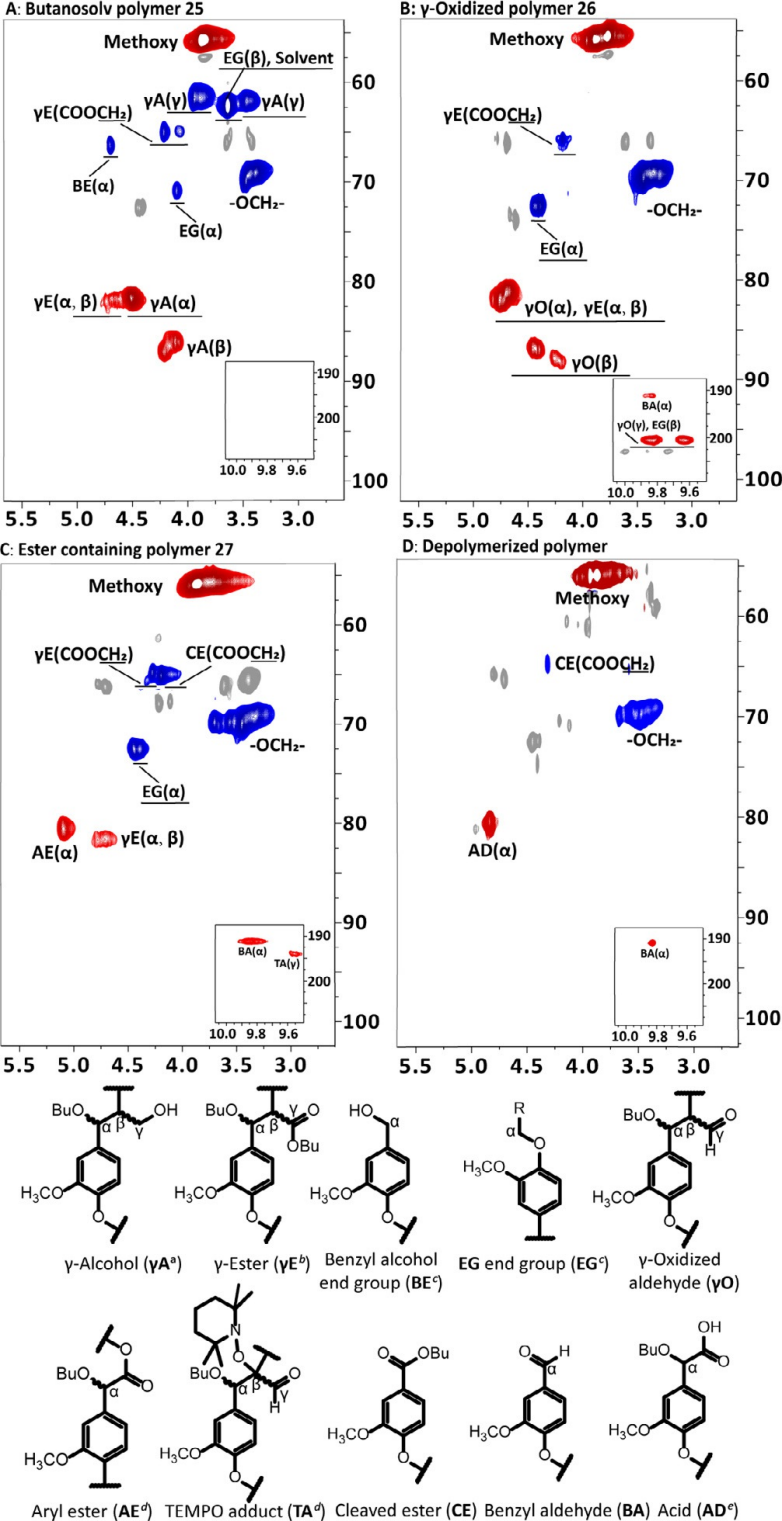
The oxygenated aliphatic region of the 2D HSQC NMR analysis of: A) Butanosolv β‐*O*‐4 oligomer **25**; B) butanosolv β‐O‐4 γ‐aldehyde oligomer **26**; C) Oligomer **27** formed on reaction of **26** with 3.0 equiv. TEMPO, 10 mol % CuCl, Pyridine, 100 °C, 15 h; D) Crude mixture of depolymerised products after hydrolysis of **27**. Full spectra are available in Figures S46–48 and S60. The chemical shifts of peaks in the aldehyde region have been corrected due to folding in the original analysis. [a] he assignment of signals in **γA** was determined by comparison the 2D HSQC spectra of **25** with that of the corresponding dimer model compound (Figures S45–S46). [b] The presence of signals corresponding to the γ‐ester unit (**γE**) was achieved by comparison with the 2D HSQC spectra of the corresponding dimer model compound (Figures S44–S46). [c] The signals corresponding to the α‐protons in the benzyl alcohol (**BE**) end group and the α and β‐protons of the ethylene glycol (**EG**) end group were assigned based on the literature.^18^ After the butanosolv step (Scheme 3, step A), R (in the ethylene glycol end group)=CH_2_OH; after oxidation step (steps B and C), R (in the ethylene glycol end group)=CHO; [d] In the dimer study, the HSQC NMR spectra showed that the signal corresponding to the α‐proton of ester **9** and the α‐proton of TEMPO‐adduct **22** were overlapping (Figure S50, entry E), making it hard to distinguish **AE** from **TA** using this signal. However, the presence of a **TA** unit was clear due to the presence of the aldehyde proton. In addition, HMBC analysis confirmed the formation of either **AE** or **TA** or both (Figures S51). [e] The formation of the acid unit **AD** is discussed in Figures S61.

Having identified optimised conditions for the formation of the desired esters **9** and **14** from the dimeric model compounds **8** and **13** respectively, the next challenge came in applying this chemistry to a model β‐O‐4‐containing oligomer. This step is required to assess whether it is possible using this methodology to prepare ester‐containing chains with minimal initial cleavage of the β‐O‐4 unit. Owing to the complexity of real samples of even butanosolv lignin, it was decided to use an artificial all β‐O‐4‐containing oligomer. As the TEMPO‐mediated ester formation reaction did not work on G‐S model **18** and S‐S model **21** (Figure 2, entries 5 and 6), studies focused on the all G β‐O‐4 oligomer (Scheme 3) which is a simplified model of a softwood lignin such as that obtained from Douglas fir wood.10b Oligomer **24** (M_w_ 2880, DP_n_ (degree of polymerisation) 8.13, Figures S41–S44 for a more detailed discussion) was synthesised using the previously reported route.3b, 17 In an extension to previous literature **24**, which contains native β‐O‐4 units, was converted to the corresponding α‐functionalised butanosolv β‐O‐4 oligomer **25** (Scheme 3 and Figure 4 A) using a modified butanosolv pretreatment10a, 12 in quantitative yield (Figures S45–S46). The subsequent γ‐oxidation reaction was then carried out using Dess–Martin periodinane (DMP)3a, 12 to give the required oligomeric substrate **26** (Figures 4 B and S47).

In brief, the key signals observed in the HSQC NMR analysis of oligomer **25** included those corresponding to the α (^1^H/^13^C *δ* 4.37–4.54 ppm/80.2–83.1 ppm), β (^1^H/^13^C *δ* 3.99–4.22 ppm/85.1–88.2 ppm) and the diastereotopic γ‐positions of the two diastereomers (^1^H/^13^C *δ* 3.32–3.46 ppm/61.3–63.1 ppm and ^1^H/^13^C *δ* 3.32–3.46 ppm/61.3–63.1 ppm and ^1^H/^13^C *δ* 3.72–3.92 ppm/60.7–62.6 ppm) of the **γA** unit. Signals corresponding to the two end groups (**BE** and **EG**) as well as small amounts of γ‐ester units (**γE**, Figures S45–S46 for a more detailed discussion), remaining from incomplete ester reduction during the synthesis of **25**, were also present. On oxidation of **25** to **26**, disappearance of the signals corresponding to the γ‐ alcohol positions of unit **γA** in **25** appeared linked to the presence of new signals corresponding to the γ‐aldehyde positions in the desired unit **γO** (^1^H/^13^C *δ* 9.57–9.68 ppm/199.6–201.5 ppm and ^1^H/^13^C *δ* 9.77–9.89 ppm/199.9–201.5 ppm, Figures 4 B and Figure S47).

Inspired by the dimer study (Figure 2, entry 2), an initial attempt to form the ester using oligomer **26** started by reacting **26** with TEMPO (2.0 equiv.) in the presence of CuCl (0.1 equiv.) in pyridine under an Ar atmosphere for 7 hours. The reaction outcome was encouraging but the major product contained predominantly TEMPO‐adduct **TA** units (Figure S52). Whilst studies on the simpler model **8** suggested that a reaction time of 7–9 hours may be ideal (Figure 3 B), it was clear that modified conditions would be required for the successful preparation of **27** containing predominantly ester units (Scheme 3). For example, when a reaction time of 15 hours was tried (using 3.0 equiv. TEMPO and 10 mol % CuCl) signals corresponding to the formation of the TEMPO‐adduct unit **TA** (minor component) and the desired ester unit **AE** (major component) were observed (Figure 4 C and Figure S48‐S51). Diagnostic changes in the HSQC analysis included the disappearance of the signals assigned to the β‐(^1^H/^13^C *δ* 4.34–4.51 ppm/85.7–88.1 ppm and ^1^H/^13^C *δ* 4.14–4.30/86.9–89.0 ppm) and γ‐positions of the two diastereomers (^1^H/^13^C *δ* 9.57–9.68 ppm/199.6–201.5 ppm and ^1^H/^13^C *δ* 9.77–9.89 ppm/199.9–201.5 ppm) in **γO** units in **26** and new signals corresponding to the α‐position of the aryl ester (**AE**) units (^1^H/^13^C *δ* 4.97–5.16 ppm/79.3–81.8 ppm) were observed (c.f. Figures 4 B and 4C).

**Figure 5:**
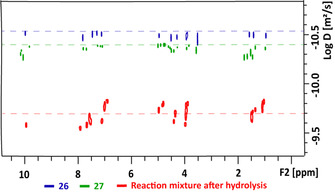
2D ^1^H DOSY spectrum of the **26**, **27** and the crude reaction mixture after hydrolysis of **27**.

Whilst the 2D HSQC analysis of **26** confirmed that the major reaction on forming **27** involved formation of the desired ester unit (**AE**), evidence for the formation of units formed by cleavage of the C_α_−C_β_ bond (units **CE** and **BA** in Figure 4 C) were also obtained (Figures S57 for a more detailed discussion), consistent with the dimer study result (Figures S**24**–**S25**). This was explored further using a previously reported^19^ 2D DOSY NMR approach this time on **26** and **27** to determine any apparent change in the molecular weight (MW) of the lignin models. There was, on average, a small increase in the diffusion coefficient on going from **26** (blue signals) to **27** (green signals, Figure 5), indicating a decrease in size during the reaction, which was consistent with the appearance of the signals observed for the **CE** unit in the HSQC analysis of **27** (Figure 4 C). On hydrolysis of **27** (Scheme 3, step D), a significant increase in the average diffusion coefficient of the crude product(s) was observed compared to both **26** and **27** (Figure 5). Subsequent purification of the crude products by column chromatography gave the expected acid **28** (Scheme 3, 7.0 wt % isolated yield). No attempts to optimise this yield have been made. In addition, a mixture of products (16.0 wt % isolated yield) that was shown to contain dimeric units was isolated from the column (Figures S59–S60 for a more detailed discussion). The isolation of these dimeric units (albeit as part of a mixture) was consistent with a relatively mild final step in the depolymerisation process allowing fragments of model oligomer chains to be obtained for potential future use (Figure S61 for a more detailed discussion).

## Conclusions

In summary, whilst preliminary studies based on the Koskinen protocol^21^ did not enable the efficient conversion of a modified β‐O‐4 unit to the desired ester‐containing unit, an effective method was developed based using the CuCl/TEMPO system in the absence of oxygen. This methodology was not only applicable to a dimeric β‐O‐4 model unit but also to an oligomeric β‐O‐4 model, leading to an oligomer chain (in **27**) that contained a significant number of internal ester units. A more detailed understanding of the key aspects of the CuCl/TEMPO/pyridine system was obtained. For example, the study confirmed that the desired ester was formed via TEMPO‐adduct **22** which was isolated and, on resubmission to the reaction conditions, was converted to the corresponding ester **9**. Detailed 2D NMR (HSQC, HMBC and DOSY) analysis showed that the reaction outcomes in both the dimeric and oligomeric models were analogous. It is envisaged that this method could be applied to a butanosolv lignin but challenges associated with the successful removal of oxygen and the amount of TEMPO are likely to limit the scale on which it can be applied.

## Conflict of interest

The authors declare no conflict of interest.

## Supporting information

As a service to our authors and readers, this journal provides supporting information supplied by the authors. Such materials are peer reviewed and may be re‐organized for online delivery, but are not copy‐edited or typeset. Technical support issues arising from supporting information (other than missing files) should be addressed to the authors.

Supplementary
