# Supplementary material for: Copper‐Mediated Conversion of Complex Ethers to Esters: Enabling Biopolymer Depolymerisation under Mild Conditions
Source: Chemistry. 2020 Sep 3;26(54):12397–402. doi: 10.1002/chem.202000088 (PMC7589252; doi:10.1002/chem.202000088)
Supplement: Supplementary file 1 — Supplementary [file CHEM-26-12397-s001.pdf]

Chemistry–A European Journal

Supporting Information

Copper-Mediated Conversion of Complex Ethers to Esters: Enabling Biopolymer Depolymerisation under Mild Conditions

Ganyuan Xiao, James R. D. Montgomery, Christopher S. Lancefield, Isabella Panovic, and Nicholas J. Westwood*[a]

Table of Contents

1. General Information **Pages S3**

2. Previously reported studies using NHC catalysis to form an ester group **Pages S4**

3. Use of the Koskinen Protocol **Pages S5-S9**

4. Summary of studies using Baker's catalytic CuCl/TEMPO/O_2/Pyridine conditions to achieve oxidative cleavage of the butanosolv γ-oxidised β-O-4 unit **Pages S10-S16**

5. General procedure for the Baker oxidation protocol **Pages S17-S18**

6. Oxidation of lignin model compounds 8, 13 and 18 using modified Baker's conditions: catalytic CuCl under an Oxygen atmosphere in the absence of TEMPO **Pages S19-S24**

7. Oxidation of lignin model compounds 8, 13, 18 and 21 by using catalytic CuCl/ stoichiometric TEMPO under Ar atmosphere **Pages S24-S35**

8. Hydrolysis of aryl ester **9** **Pages S36**

9. Synthesis of all-G β-O-4 γ-aldehyde model oligomers **25**-**27** **Pages S36-S42**

10. TEMPO oxidation of Y-CHO all-G β-O-4 γ-aldehyde model oligomer **27** **Pages S43-S50**

11. Hydrolysis of oxidized polymer **27** **Pages S50-S53**

12. NMR spectra of synthesized compounds **Pages S54-61**

13. References **Pages S62**

1. General Information

All commercially available compounds were purchased and used as received unless otherwise stated. Pyridine was refluxed with CaH_2 overnight, followed by distillation and degassed by freeze-pump-thaw cycling. For all air and moisture sensitive reactions, a flame dried Schlenk flask under an argon atmosphere was used and pyridine was freshly dried and degassed. ^{1}H NMR and ^{13}C NMR analysis was performed on a Bruker Avance II 400 MHz, a Bruker Avance III 500 MHz spectrometer equipped with a nitrogen cooled (Prodigy) BBO probe or a Bruker Avance III 700 MHz spectrometer equipped with a nitrogen cooled (Prodigy) TCI probe with the solvent peak used as the internal standard. Multiplicities are described using the following abbreviations: s = singlet, d = doublet, t = triplet, q = quartet and m = multiplet and the J couplings are reported in Hz. NMR spectra were processed using TopSpin 3.1 (PC version) or MestReNova. ^{1}H NMR data is provided for literature compounds. Full characterisation data consisting of ^{1}H NMR, ^{13}C NMR, IR, M.P. (if applicable) and HRMS are provided for all novel compounds. Column chromatography was performed using Davisil® silica (40-63 μm, 230-400 mesh). Thin layer chromatography was performed on pre-coated glass plates (Silica Gel 60A, Fluorochem) and visualised under UV light (254 nm) or by staining with $KMnO_4$. IR spectra were obtained on a Shimadzu IRAffinity-1 Fourier transform IR spectrophotometer as thin films. Analysis was carried out using Shimadzu IR solution v1.50 and only characteristic peaks are recorded. Mass spectrometry data were acquired via the University of St Andrews School of Chemistry mass spectrometry service by Mrs Carolyn Horsburgh.

For polymer samples, DOSY experiments were performed using the *ledbpgp2s* pulse sequence. The diffusion delay (Δ) and gradient pulse length (δ) were optimized for each sample in order to achieve *ca.* 5-10% residual signal at 98% gradient strength (compared to 10% gradient strength) using the 1D DOSY experiment with the *ledbpgp2s1d* pulse sequence. Each *pseudo*-2D experiment consisted of a series of 32 spectra acquired with 65536 data points. The gradient pulses were incremented from 10% to 98% with a linear ramp. The temperature was set and maintained at 295 K. Data sets were processed by Fourier transformation in *F2,* using line broadening of 10 Hz, followed by a baseline correction. The DOSY analysis was then performed in Bruker Dynamics Center 2.3. Manual peak picking was performed for each dataset and peak intensities were used to measure the signal decay. Error estimation of the fit was performed at the 95% confidence level. All samples were prepared by dissolving 60 mg of material in 0.7 mL of *d6*-DMSO. All samples were allowed to thermally equilibrate prior to optimizing DOSY parameters (Δ and δ).

2. Previously reported studies using NHC catalysis to form an ester group[S1]

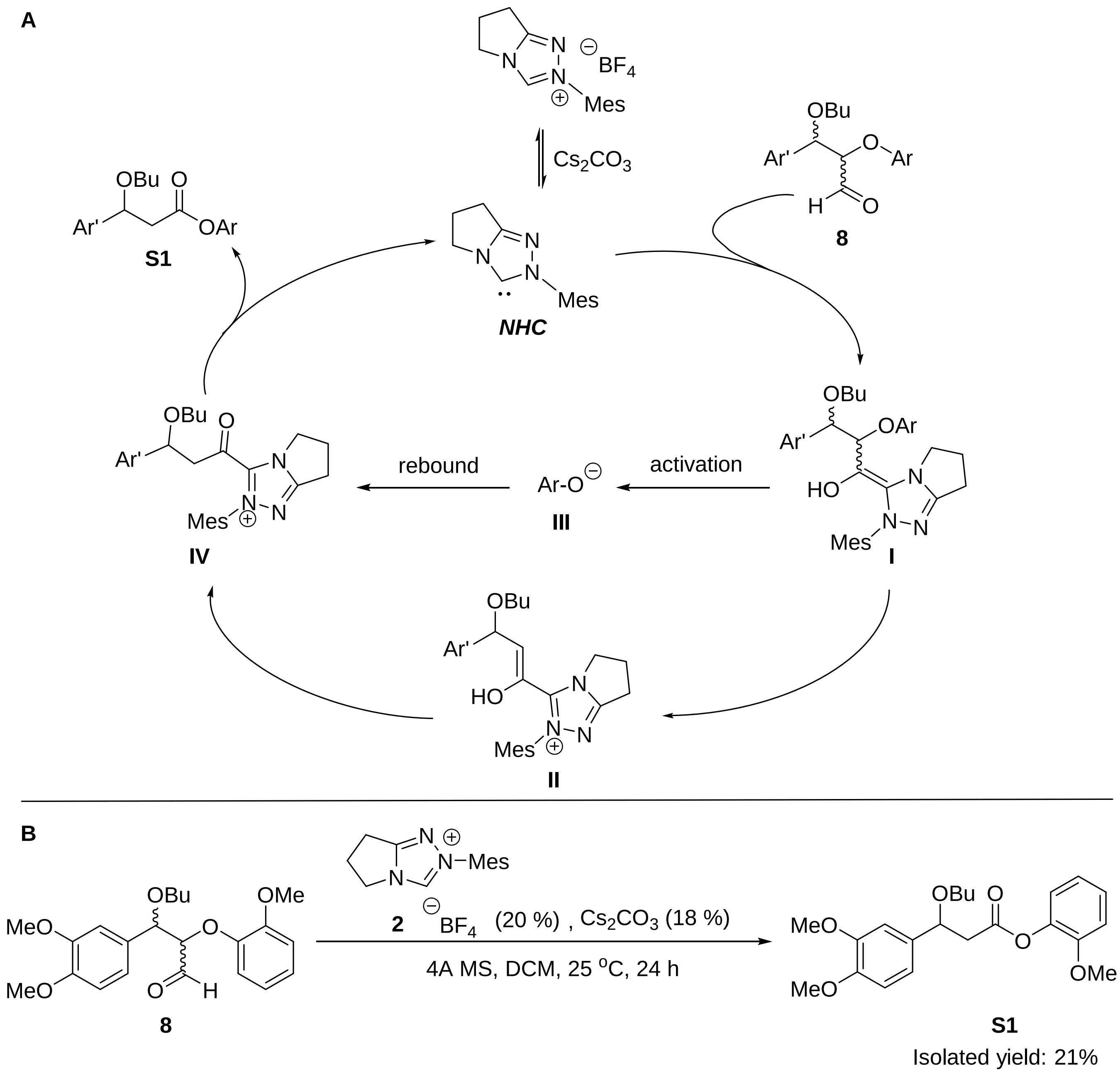

Scheme S1. Previous attempts to convert the β-O-4 unit into an ester containing unit[S1]: (**A**) The proposed mechanism of the NHC-catalysed redox esterification using α-phenoxyaldehyde **8** as the starting material (see **Section B** of this scheme for full structure of **8**). Initially the Breslow intermediate **I** is formed, after which the aryloxide **III** functions as a leaving group leading to the formation of **II. II** can tautomerize to form acyl azolium **IV,** onto which the aryloxide **III** can rebound to form the desired aryl ester **S1**. (**B**) Previously reported study [S1] using model β-O-4 dimer **8** showed that the rebound reaction was inefficient in this system, giving a low yield of **S1** (21%).

3. Use of the Koskinen Protocol[S2]

3.1. General procedure

The procedure was based on literature precedent.[S2] To a flame-dried round bottom flask (5 mL) was added CuCl (1.0 mg, 0.01 mmol, 10 mol %[a]), $NaBF_4$ (1.1 mg, 0.01 mmol 10 mol %), 0.5 mL of a stock solution of model compound **7** or **8** (0.2 M, 0.1 mmol, 100 mol %)[b] in CH_3CN, 0.1 mL of stock solution of 2,2'-bipyridine (0.1 M, 0.01 mmol, 10 mol %) in CH_3CN, 0.1 mL of a freshly prepared stock solution of TEMPO (0.1 M, 0.01 mmol, 10 mol %) in CH_3CN and 0.1 mL of a stock solution of NMI (0.1 M, 0.01 mmol, 10 mol %) in CH_3CN, followed by the addition of CH_3CN (0.2 mL), giving a final concentration of substrate of 0.1 M.[c] Oxygen was bubbled through the mixture for 1 minute and the flask was then sealed with a septum and an O_2 balloon was used. The reaction was then stirred at 80 °C for the assigned number of hours. After cooling to rt, 1 mL of a standard solution of 1,3,5-trimethoxybenzene in CH_3CN (10.8 mg/mL, 0.0599 M) was added and the resulting mixture was passed through a short silica column followed by washing of the column with DCM/ acetone (1:1, 10 mL). The filtrate was concentrated *in vacuo* and the crude reaction mixture was subjected to quantitative ^1H NMR analysis. Full spectra are shown in **Figures S4-S6.**

[a] Note that the actual concentration of starting material **8** was lower than determined by weighing (see quantitative NMR analysis below) and so slightly larger mol % of the CuCl, $NaBF_4$, 2,2'-bipyridine and NMI were used in these reactions compared to the original Koskinen protocol.

[b] Concentration of the stock solution of the starting material was determined by quantitative ^1H NMR using an internal standard 1,3,5-trimethoxybenzene in CH_3CN (10.8 mg/mL, 0.0599 M).

[c]All substrates, reagents and internal standard solutions were added using an Eppendorf pipette.

Throughout detailed NMR analysis of quantitative ^1H NMR spectra has been used to calculate NMR yields. **Figure S1** below provides a comparison of the analysis of one selected reaction with authentic samples of the main products observed to illustrate how peaks that correspond to the different products were selected.

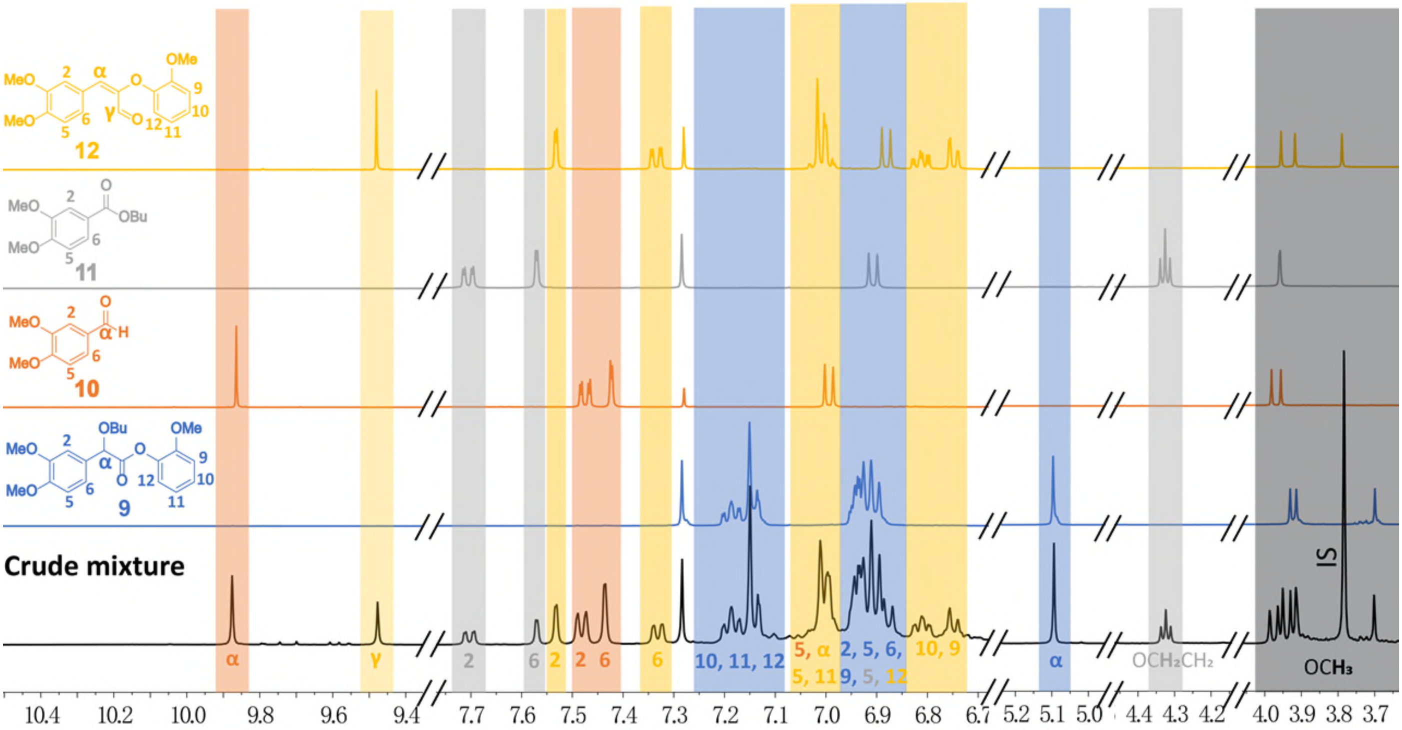

Figure S1: Comparison of the ^1H NMR spectra of products from a CuCl/TEMPO reaction on dimeric model **8**. Peaks that are diagnostic for the different products are highlighted.

3.2. An example of the calculation of reaction conversion and product NMR yields by using the Koskinen protocol[S2]

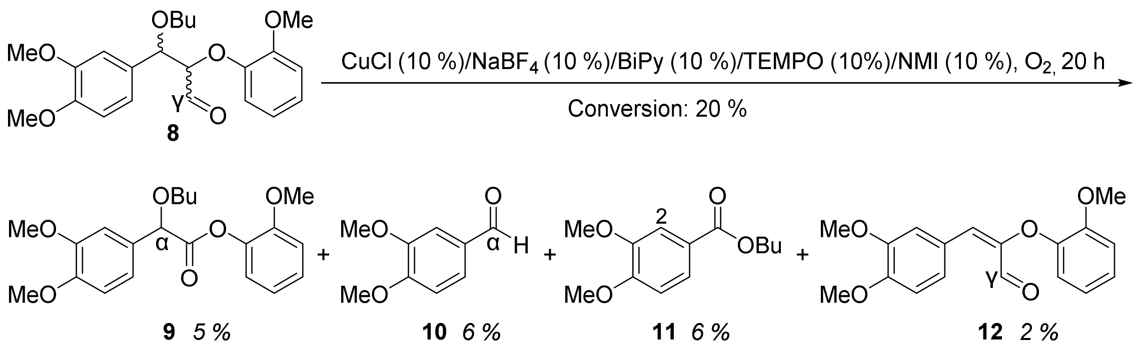

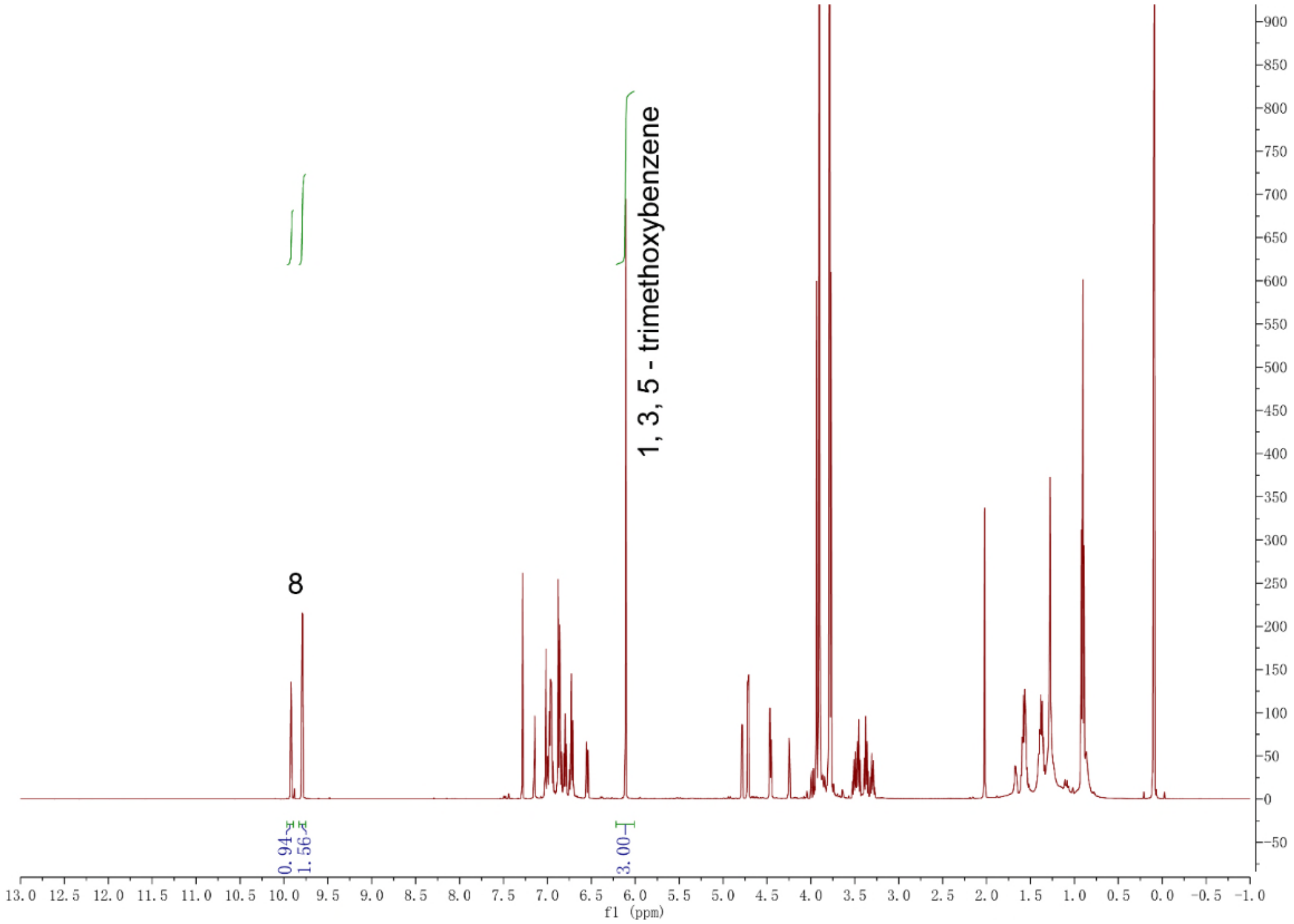

Figure S2: The quantitative ^{1}H NMR spectrum of starting material **8** with internal standard. Adding 0.3 mL of a standard solution of **8** in CH_3CN and 0.3 mL of internal standard 1,3,5-trimethoxybenzene solution (10.8 mg/mL, 0.0599 mol/L) in CH_3CN by Eppendorf pipette, the solvent was evaporated and the sample was then dissolved in 0.5 mL *d*-$CDCl_3$ and subjected to quantitative ^{1}H NMR analysis. The integral value of the observed signal for the internal standard was set to 3.

The first step in calculating the conversion of the starting material and the NMR yields of the products was to determine the actual concentration of the starting material (in this case **8**) in the reaction mixture. This was done using the following equation and the quantitative ^{1}H NMR spectrum shown in **Figure S2**:

Concentration of **8** = c(IS)×V(IS)×(I(SM)/(I(IS)/3)/V(SM) = 0.0599 M × 0.3 mL × ((0.94 + 1.56)/(3/3)) / 0.3 mL = 0.15 M

c(IS) = concentration of the internal standard solution (M)

V(IS) = volume of the internal standard solution added (mL)
I(SM) = sum of the integrals of the γ-proton for each diastereomer of the starting material **8**
I(IS) = integral of the 3 magnetically identical protons in the internal standard
V(SM) = volume of the standard solution of the starting material added (mL)

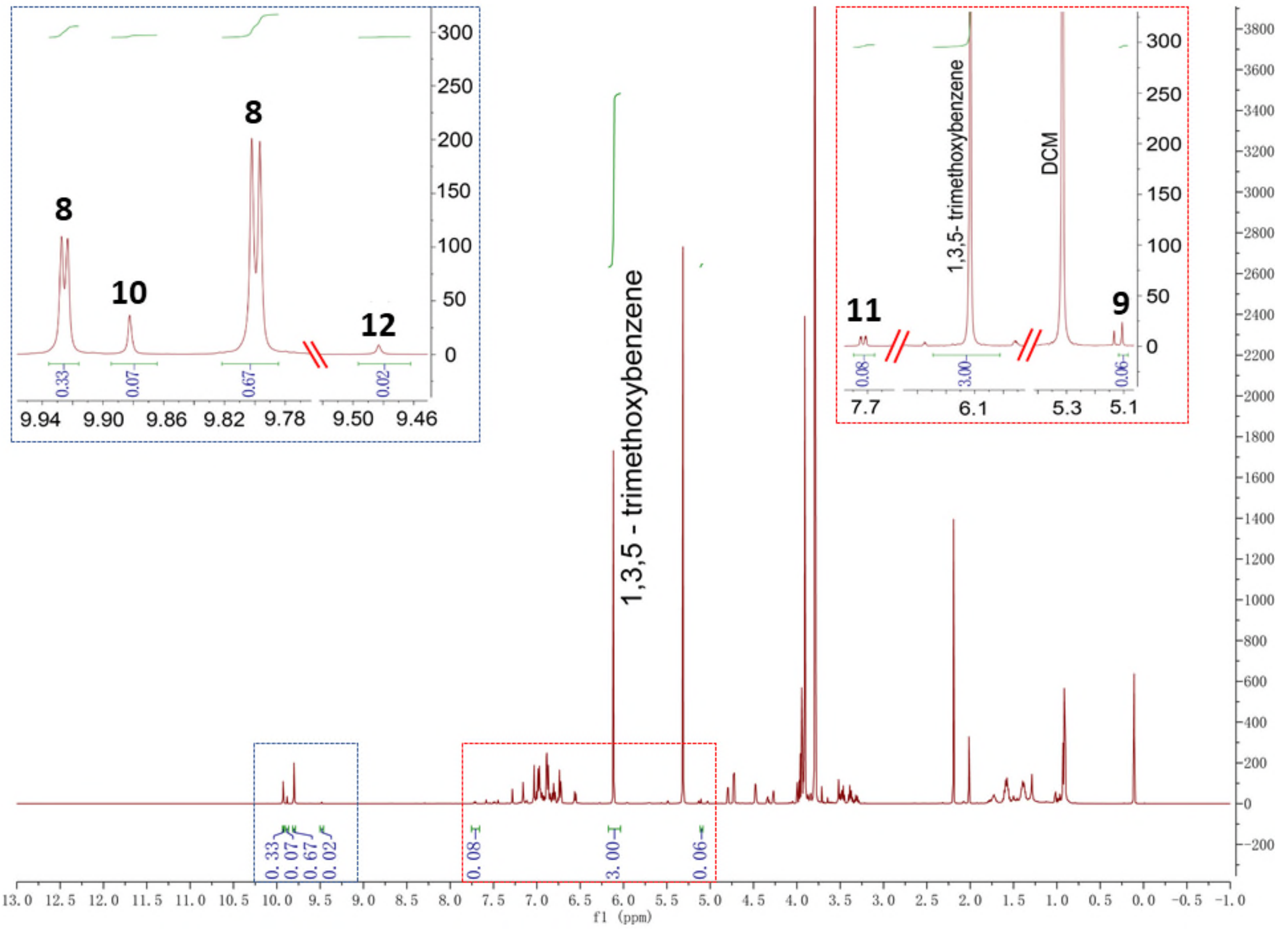

Figure S3: The quantitative ^{1}H NMR spectrum after the Koskinen oxidation protocol. 0.5 mL of a standard solution of starting material **8** was added to the reaction. After cooling to rt, 1 mL of standard solution of 1,3,5-trimethoxybenzene (10.8 mg/mL, 0.0599 M) was added.

In the second step, the following equation was used to calculate the overall conversion of the starting material in conjunction with the quantitative ^{1}H NMR spectrum in **Figure S3**.

Conversion of reaction = [1 - c(IS)×V(IS)× (I(SM)/(I(IS)/3)/c(SM)×V(SM)] × 100 % = [1 - 0.0599 M × 1.0 mL × ((0.33 + 0.67)/(3/3)) / 0.15 M × 0.5 mL] × 100 % = 20 %

NMR yield of **9**: c(IS)×V(IS)× (I(**9**)/(I(IS)/3)/c(SM)×V(SM) × 100 % = (0.0599 M × 1.0 mL × 0.06)/ (0.15 M × 0.5 mL) × 100 % = 5 %

NMR yield of **10**: c(IS)×V(IS)× (I(**10**)/(I(IS)/3)/c(SM)×V(SM) × 100 % = (0.0599 M × 1.0 mL × 0.07)/ (0.15 M × 0.5 mL) × 100 % = 6 %

NMR yield of **11**: c(IS)×V(IS)× (I(**11**)/(I(IS)/3)/c(SM)×V(SM) × 100 % = (0.0599 M × 1.0 mL × 0.08)/ (0.15 M × 0.5 mL) × 100 % = 6 %

NMR yield of **12:** c(IS)×V(IS)× (I(12)/(I(IS)/3)/c(SM)×V(SM) × 100 % = (0.0599 M × 1.0 mL × 0.02)/ (0.15 M × 0.5 mL) × 100 % = 2 %

c(IS): concentration of internal standard (M).
V(IS): volume of internal standard (mL).
I(SM) = sum of the integrals of the γ-proton for each diastereomer of the starting material **8**.
I(IS) = integral of the 3 magnetically identical protons in the internal standard
I(**9**): integral of α proton of **9**.
I(**10**): integral of α proton of **10**.
I(**11**): integral of H2 of **11**.
I(**12**): integral of γ proton of starting material **12**.
c(SM): calibrated concentration of starting material **8** (M).
V(SM): volume of starting material (mL)

The spectra used to generate the bar chart **Figure 1A entries 1-3** in the manuscript are shown in **Figures S4-S6**.

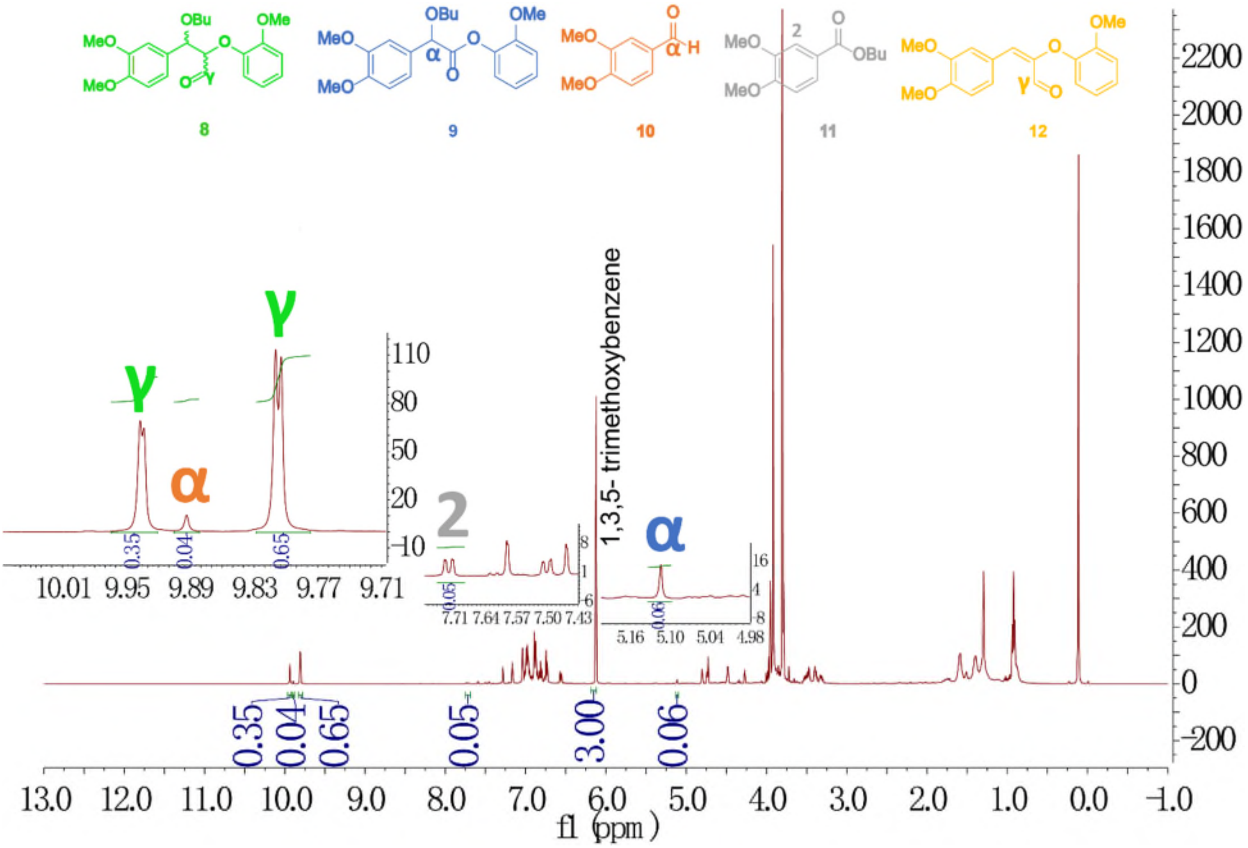

Figure S4: Quantitative ^{1}H NMR spectrum ($CDCl_3$) for the oxidation of **7** using the Koskinen protocol[S2]: CuCl (10 mol %), $NaBF_4$ (10 mol %), BiPy (10 mol %), TEMPO (10 mol %), NMI (10 mol %), O_2 in acetonitrile for 20 hours. (**Figure 1A**, **entry 1** in manuscript)

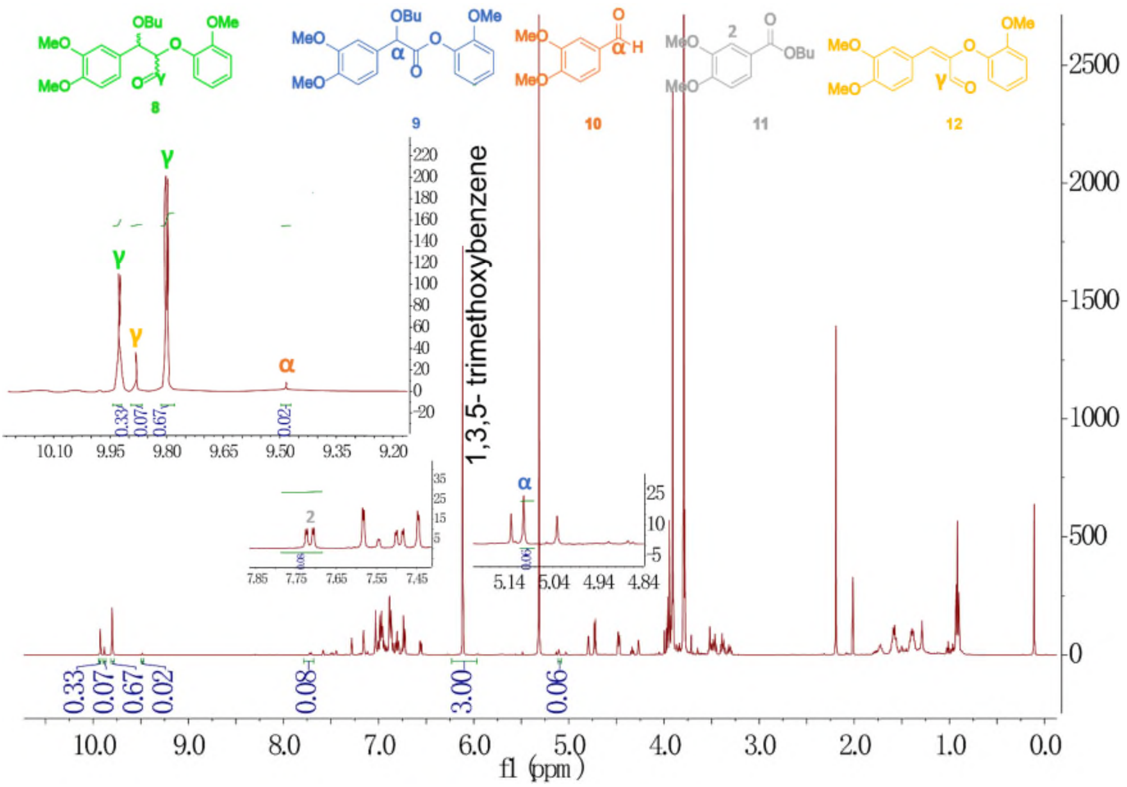

Figure S5: Quantitative ^{1}H NMR spectrum ($CDCl_3$) for the oxidation of **8** using the Koskinen protocol[S2]: CuCl (10 mol %), $NaBF_4$ (10 mol %), BiPy (10 mol %), TEMPO (10 mol %), NMI (10 mol %), O_2 in acetonitrile for 20 hours. (**Figure 1A**, **entry 2** in manuscript)

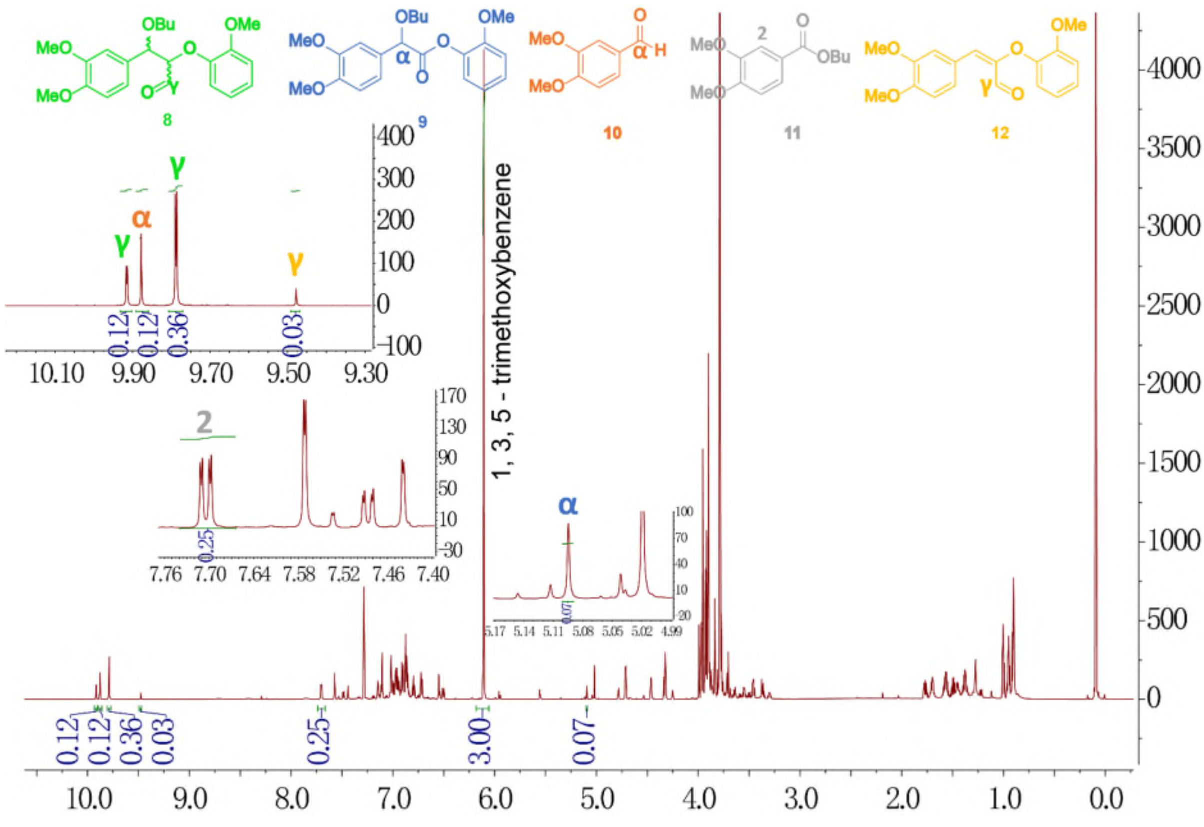

Figure S6: Quantitative ^{1}H NMR spectrum ($CDCl_3$) for the oxidation of **8** using the Koskinen protocol[S2]: CuCl (10 mol %), $NaBF_4$ (10 mol %), BiPy (10 mol %), TEMPO (10 mol %), NMI (10 mol %), O_2 in acetonitrile for 60 hours. (**Figure 1A**, **entry 3** in manuscript)

4. Summary of studies using Baker's catalytic CuCl/TEMPO/O_2/Pyridine conditions[S3] to achieve oxidative cleavage of the butanosolv γ-oxidised β-O-4 unit

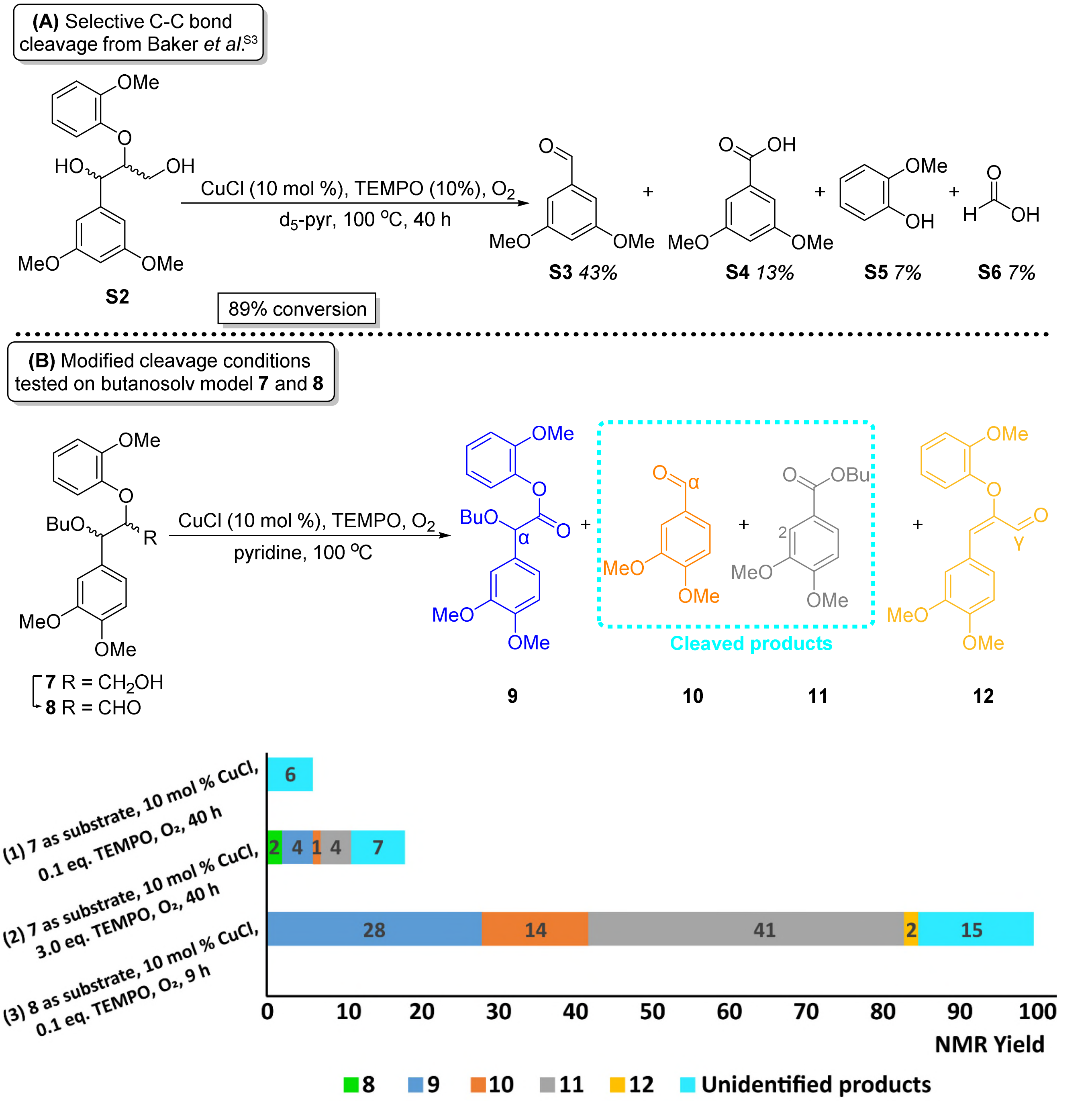

Figure S7: (**A**) Baker *et al.*'s oxidative cleavage system[S3]. Using model **S2**, Baker observed C_α-C_β bond cleavage giving monoaromatic products **S3**, **S4**, guaiacol (**S5**) and formic acid (**S6**), with a conversion of **S2** of 89% after 40 h. (**B**) Baker's catalytic system was used by us for the cleavage of models **7** and **8.** Application of this protocol to unoxidized butanosolv model **7** led to quite low conversion (6 % conversion, **entry 1**). Reaction of **7** with 3.0 eq. TEMPO in pyridine for 40 h under an oxygen atmosphere resulted in only 18% conversion (**entry 2**), as γ-oxidation of **7** occurred to a very limited degree in the presence of TEMPO/CuCl/O_2. When the Baker conditions were tested with **8** (**entry 3**), 100 % conversion of **8** was observed within 9 hrs with 55 % of the products being monoaromatic cleavage products **10** and **11**. Importantly, a 28 % NMR yield of non-cleaved aryl ester **9** was observed, which raised the possibility of forming ester-containing β-O-4 units in lignin using this system. Reactions were conducted on a 0.1 mmol scale following the general procedure **5**. Yields were determined by quantitative ^{1}H NMR analysis using 1,3,5 – trimethoxybenzene as the internal standard. The spectra used to generate the bar chart **Figure S7B**, **entry 1-3** are shown in **Figures S8-S10**.

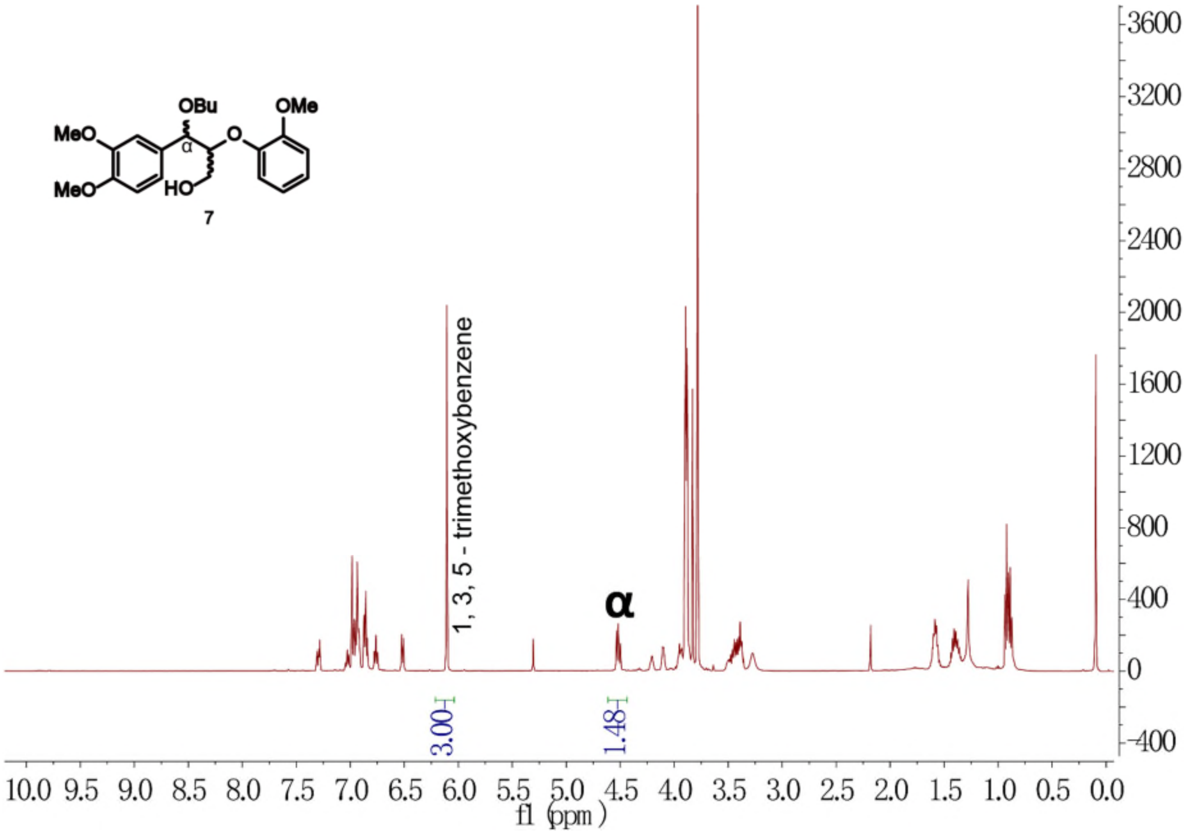

Figure S8: Quantitative ^{1}H NMR spectrum ($CDCl_3$) for the oxidation of **7** with Baker's originally reported conditions[S3]: CuCl (10 mol %), TEMPO (0.1 eq.), O_2 in pyridine for 40 hours. (**Figure S7B**, **entry 1**)

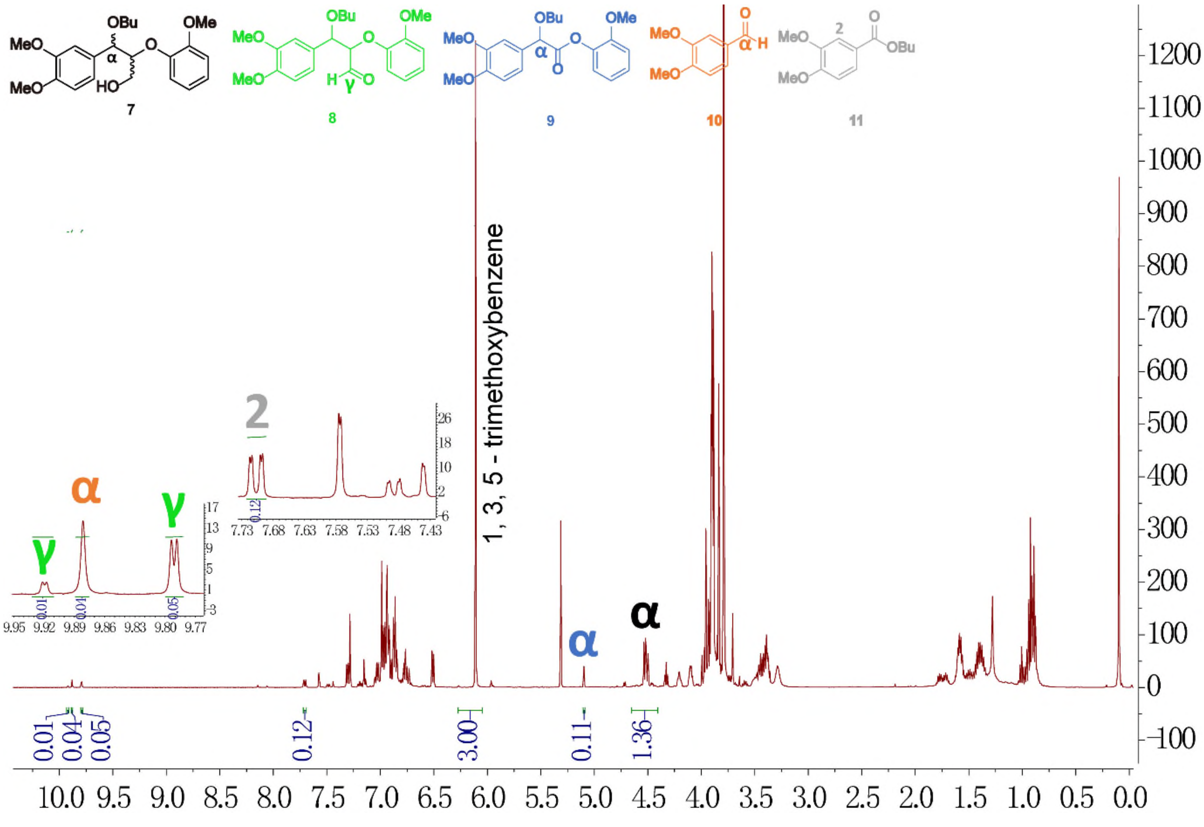

Figure S9: Quantitative ^{1}H NMR spectrum ($CDCl_3$) for the oxidation of **7** with modified Baker's conditions: CuCl (10 mol %), TEMPO (3.0 eq.), O_2 in pyridine for 40 hours. (**Figure S7B, entry 2**)

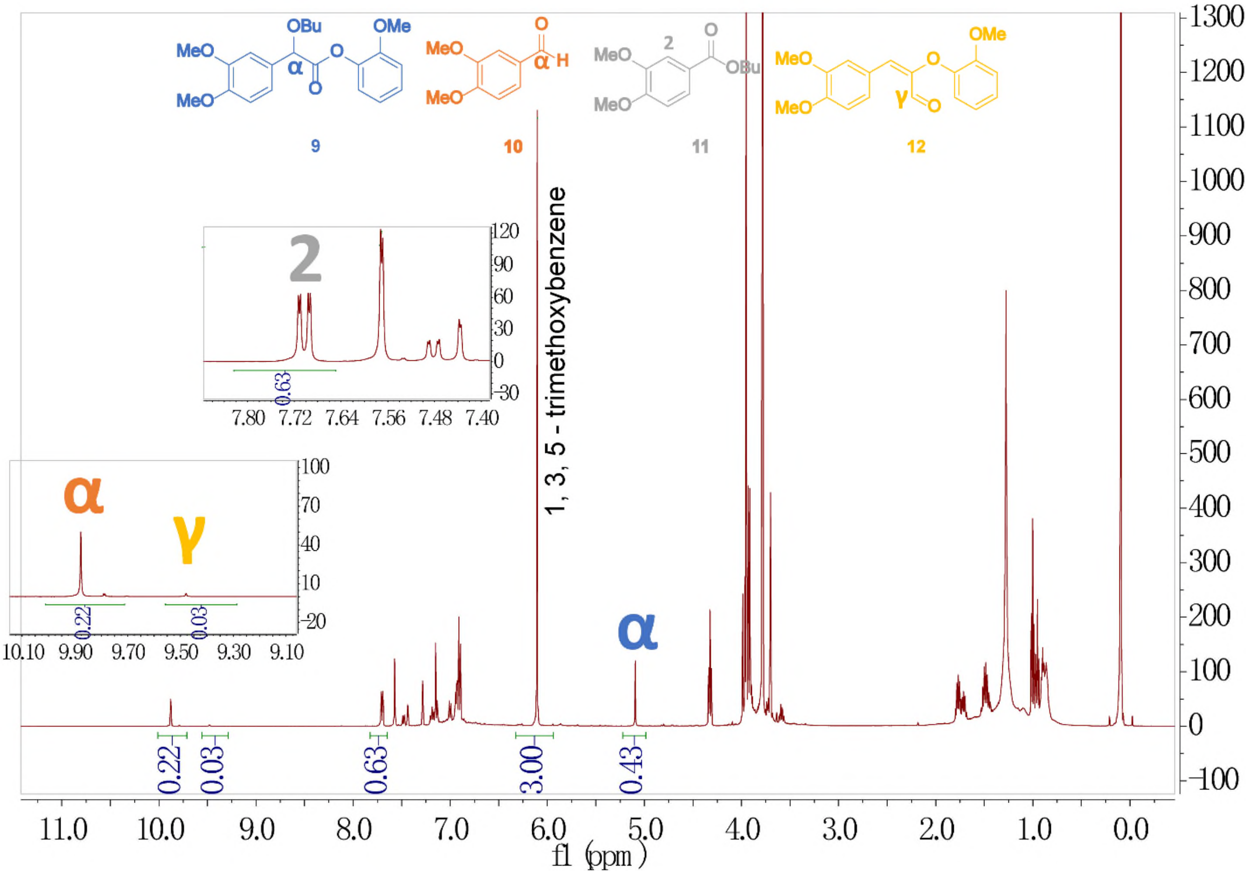

Figure S10: Quantitative ^{1}H NMR spectrum ($CDCl_3$) for the oxidation of **8** with modified Baler's conditions: TEMPO (10 mol%), CuCl (10 mol %), O_2 in pyridine for 9 hours. (**Figure 1B, entry 4** in manuscript; **Figure S7**, **entry 3** and **Figure S11, entry 1**)

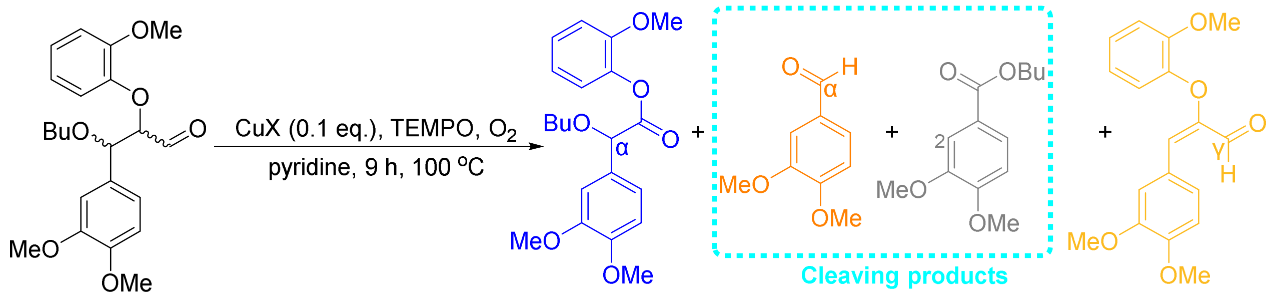

8 **9** **10** **11** **12**

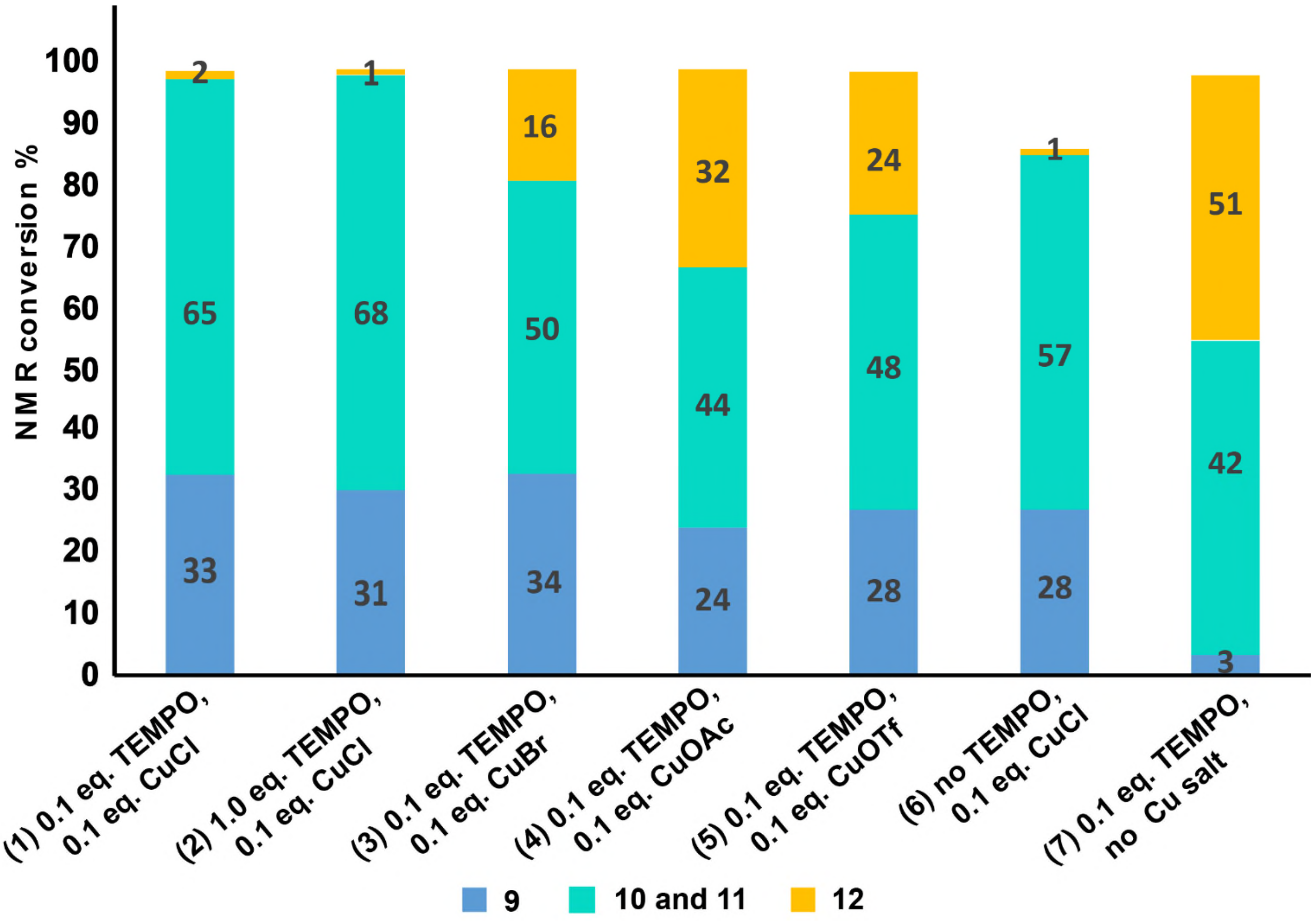

Figure S11: Attempted optimization of Baker's CuCl/TEMPO/O_2 system[S3] applied to dimeric model **8**. The initial goal in the parallel study was to find conditions to give increased amounts of cleavage products **10** and **11** and to minimize production of non-cleavage products **9** and **12**. Increasing the amount of TEMPO used in this reaction did not enhance the cleavage reaction further (*c.f.* entries **1** and **2**). A small screen of other copper catalysts was also conducted (entries **3**-**5**), although CuCl remained the most effective. CuBr, CuOAc and CuOTf all gave an increased yield of enal **12**. Starting material **8** was still consumed in the presence of only CuCl and O_2 (no TEMPO, entry **6**), or in the absence of CuCl (with TEMPO and O_2, entry **7**). Importantly in the context of this manuscript, aryl ester **9** was detected in significant amounts in all of these reactions except in the absence of a source of copper ions. Reactions were conducted on a 0.1 mmol scale following the general procedure **5**. The percentage conversion in these reactions was calculated from quantitative ^{1}H NMR analysis of the crude reaction mixture. The percentage conversion was based on a comparison of the integrals of the following peaks corresponding to: (i) the γ-proton of the starting material **8**; (ii) the α-proton of aryl ester **9**; (iii) the α-proton of aldehyde **10**; (iv) the H2 in butyl ester **11**; (v) the γ-proton of enal **12**. Full spectra are shown in **Figures S10** (for entry **1**) and **Figures S12-S17** (for entries **2**-**7**).

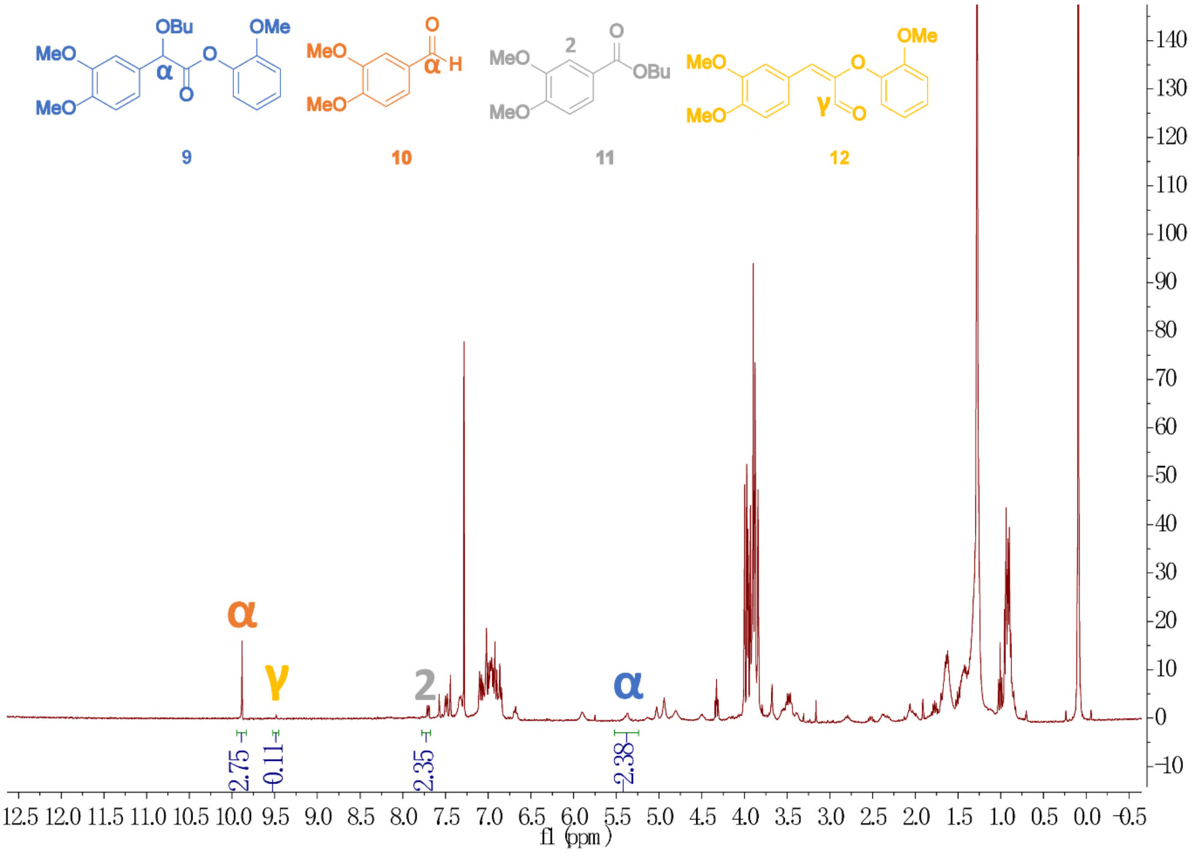

Figure S12: Quantitative ^{1}H NMR spectrum ($CDCl_3$) for the oxidation of **8** with modified Baker's conditions: CuCl (10 mol%), TEMPO (1.0 eq.), O_2 in pyridine for 9 hours (**Figure S11**, **entry 2**).

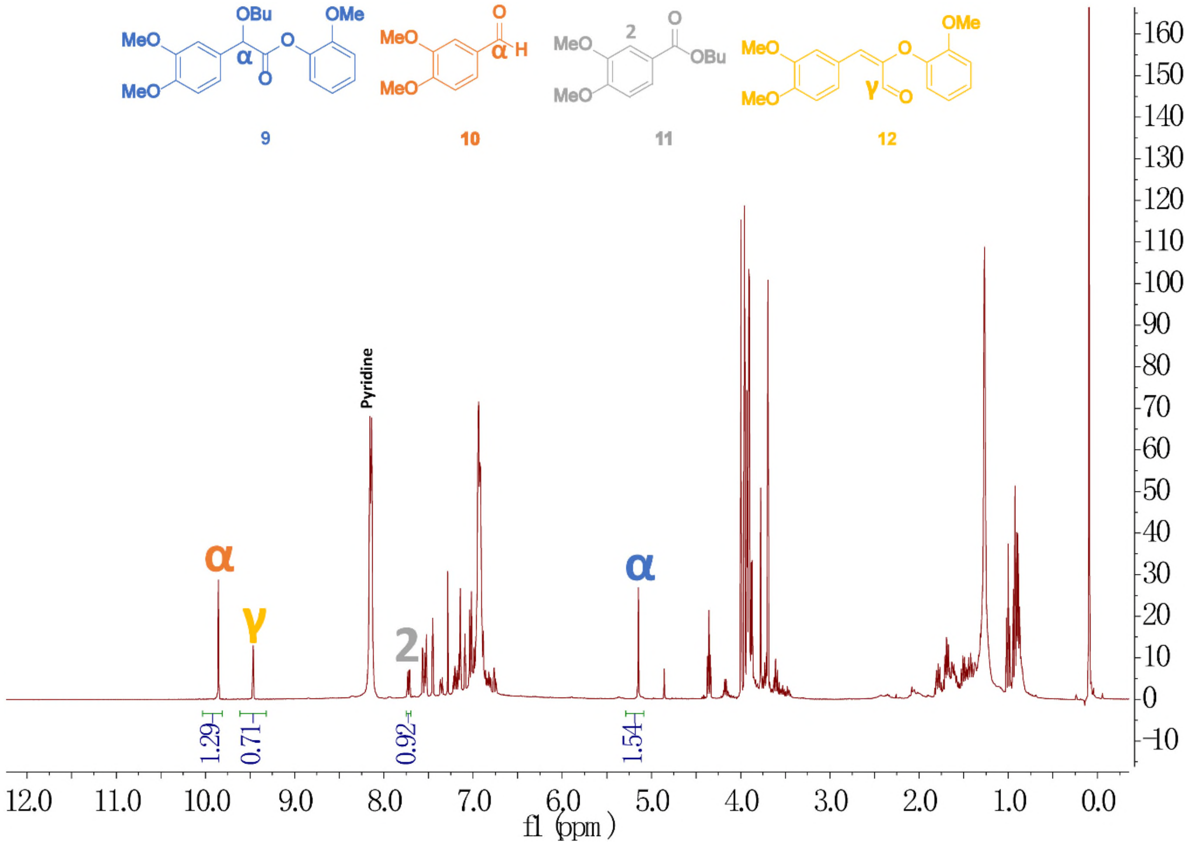

Figure S13: Quantitative ^{1}H NMR spectrum ($CDCl_3$) for the oxidation of **8** with modified Baker's conditions: CuBr (10 mol %), TEMPO (10 mol %), O_2 in pyridine for 9 hours (**Figure S11**, **entry 3**).

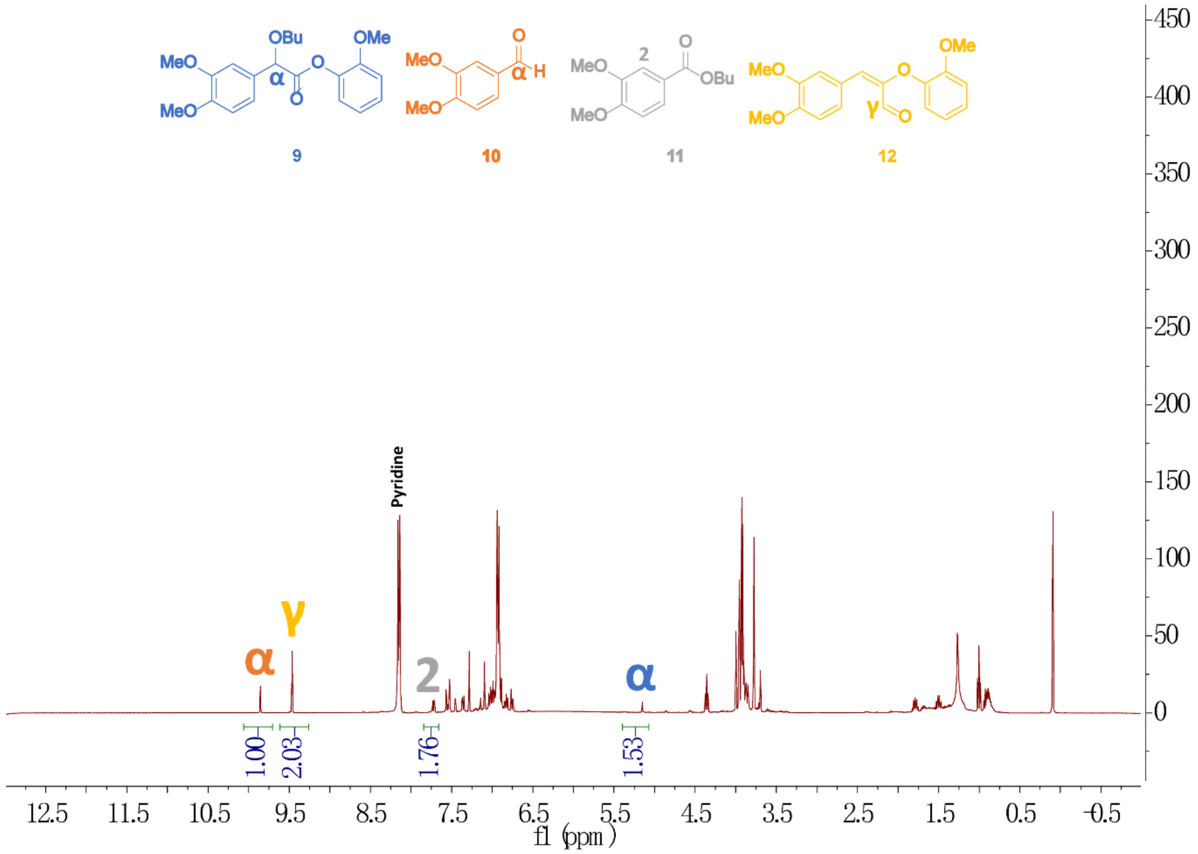

Figure S14: Quantitative ^{1}H NMR spectrum ($CDCl_3$) for the oxidation of **8** with modified Baker's conditions: CuOAc (10 mol %), TEMPO (10 mol %), O_2 in pyridine for 9 hours (**Figure S11**, **entry 4**).

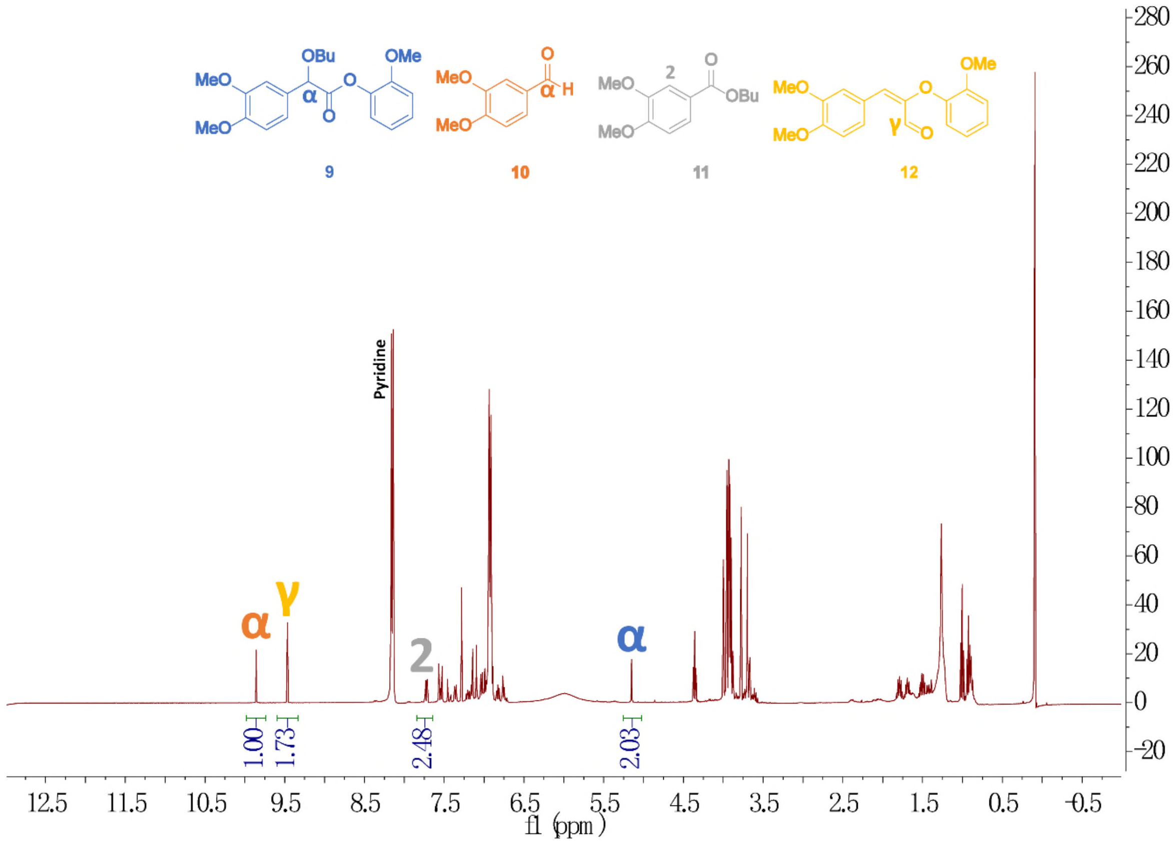

Figure S15: Quantitative ^{1}H NMR spectrum ($CDCl_3$) for the oxidation of **8** with modified Baker's conditions: CuOTf (10 mol %), TEMPO (10 mol %), O_2 in pyridine for 9 hours (**Figure S11**, **entry 5**).

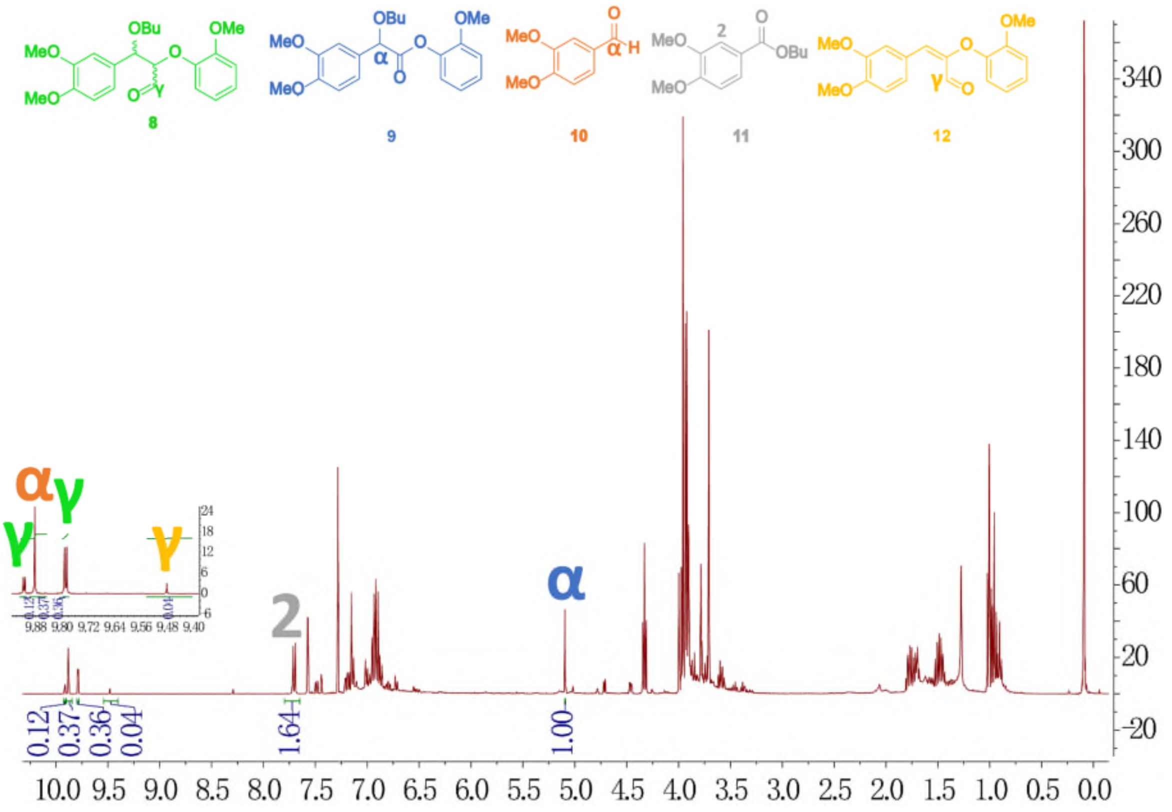

Figure S16: Quantitative ^{1}H NMR spectrum ($CDCl_3$) for the oxidation of **8** with modified Baker's conditions: CuCl (10 mol %), O_2 in pyridine for 9 hours (**Figure S11**, **entry 6**).

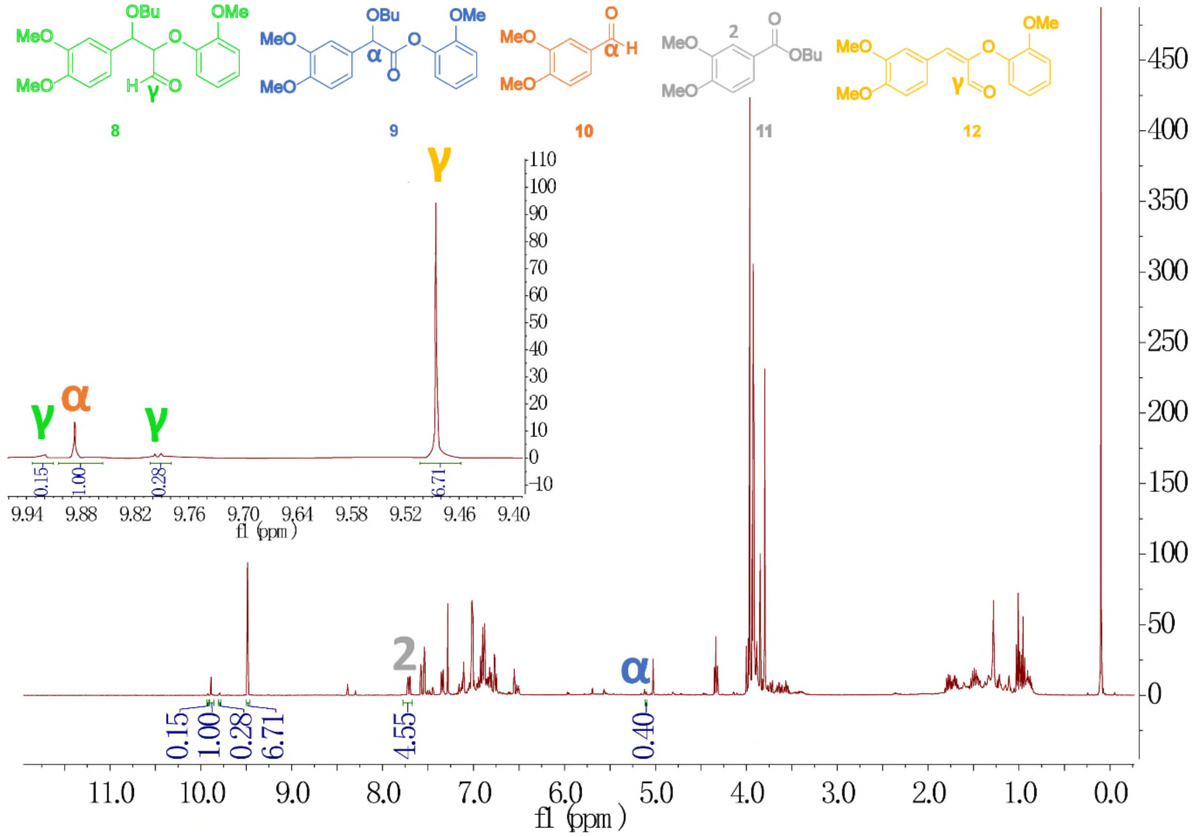

Figure S17: Quantitative ^{1}H NMR spectrum ($CDCl_3$) for the oxidation of **8** with modified Baker's conditions: TEMPO (10 mol %), O_2 in pyridine for 9 hours (**Figure S11**, **entry 7**).

5. General procedure for the Baker oxidation protocol[S3]:

Butanosolv β-O-4 γ-aldehyde lignin dimer **8** was prepared as previously reported.[S1]

To a flame-dried round bottom flask was added CuCl (1.0 mg, 0.01 mmol, 10 mol %), 0.1 mL of a fresh stock solution of TEMPO (0.1 M, 0.01 mmol, 10 mol %) in pyridine, 0.5 mL of a standard solution of lignin model compound **8** (0.2 M, 0.1 mmol, 100 mol %) in pyridine, followed by the addition of 0.4 mL pyridine, giving the final concentration of **8** as 0.1 M. Oxygen was bubbled through the reaction mixture for 1 minute and the flask was then sealed with a septum and an O_2 balloon added. The reaction was stirred at 100 °C for 9 hours. After cooling to rt., the solvent was evaporated, and 1 mL of a standard solution of 1,3,5-trimethoxybenzene in CH_3CN (10.8 mg/mL, 0.0599 M) was added and the resulting mixture was passed through a short silica column followed by washing with DCM/acetone (1:1, 10 mL). The eluent was concentrated *in vacuo* and the crude mixture was subjected to quantitative ^{1}H NMR analysis. Full spectra shown above in **Figures S10**.

Oxidation of 8 using Catalytic CuCl/TEMPO/O_2: the general protocol above was used with **8** (150 mg, 0.39 mmol, 100 mol %) in pyridine (2 mL), CuCl (4 mg, 0.039 mmol, 10 mol %) and TEMPO (6 mg, 0.039 mmol, 10 mol %). After cooling the reaction to rt., the solvent was evaporated and the residue was subjected to column chromatography (10 % EtOAc/Hex to 50 % EtOAc/Hex) to give **9** (45 mg, 0.12 mmol, 31 %) as a pale yellow oil, **10** (8 mg, 0.05 mmol, 12 %) as a white solid and **11** (33 mg, 0.14 mmol, 36 %) as a white solid.

2-Methoxyphenyl 2-butoxy-2-(3,4-dimethoxyphenyl) acetate (9): HRMS (ESI) calculated for $C_{21}H_{30}O_6N_1$ 392.2068, [M + NH_4]$^+$, found 392.2070; **IR** (thin film): 1770, 1606, 1593, 1514, 1498, 1138, 1024, 829, 758 cm^{-1}; **^{1}H NMR** (500 MHz, $CDCl_3$) δ 7.22 – 7.13 (m, 3H, H10, H11, H12), 6.96 – 6.87 (m, 4H, H2, H5, H6, H9), 5.10 (s, 1H, Hα), 3.94 (s, 3H, OC**H₃**), 3.92 (s, 3H, OC**H₃**), 3.73 (dt, *J* = 9.1, 6.7 Hz, 1H, 1 x OC**H₂**CH₂), 3.71 (s, 3H, OCH₃), 3.59 (dt, *J* = 9.1, 6.7 Hz, 1H, 1 x OC**H₂**CH₂), 1.76 – 1.67 (m, 2H, CH₂C**H₂**), 1.52 – 1.41 (m, 2H, C**H₂**CH₃), 0.96 (t, *J* = 7.4 Hz, 3H, CH₂C**H₃**). **^{13}C NMR** (126 MHz, $CDCl_3$) δ 169.4 (Cβ), 151.0 (C8), 149.3 (C3), 149.0 (C4), 139.5 (C7), 129.0 (C1), 127.0 (C10), 122.5 (C6), 120.7 (C11), 120.4 (C12), 112.5 (C9), 110.8 (C5), 110.1 (C2), 80.7 (Cα), 69.8 (O**C**H₂CH₂), 56.0 (O**C**H₃), 55.9 (O**C**H₃), 55.7 (O**C**H₃), 31.7 (OCH₂**C**H₂), 19.3 (**C**H₂CH₃), 13.9 (CH₂**C**H₃).

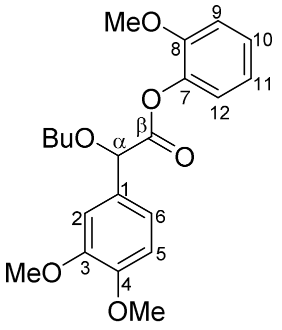

3,4-Dimethoxybenzaldehyde (10)[S4]: m.p. 42-45 °C (lit.[S4] 43-45 °C); **^{1}H NMR** (500 MHz, $CDCl_3$) δ 9.89 (s, 1H, C**H**O), 7.49 (dd, *J* = 8.2, 1.9 Hz, 1H, **H**6), 7.44 (d, *J* = 1.9 Hz, 1H, **H**2), 7.01 (d, *J* = 8.2 Hz, 1H, **H**5), 4.00 (s, 3H, OC**H₃**), 3.98 (s, 3H, OC**H₃**). Analytical data was in accordance with that previously reported.[S4]

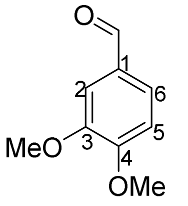

Butyl 3,4-dimethoxybenzoate (11)[S5]: m.p. 150-152 °C (lit.[S5] 153-154 °C); **^{1}H NMR** (500 MHz, $CDCl_3$) δ 7.70 (dd, *J* = 8.4, 2.0 Hz, 1H, H6), 7.57 (d, *J* = 2.0 Hz, 1H, H2), 6.91 (d, *J* = 8.4 Hz, 1H, H5), 4.32 (t, *J* = 6.6 Hz, 2H, OC**H₂**CH₂), 3.96 (s, 3H, OC**H₃**), 3.96 (s, 3H, OC**H₃**) 1.81 – 1.73 (m, 2H, OCH₂C**H₂**), 1.54 – 1.45 (m, 2H, C**H₂**CH₃), 1.00 (t, *J* = 7.4 Hz, 3H, CH₂C**H₃**). Analytical data was in accordance with that previously reported.[S5]

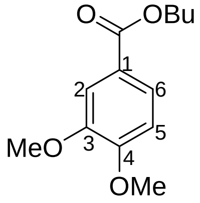

During the course of these studies, the original Baker protocol[S3] was also attempted using the S-G model compound **13**. Overall, the results of this experiment were similar to the reaction of **13** under analogous conditions but in the absence of TEMPO (see **Figure 2A entry 4** in manuscript).

Oxidation of 13 by using Catalytic CuCl/TEMPO/O_2: The general protocol above was used with **13** (150mg, 0.36 mmol,

100 mol %) in pyridine (2 mL), CuCl (4 mg, 0.036 mmol, 10 mol%) and TEMPO (6 mg, 0.036 mmol, 10 mol%). After cooling the reaction to rt, the solvent was evaporated and the residue was subjected to column chromatography (10% EtOAc/Hex to 50% EtOAc/Hex) to produce **14** (40 mg, 0.10 mmol, 28 %) as a light-yellow oil, **15** (11 mg, 0.06 mmol, 16 %) as a colourless oil, **16** (48 mg, 0.18 mmol, 40 %) as a colourless oil and **17** (14 mg, 0.04 mmol, 10 %) as a light yellow solid.

2-Methoxyphenyl 2-butoxy-2-(3,4,5-trimethoxyphenyl) acetate (14). **HRMS** (ESI) calculated for $C_{22}H_{28}O_7Na$ 427.1733, $[M + Na]^+$, found 427.1720; **IR** (thin film): 1737, 1598, 1573, 1510, 1483, 1130, 1031, 879, 773 cm^{-1}. **^{1}H NMR** (500 MHz, $CDCl_3$) δ 7.20 (ddd, J = 8.3, 7.3, 1.8 Hz, 1H, H10), 7.00 – 6.89 (m, 3H, H9, H11, H12), 6.84 (s, 2H, H2 and H6), 5.08 (s, 1H, Hα), 3.90 (s, 6H, 2 × OCH_3), 3.88 (s, 3H, OCH_3), 3.79 (dt, J = 9.1, 6.7 Hz, 1H, 1 x OC**H$_2$**CH_2), 3.70 (s, 3H, OCH_3), 3.61 (dt, J = 9.1, 6.7 Hz, 1H, 1 x OC**H$_2$**CH_2), 1.78 – 1.68 (m, 2H, OCH_2C**H$_2$**), 1.52 – 1.43 (m, 2H, C**H$_2$**CH_3), 0.97 (t, J = 7.4 Hz, 3H, CH_2C**H$_3$**). **^{13}C NMR** (126 MHz, $CDCl_3$) 169.2 (Cβ), 153.3 (C3 and C5), 151.0 (C8), 139.5 (C7), 138.1 (C4), 132.0 (C1), 127.1 (C10), 122.5 (C11) , 120.7 (C12), 112.5 (C9) , 104.4 (C2 and C6), 81.0 (Cα), 70.1 (O**C**H_2CH_2), 60.8 (OCH_3), 56.2 (2 × O**C**H_3), 55.7 (OCH_3), 31.5 (OCH_2**C**H_2), 19.5 (**C**H_2CH_3), 13.9 (CH_2**C**H_3).

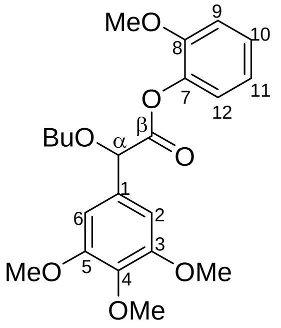

3,4,5-trimethoxybenzaldehyde (15)[S6]. **m.p**. 69-72 °C (lit.[S6] 71 – 72 °C), **^{1}H NMR** (500 MHz, $CDCl_3$) δ 9.88 (s, 1H, -C**H**O), 7.14 (s, 2H, H2 and H6), 3.95 (s, 3H, OC**H$_3$**), 3.95 (s, 6H, 2 × OC**H$_3$**). Analytical data was in accordance with that previously reported. [S6]

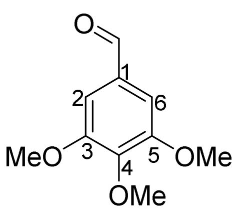

Butyl 3,4,5-trimethoxybenzoate (**16**). **HRMS** (ESI) calculated for $C_{14}H_{20}O_6Na$ 291.1208, $[M + Na]^+$, found 291.1198; **IR** (thin film): 2926, 1710, 1328, 1213, 1120, 1004, 950, 798, 758 cm^{-1}; **^{1}H NMR** (500 MHz, $CDCl_3$) δ 7.32 (s, 2H, H2 and H6), 4.34 (t, J = 6.7 Hz, 2H, OC**H$_2$**CH_2), 3.98 – 3.84 (m, 9H, 3 × OCH_3), 1.77 (dq, J = 8.7, 6.8 Hz, 2H, OCH_2C**H$_2$**), 1.53 – 1.45 (m, 2H, C**H$_2$**CH_3), 1.00 (t, J = 7.4 Hz, 3H, CH_2C**H$_3$**); **^{13}C NMR** (126 MHz, $CDCl_3$) 166.3 (**C**=O), 152.9 (C3 and C5), 142.0 (C4), 125.6 (C1), 106.7 (C2 and C6), 65.1 (O**C**H_2CH_2), 60.9 (O**C**H_3), 56.2 (2 × O**C**H_3), 30.8 (OCH_2**C**H_2), 19.3 (**C**H_2CH_3), 13.8 (CH_2**C**H_3).

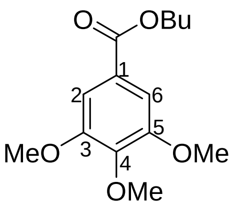

(*E*)-2-(2-methoxyphenoxy)-3-(3,4,5-trimethoxyphenyl) acrylaldehyde (17). HRMS (ESI) calculated for $C_{19}H_{20}O_6Na$, $[M + Na]^+$ 367.1158, found 367.1145; **IR** (thin film): 2933, 2837, 1674, 1576, 1456, 1250, 1005, 827, 791, 750 cm^{-1}; **m.p**. 111-113 ° C; **^{1}H NMR** (500 MHz, $CDCl_3$) δ 9.49 (s, 1H, Hγ), 7.11 (s, 2H, H2 and H6), 7.05 – 6.95 (m, 3H, H8, H9 and Hα), 6.81 (ddd, J = 8.0, 7.0, 2.0 Hz, 1H, H10), 6.74 (dd, J = 8.0, 1.5 Hz, 1H, H11), 3.93 (s, 3H, OC**H$_3$**), 3.88 (s, 3H, OC**H$_3$**), 3.80 (s, 6H, 2 × OC**H$_3$**); **^{13}C NMR** (126 MHz, $CDCl_3$) δ 187.3 (Cγ), 153.1 (C3 and C5), 148.9 (C7), 148.2 (Cβ), 144.8 (C12), 140.3 (C4), 135.5 (Cα), 127.5 (C1), 123.3 (C9), 120.9 (C10), 114.5 (C11), 112.5 (C8), 108.1 (C2 and C6), 61.0 (O**C**H_3), 56.0 (O**C**H_3), 55.9 (2 × OC**H$_3$**). The stereochemistry of **17** has been tentatively assigned based on that of known compound **12**.[S1]

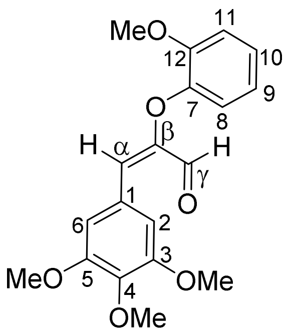

6. Oxidation of lignin model compounds 8, 13 and 18 using modified Baker's conditions: catalytic CuCl under an Oxygen atmosphere in the absence of TEMPO

6.1. General procedure

Butanosolv β-O-4 γ-aldehyde lignin dimers **8**, **13** and **18** were prepared as previously reported.[S1]

To a small flame-dried round bottom flask was added CuCl (1.0 mg, 0.01 mmol, 10 mol %), 0.5 mL of a standard solution of lignin model compound (0.2 M, 0.1 mmol, 100 mol %) in pyridine, followed by the addition of 0.5 mL pyridine, to achieve a final concentration of substrate of 0.1 M [Caution: pyridine should be freshly distilled]. Oxygen was bubbled through the reaction mixture for 1 minute and the flask was then sealed with a septum and an O_2 balloon inserted. The reaction was stirred at 100 °C for the required number of hours. After cooling to rt, 1 mL of a standard solution of 1,3,5-trimethoxybenzene in CH_3CN (10.8 mg/mL, 0.0599 M) was added and the resulting mixture was passed through a short silica column followed by washing with DCM/ acetone (1:1, 10 mL). The filtrate was concentrated *in vacuo* and the crude mixture was subjected to quantitative ^1H NMR analysis. Full spectra are shown in **Figures S18-S22.**

The spectra used to generate the bar chart **Figure 1B**, **entries 5-9** in manuscript are shown in **Figures S18-S22**.

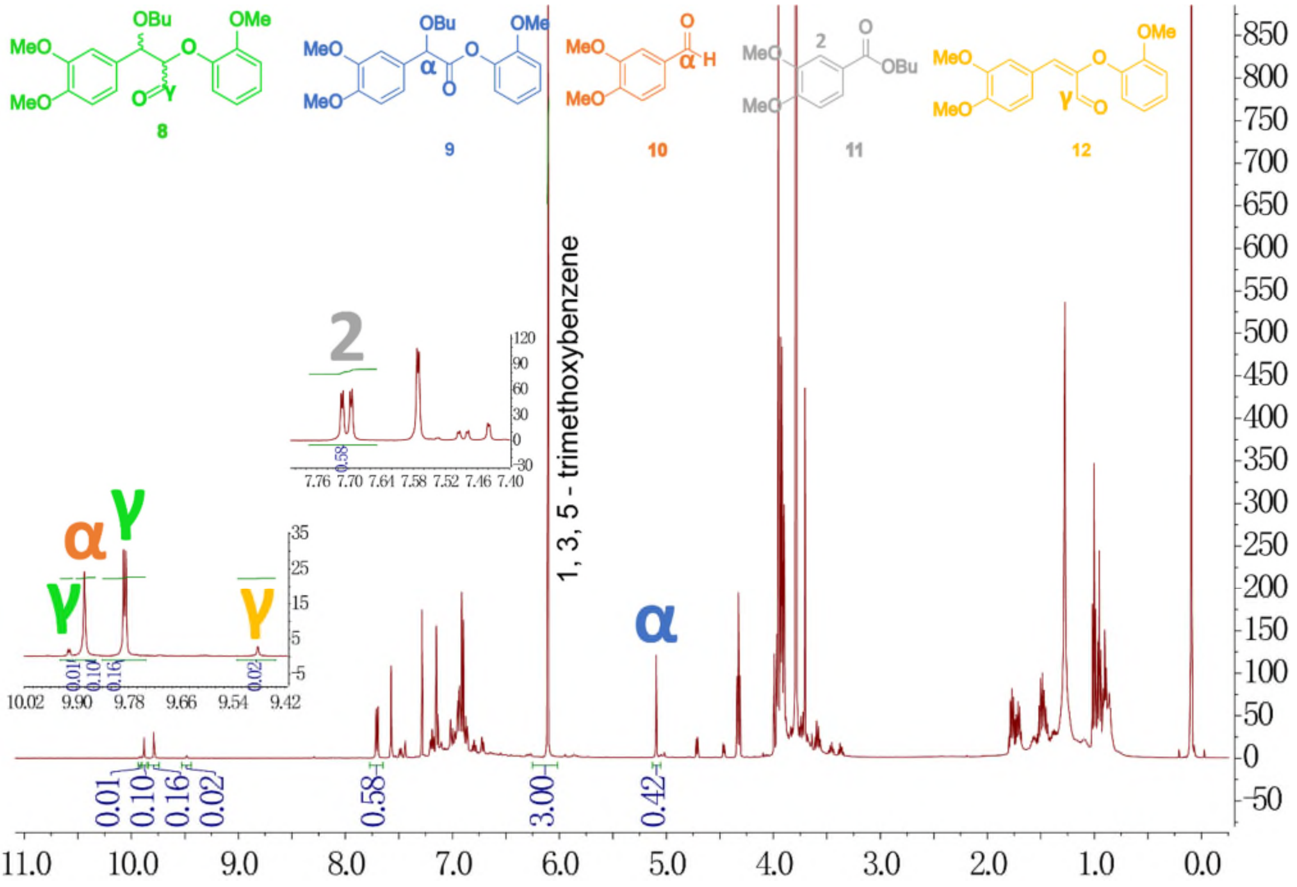

Figure S18: Quantitative ^1H NMR spectrum ($CDCl_3$) for the oxidation of **8** with modified Baker's conditions: CuCl (0.1 eq.), O_2 in pyridine in the absence of TEMPO at 100 °C for 9 hours (**Figure 1B, entry 5** in manuscript).

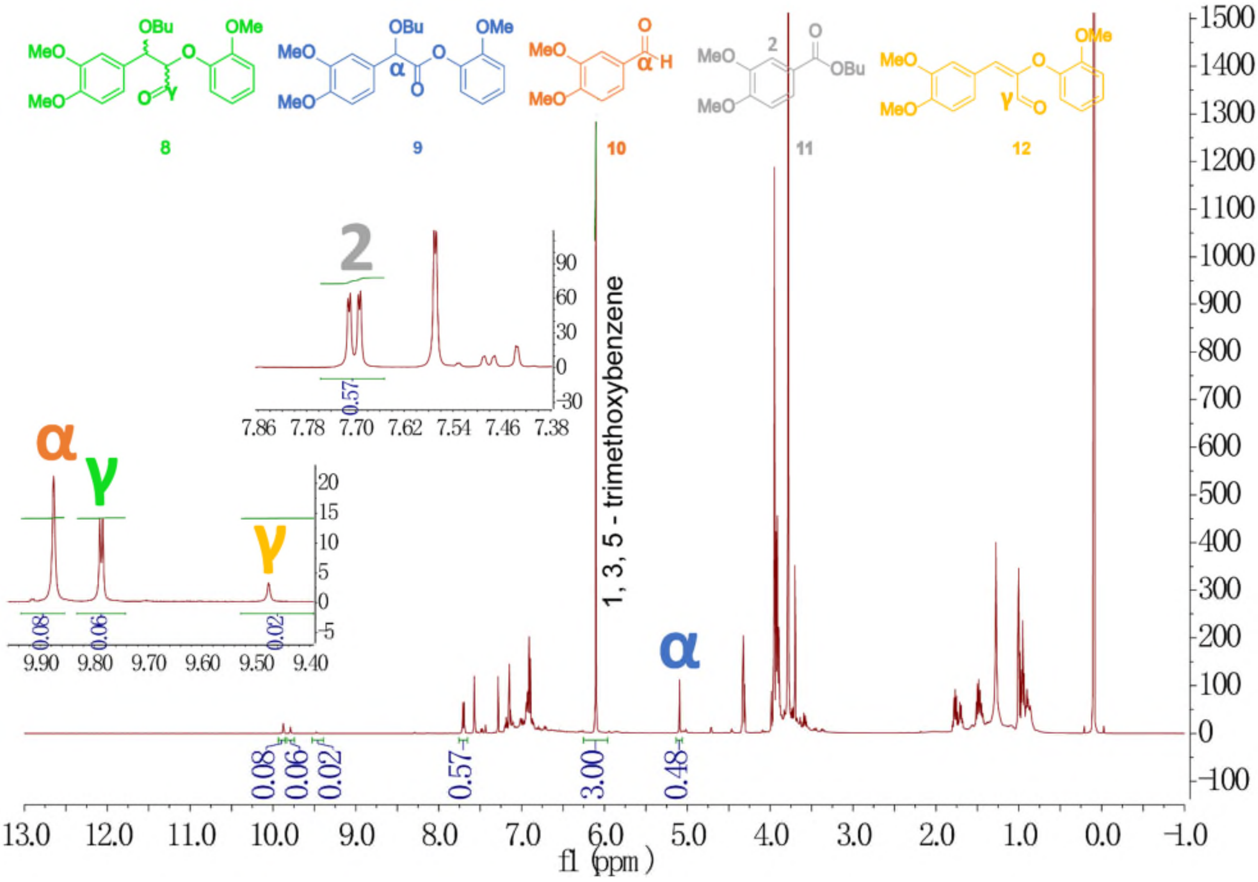

Figure S19: Quantitative ^{1}H NMR spectrum ($CDCl_3$) for the oxidation of **8** with modified Baker's conditions[S3]: CuCl (0.1 eq.), O_2 in pyridine in the absence of TEMPO at 100 °C for 15 hours (**Figure 1B, entry 6** in manuscript).

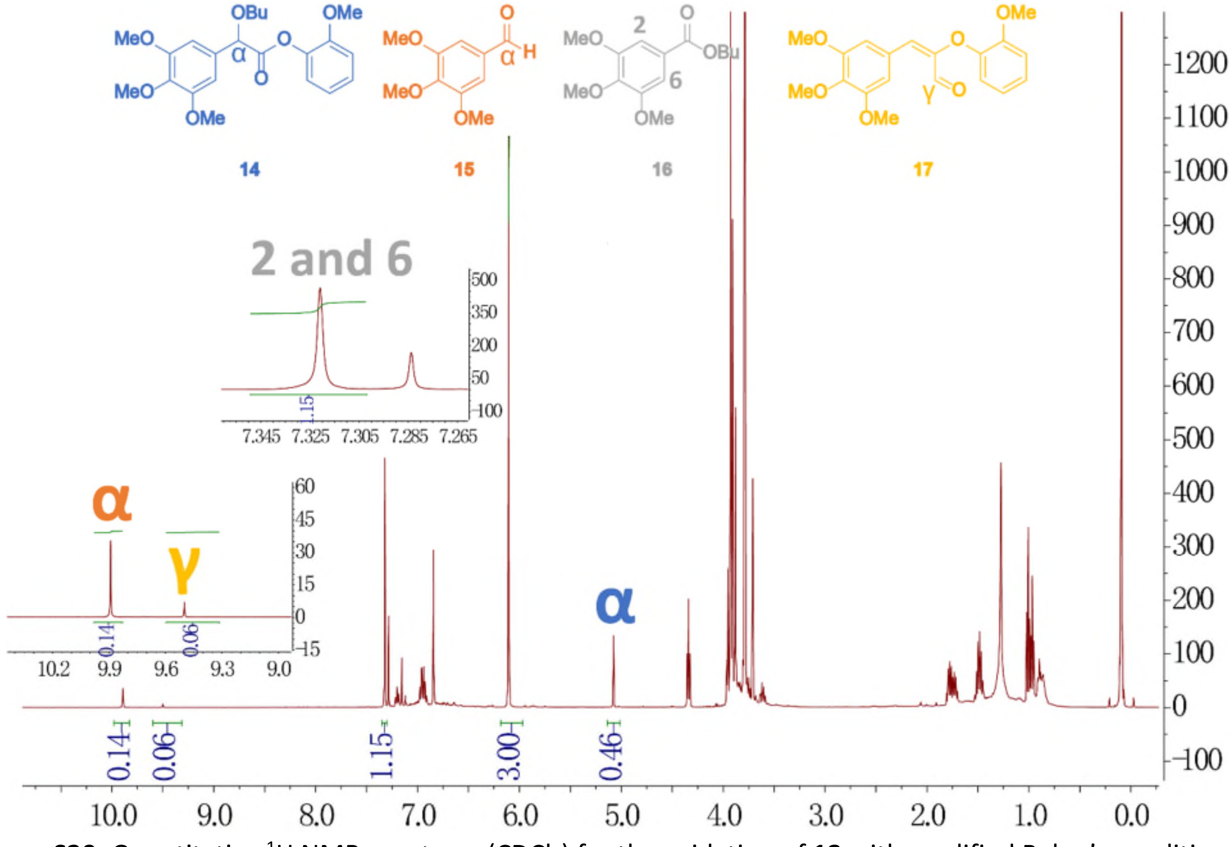

Figure S20: Quantitative ^{1}H NMR spectrum ($CDCl_3$) for the oxidation of **13** with modified Baker's conditions[S3]: CuCl (0.1 eq.), O_2 in pyridine in the absence of TEMPO at 100 °C for 15 hours (**Figure 1B, entry 7** in manuscript).

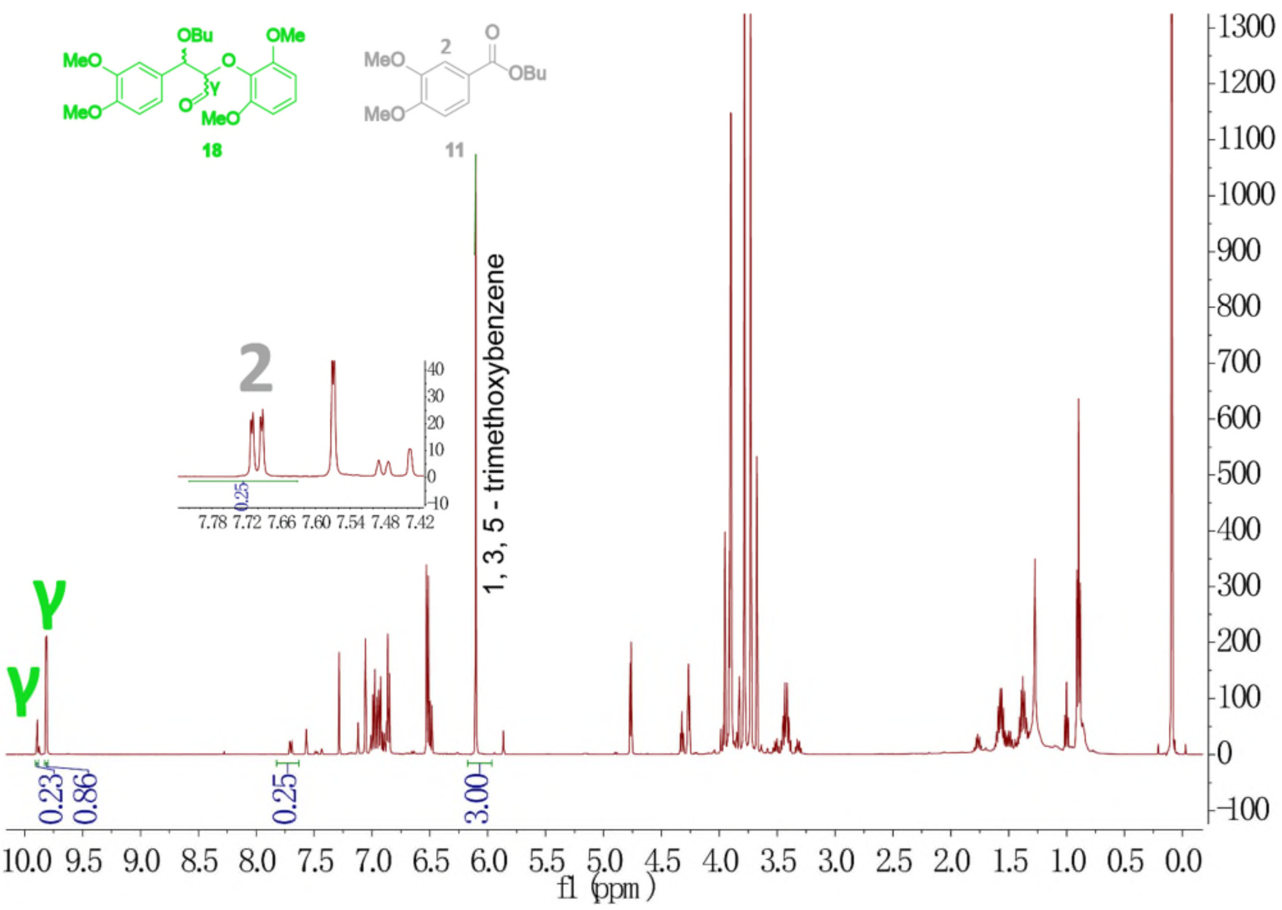

Figure S21: Quantitative ^{1}H NMR spectrum ($CDCl_3$) for the oxidation of **18** with modified Baker's conditions[S3]: CuCl (0.1 eq.), O_2 in pyridine in the absence of TEMPO at 100 °C for 15 hours (**Figure 1B, entry 8** in manuscript).

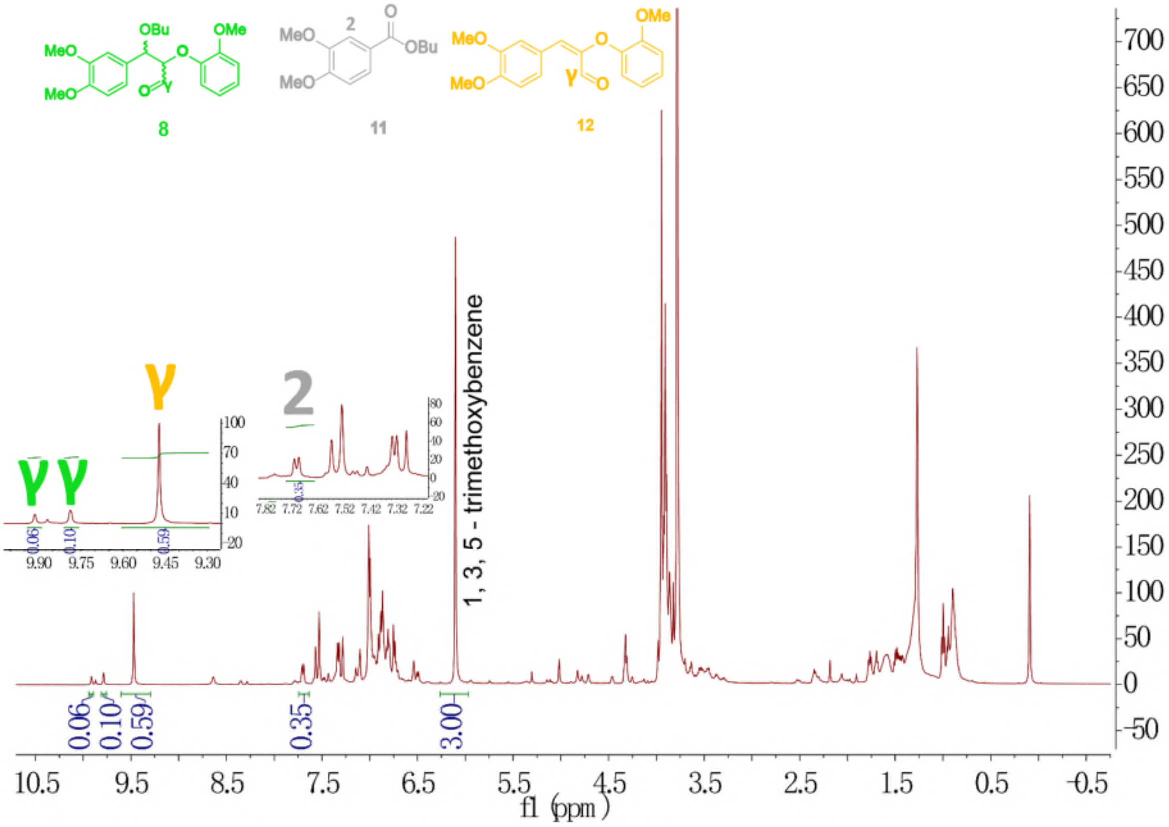

Figure S22: Quantitative ^{1}H NMR spectrum ($CDCl_3$) for the oxidation of **8** with modified Baker's conditions[S3]: O_2 in pyridine in the absence of TEMPO and CuCl at 100 °C for 15 hours (**Figure 1B, entry 9** in manuscript).

6.2. Two possible mechanisms for the formation of butyl ester 11 from 8 using catalytic CuCl under an

oxygen atmosphere in the absence of TEMPO.

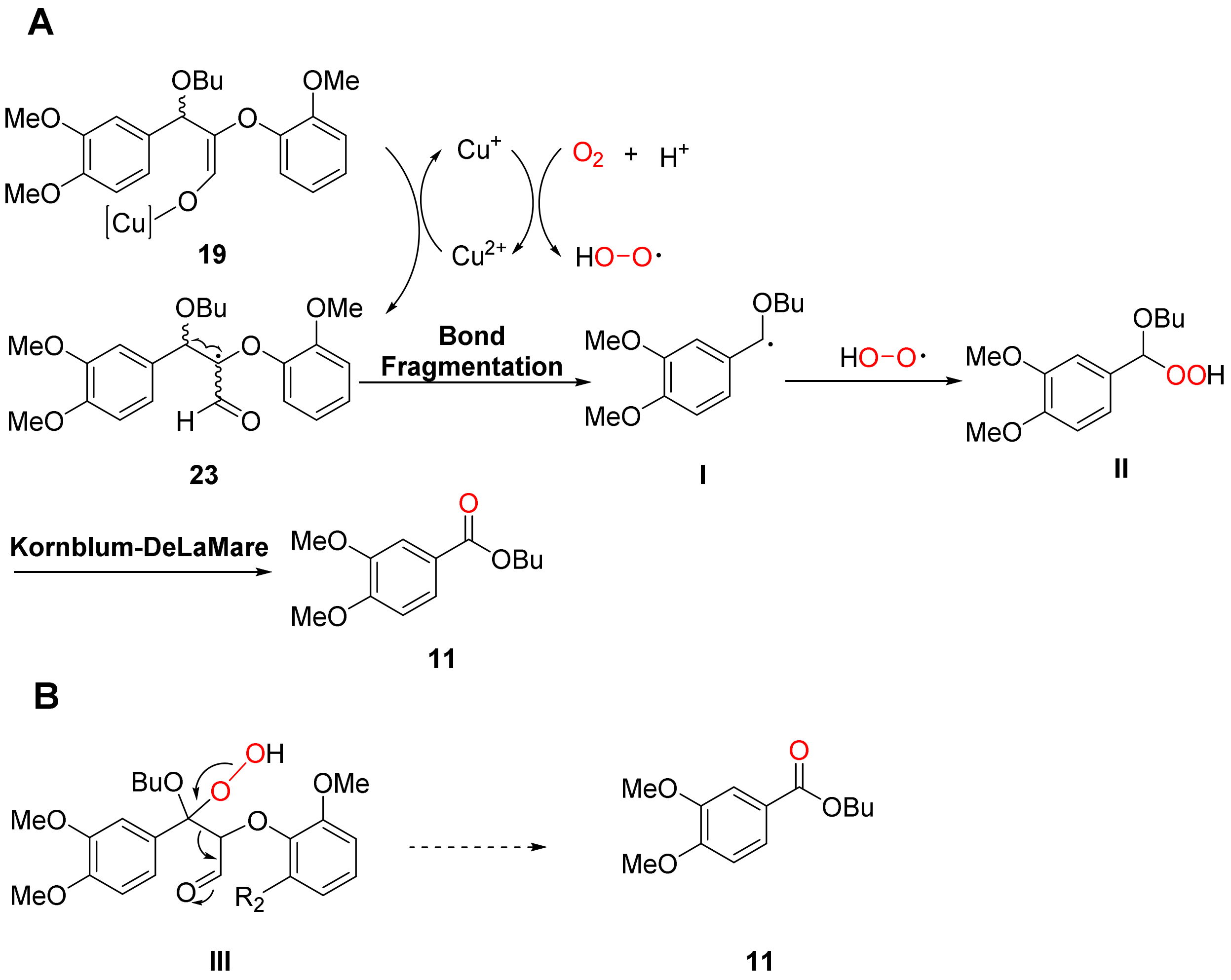

Scheme S2: A An oxidation of enolate **19** by Cu (**II**) leads to the formation of radical **23** (**Scheme 2** in manuscript), which can then fragment to give radical **I**. Radical-radical combination with the superoxide radical leads to the formation of **II** which undergoes a Kornblum-DeLaMare fragmentation[S7] to give **11**. This mechanism is based on a proposal from Stephenson *et al.*[S8] **B** According to the results presented in **Figure 1B, entry 9** in the manuscript, butyl ester **11** was still formed in the apparent absence of CuCl. Whilst it remains possible that trace amounts of CuCl were present in these control reactions[S9], the formation of intermediate **III** possibly from a precursor α-anion or α-radical could lead to **11** as shown.

6.3. Possible mechanism for the formation of 10 from 8 using catalytic CuCl under Oxygen atmosphere in the absence of TEMPO.

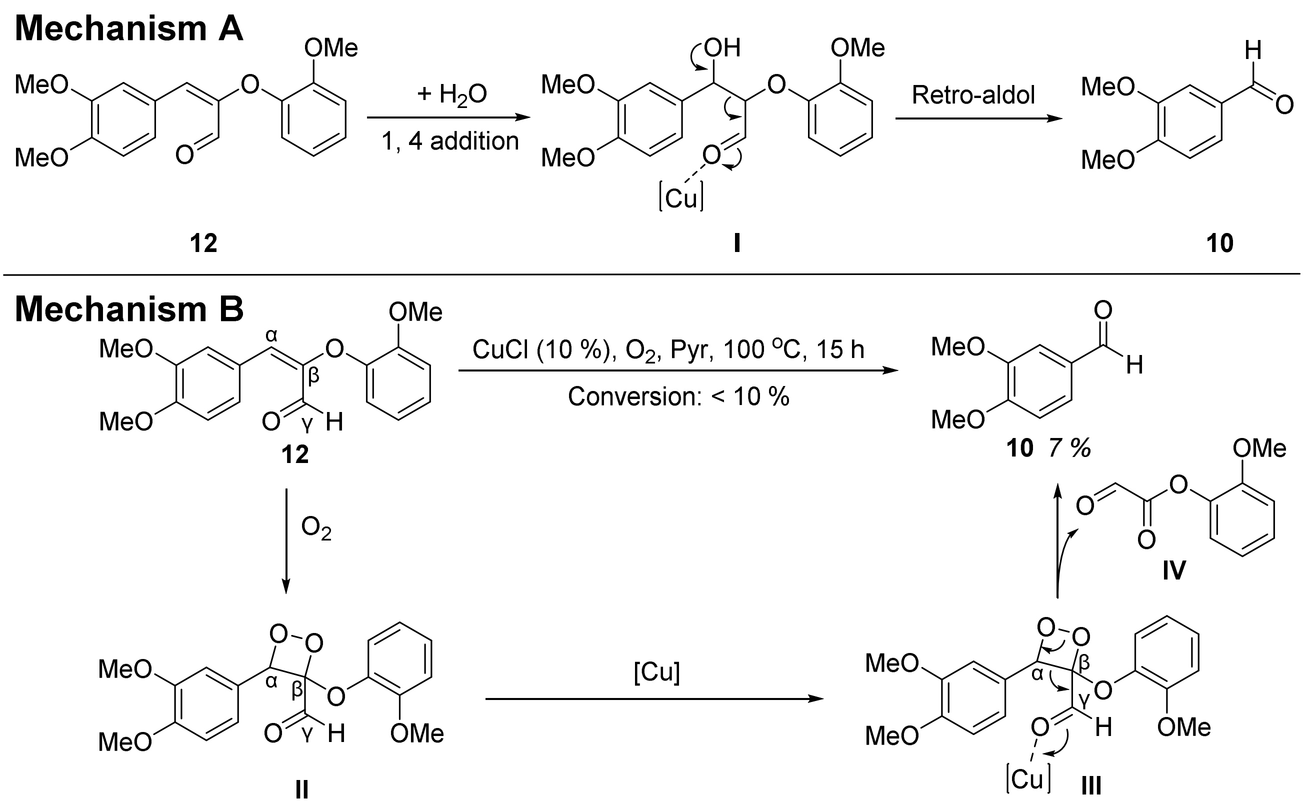

Scheme S3: Possible mechanism **A** for the formation of **10**: 1,4-addition of H_2O to enal **12** to form intermediate **I** followed by a rapid retro-aldol reaction with the help of copper-mediated Lewis acid catalysis.[S2] Possible mechanism **B** for the formation of **10**: oxidation of enal **12** using catalytic CuCl under an oxygen atmosphere gives aldehyde **10**. Reactions were conducted on a 0.1 mmol scale following the general procedure **6.1**. Yields were determined by ^{1}H NMR by using 1,3,5-trimethoxybenzene as the internal standard (**Figure S23**). A possible mechanism is that reaction with molecular oxygen generates an unstable dioxetane intermediate **I**. The activation of the γ-carbonyl by Cu could lead to the further polarization of the C_α-C_β bond aiding collapse of the dioxetane to form **10**. This mechanism is based on a proposal from Sperry *et al*[S10].

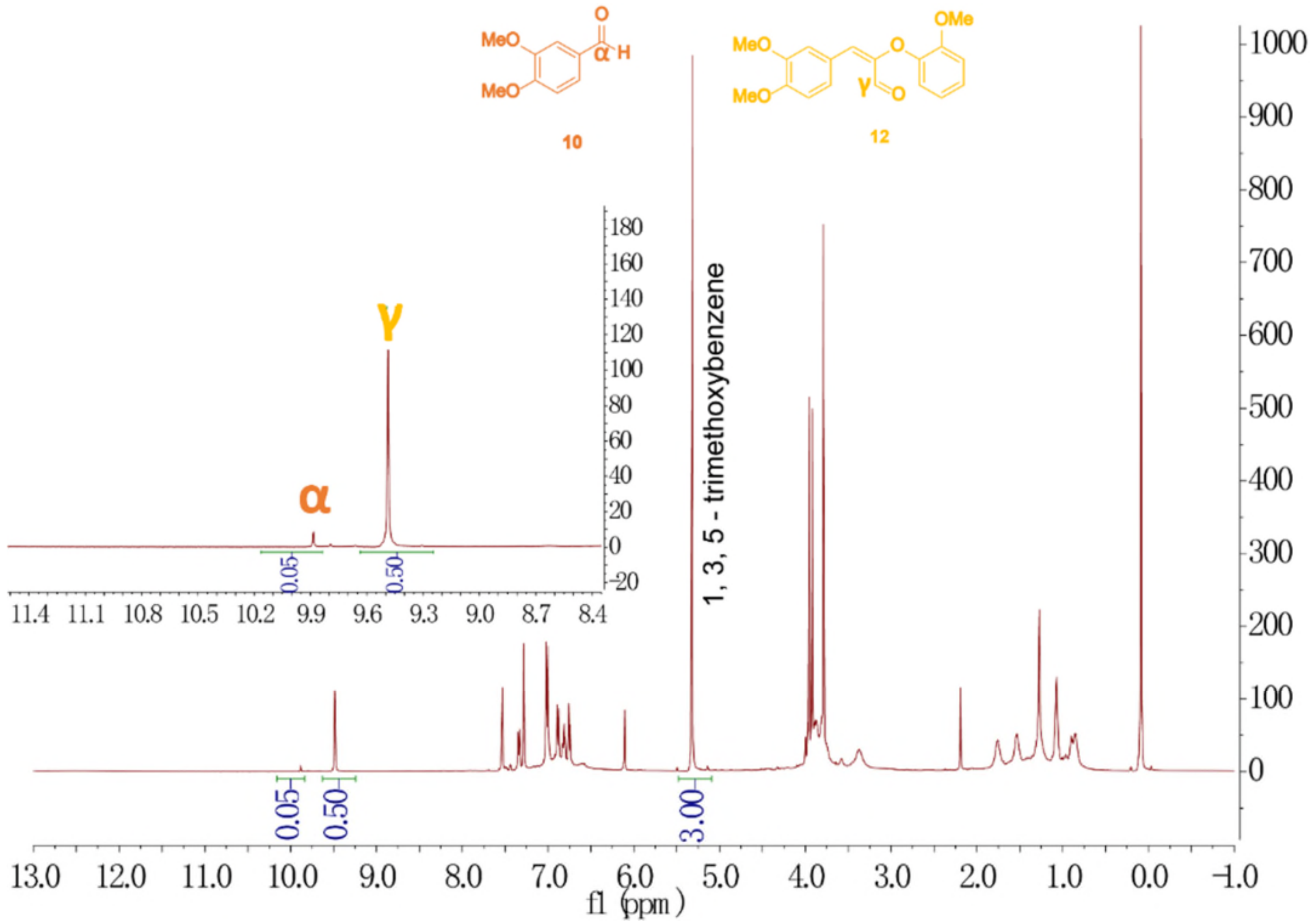

Figure S23: Quantitative ^{1}H NMR spectrum ($CDCl_3$) for the oxidation of **12** with modified Baker's conditions: CuCl (0.1 eq.), O_2 in pyridine in the absence of TEMPO at 100 °C for 15 hours. (data relevant to the discussion in **Scheme S3**)

7. Oxidation of lignin model compounds 8, 13, 18 and 21 using catalytic CuCl/ stoichiometric TEMPO under Ar atmosphere

7.1. General procedure

Butanosolv β-O-4 γ-aldehyde lignin dimers **8**, **13**, **18** and **21** were prepared as previously reported.[S1]

To a small flame dried Schlenk flask under an argon atmosphere was added CuCl (1.0 mg, 0.01 mmol, 10 mol %). The flask was then sealed with a septum and connected to the high vacuum for 5 min to remove any remaining air, after which the flask was filled using an Ar balloon. 0.5 mL of a fresh standard solution of lignin model compound (0.2 M, 0.1 mmol, 100 mol%) in pyridine and 0.3 mL of fresh stock solution of TEMPO (1.0 M, 0.3 mmol, 300 mol%) were added via syringe, followed by the addition of 0.2 mL pyridine, to achieve a final concentration of substrate of 0.1 M [Caution: pyridine should be freshly distilled and degassed]. The reaction was stirred at 100 °C for 9 hours. After cooling to rt, 1 mL of standard solution of 1,3,5-trimethoxybenzene in acetonitrile (10.8 mg/mL, 0.0599 M) was added and the solvent was evaporated. The residue was subjected to a short silica column eluting with 100% Hex (5 mL) to remove the excess TEMPO and the recovery of the crude reaction mixture was achieved by washing the column with 50% DCM/acetone (10 mL). The crude mixture was concentrated *in vacuo* and subjected to quantitative ^{1}H NMR analysis. Full spectra are shown in **Figures S26-S31**.

7.2. Oxidations using catalytic CuCl/ stoichiometric TEMPO under an Ar atmosphere

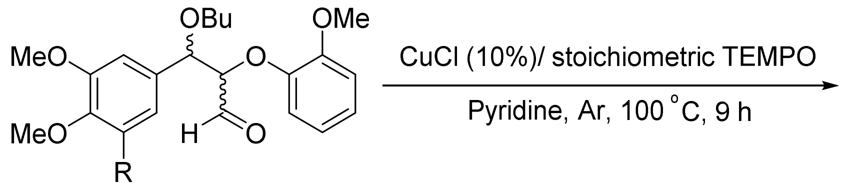

8, R = H
13, R = OMe

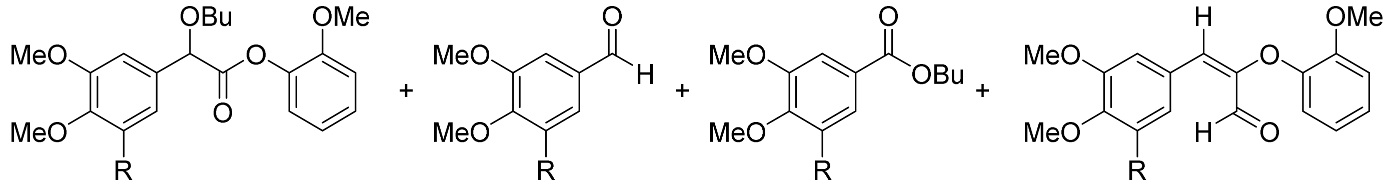

9, R = H
14, R = OMe

10, R = H
15, R = OMe

11, R = H
16, R = OMe

12, R = H
17, R = OMe

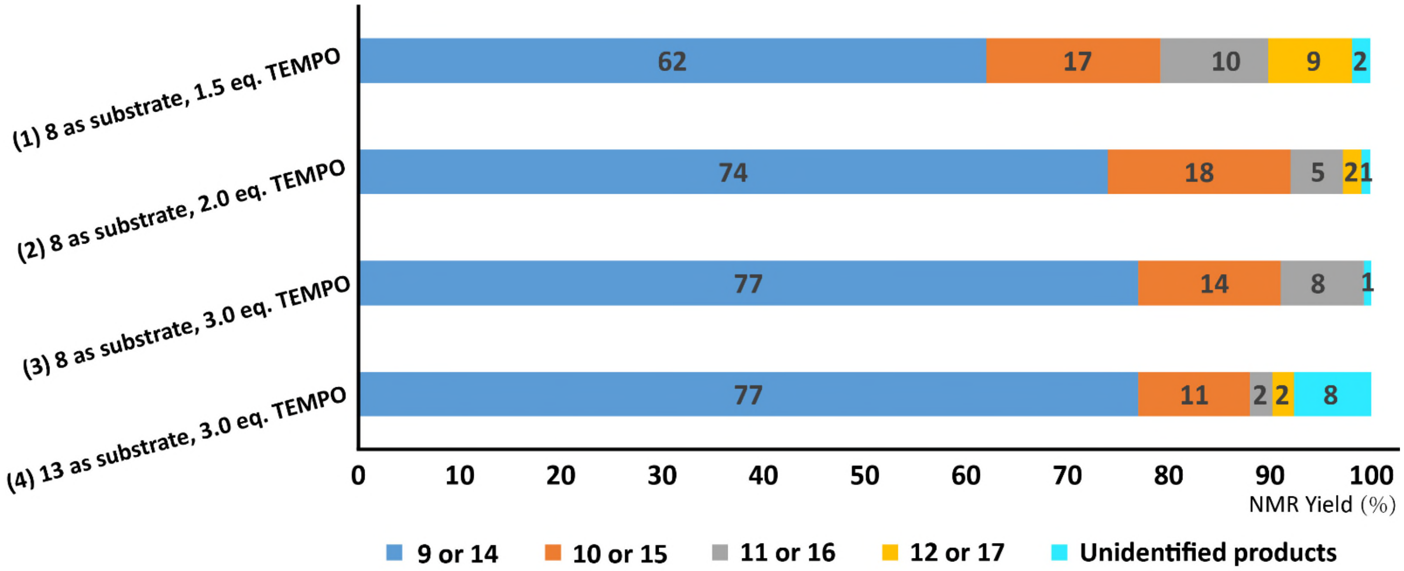

Figure S24: This figure provides additional details relating to the data presented in Figure 2 of the manuscript. The above results showed that a small amount of cleaved aldehyde (**10** or **15**) and butyl ester (**11** or **16**) were still formed when TEMPO functioned as the oxidant under an argon atmosphere. Theoretically, 2.0 eq. TEMPO was required to generate the desired aryl ester in 100 % yield (see proposed mechanism discussed in Scheme **3C** in the manuscipt). Comparing the result in **entry 3** with that in **entry 2**, the yield of aryl ester **9** did not increase despite the amount of TEMPO being increased from 2 eq. to 3 eq., indicating that there could be a competing reaction to form cleavage products **10** (or **15**) and **11** (**or 16**) that is independent of the concentrating of TEMPO used. One possibility is that any remaining oxygen and water could lead to the formation of cleavage products (see **Figure S25**). Reactions were conducted on a 0.1 mmol scale following the general procedure **7.1**. Yields were determined by ^{1}H NMR by using 1,3,5-trimethoxybenzene as the internal standard. Full spectrums shown in **Figure S26-S29.**

7.3. Background reaction by using catalytic CuCl under Ar atmosphere

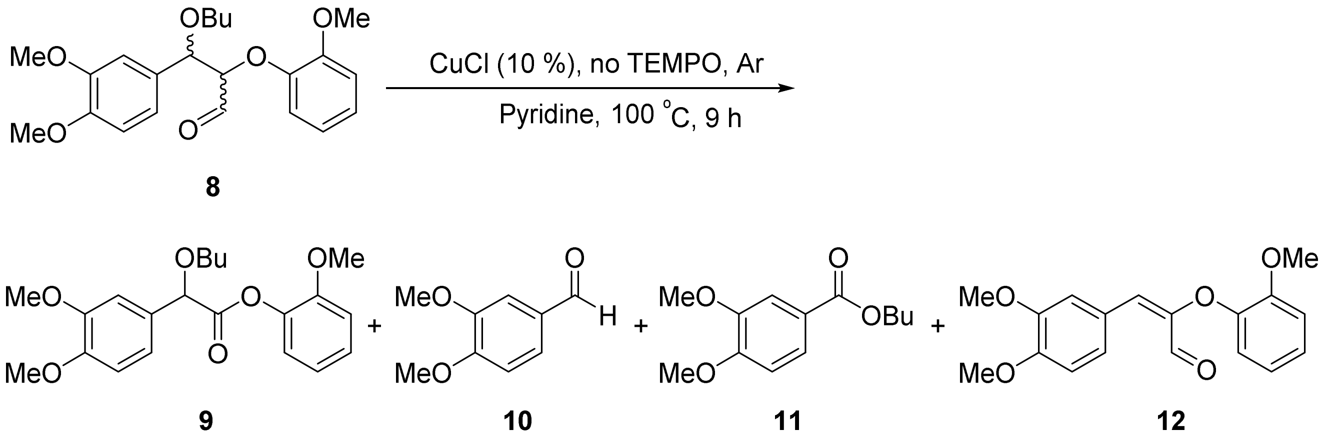

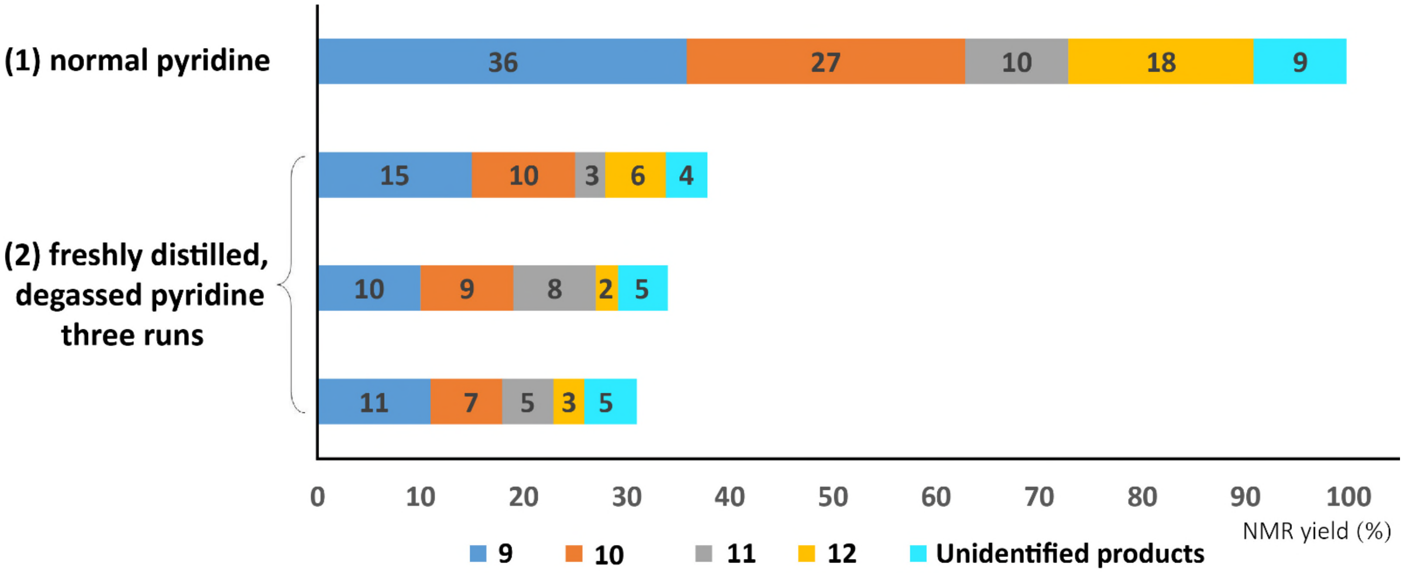

Figure S25: When the reaction was initially run apparently without TEMPO under an argon atmosphere but with CuCl, surprisingly all four products were still formed. When unpurified pyridine was used, 100 % conversion was observed (**entry 1**). When the pyridine was freshly distilled, degassed, and then used immediately, the background conversion was reduced significantly (triplicate, **entry 2**), but could not be fully eradicated. Based on these results, the cleavage products **10** and **11** could be formed in the background reaction. Reactions were conducted on a 0.1 mmol scale following the general procedure **7.1**. Yields were determined by ^{1}H NMR by using 1,3,5-trimethoxybenzene as the internal standard. Full spectra are shown in **Figures S32-S35.**

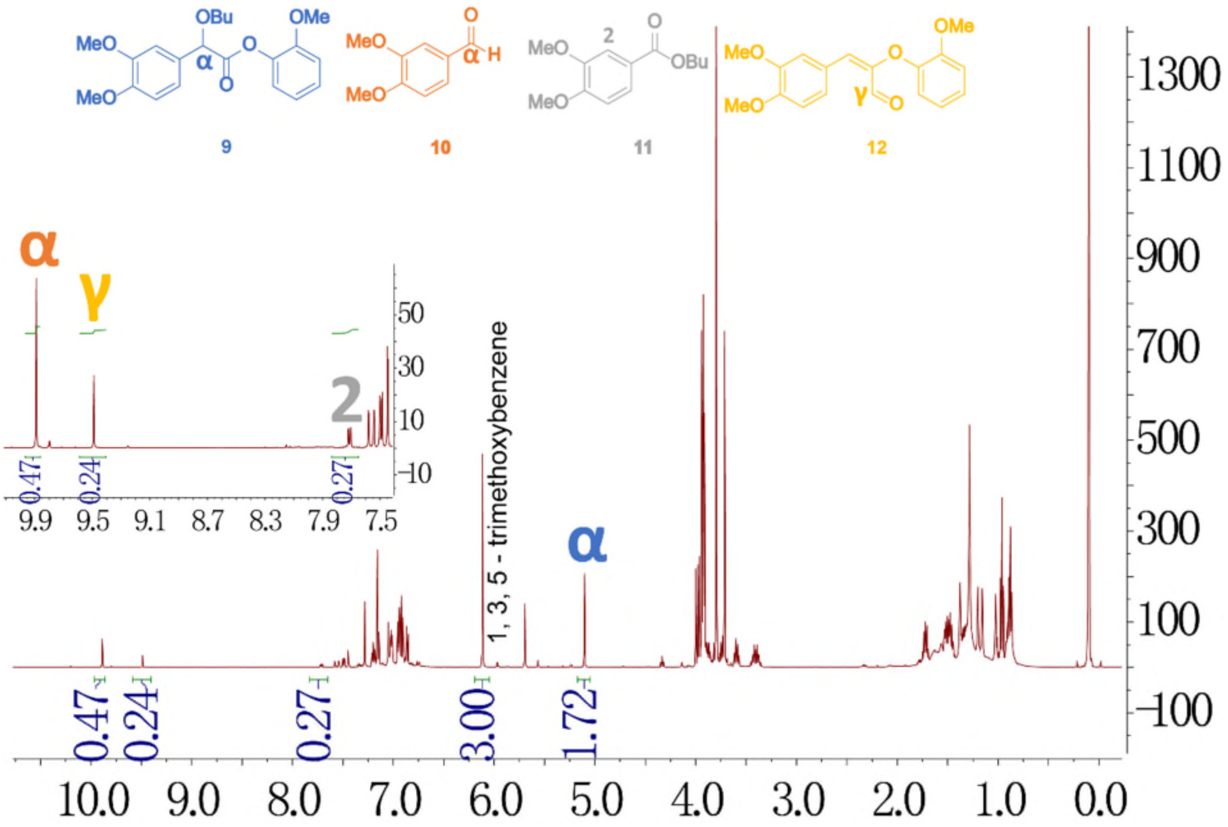

Figure S26: Quantitative ^{1}H NMR spectrum ($CDCl_3$) for the oxidation of **8:** TEMPO (1.5 eq.), CuCl (0.1 eq.) under Ar in pyridine for 9 hours. (**Figure 2**, **entry 1** in manuscript and **Figure S24**, **entry 1**)

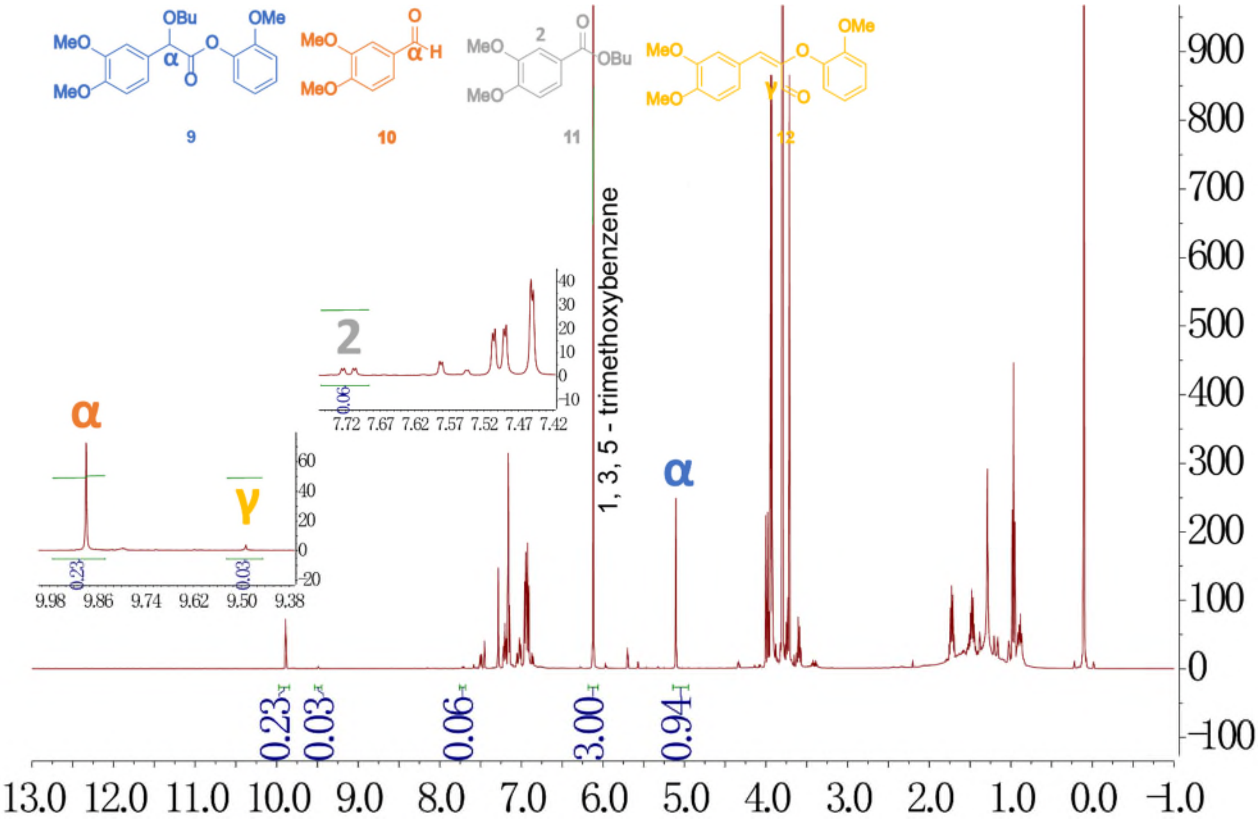

Figure S27: Quantitative ^{1}H NMR spectrum ($CDCl_3$) for the oxidation of **8:** TEMPO (2.0 eq.), CuCl (0.1 eq.) under Ar in pyridine for 9 hours. (**Figure 2**, **entry 2** in manuscript and **Figure S24**, **entry 2**)

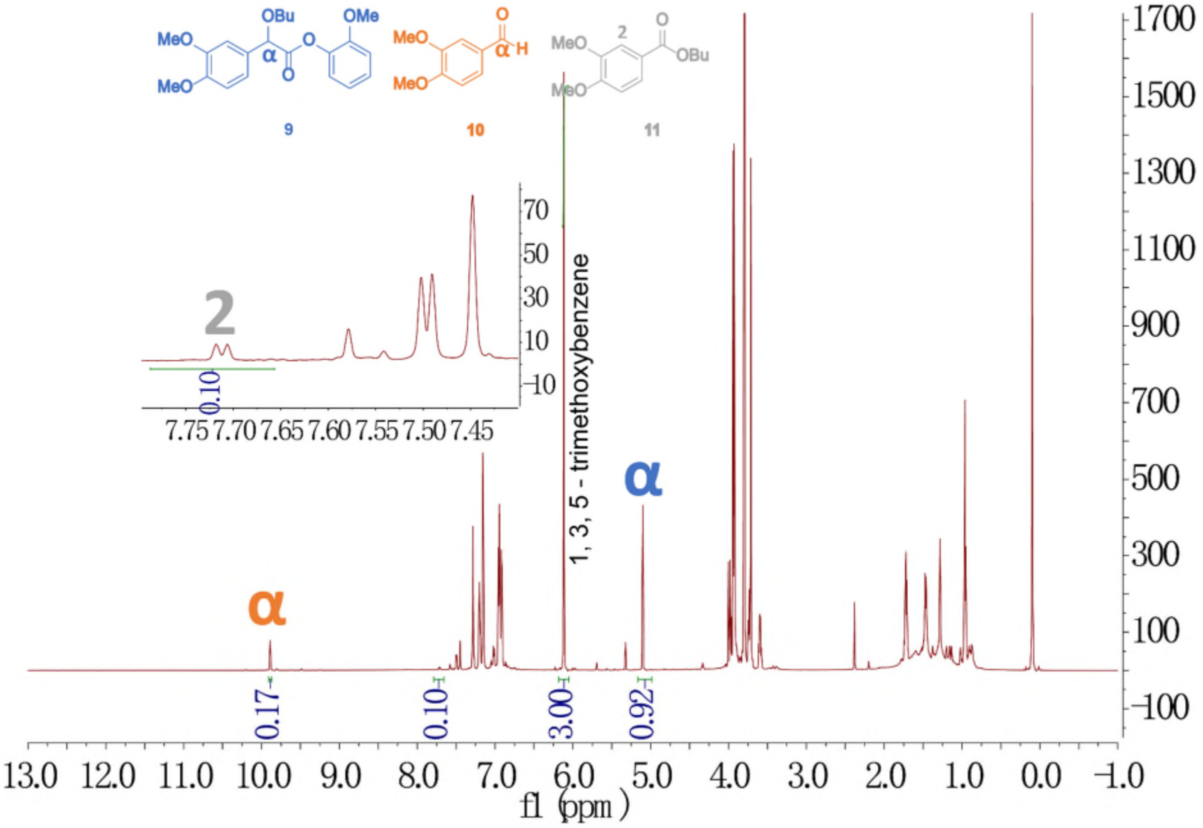

Figure S28: Quantitative ^{1}H NMR spectrum ($CDCl_3$) for the oxidation of **8:** TEMPO (3.0 eq.), CuCl (0.1 eq.) under Ar in pyridine for 9 hours. (**Figure 2**, **entry 3** in manuscript and **Figure S24**, **entry 3**)

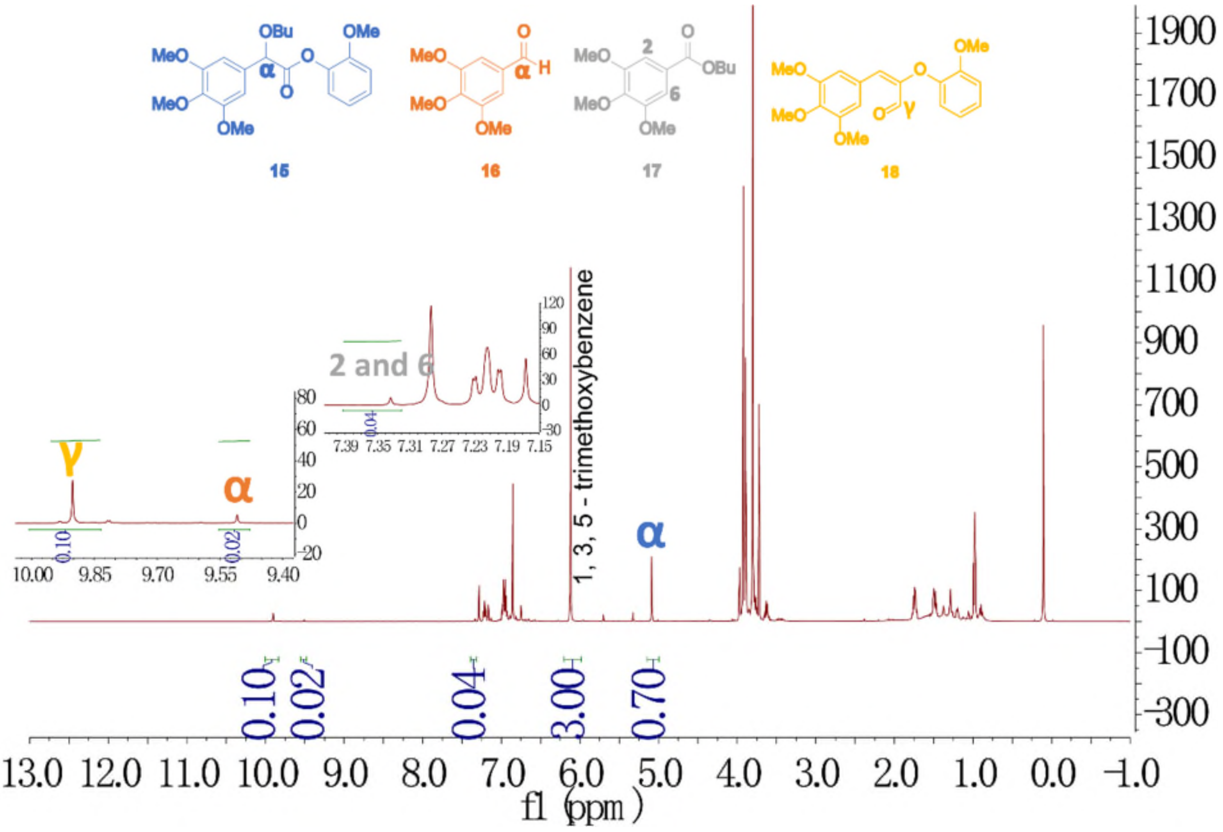

Figure S29: Quantitative ^{1}H NMR spectrum ($CDCl_3$) for the oxidation of **13:** TEMPO (3.0 eq.), CuCl (0.1 eq.) under Ar in pyridine for 9 hours. (**Figure 2**, **entry 4** in manuscript and **Figure S24**, **entry 4**)

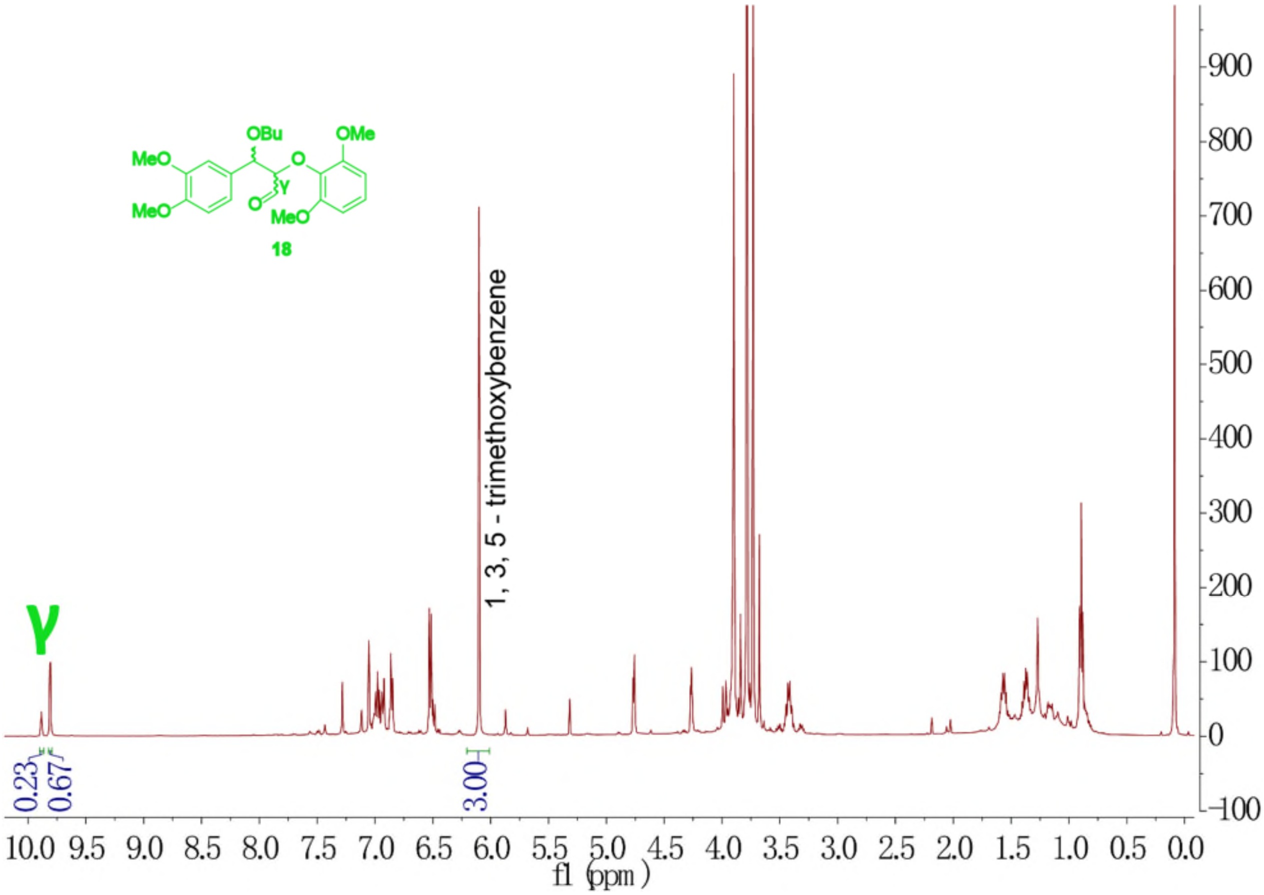

Figure S30: Quantitative ^{1}H NMR spectrum ($CDCl_3$) for the oxidation of **18:** TEMPO (3.0 eq.), CuCl (0.1 eq.) under Ar in pyridine for 20 hours. (**Figure 2**, **entry 5** in manuscript)

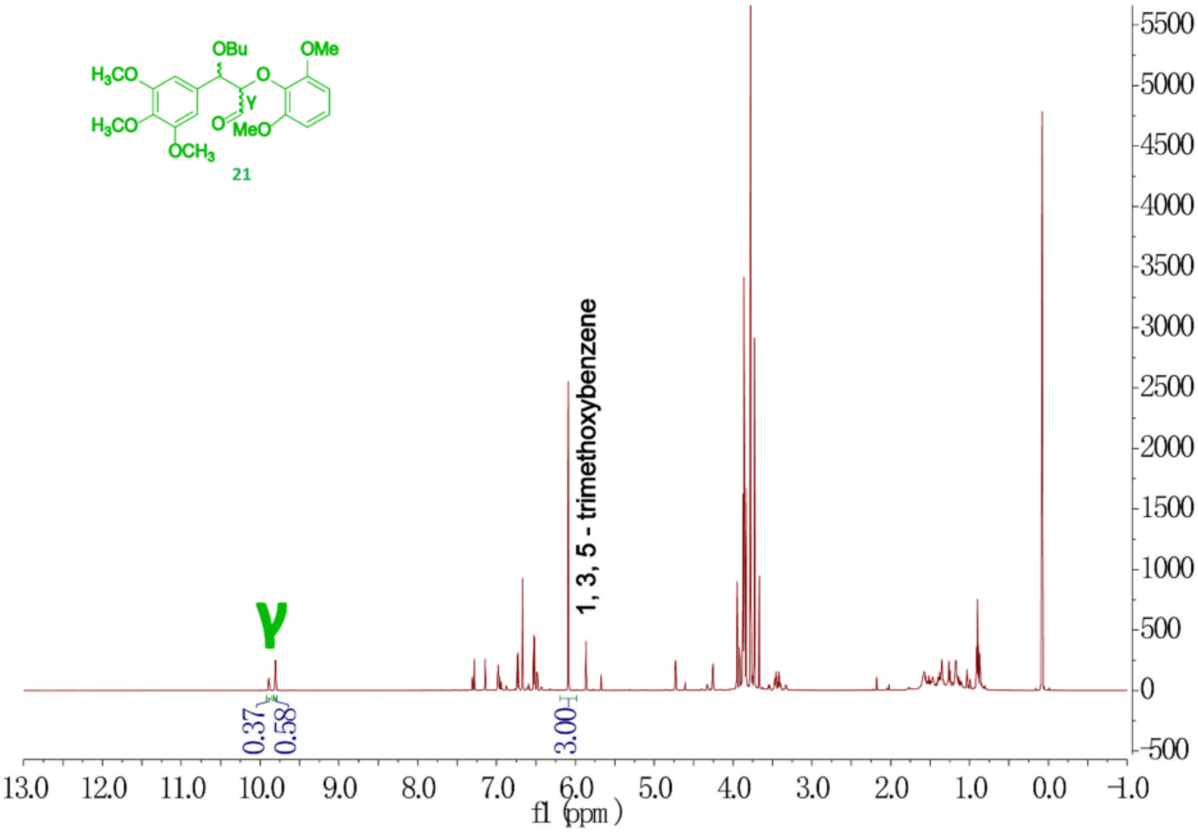

Figure S31: Quantitative ^{1}H NMR spectrum ($CDCl_3$) for the oxidation of **21**: TEMPO (3.0 eq.), CuCl (0.1 eq.) under Ar in pyridine for 20 hours. (**Figure 2**, **entry 6** in manuscript)

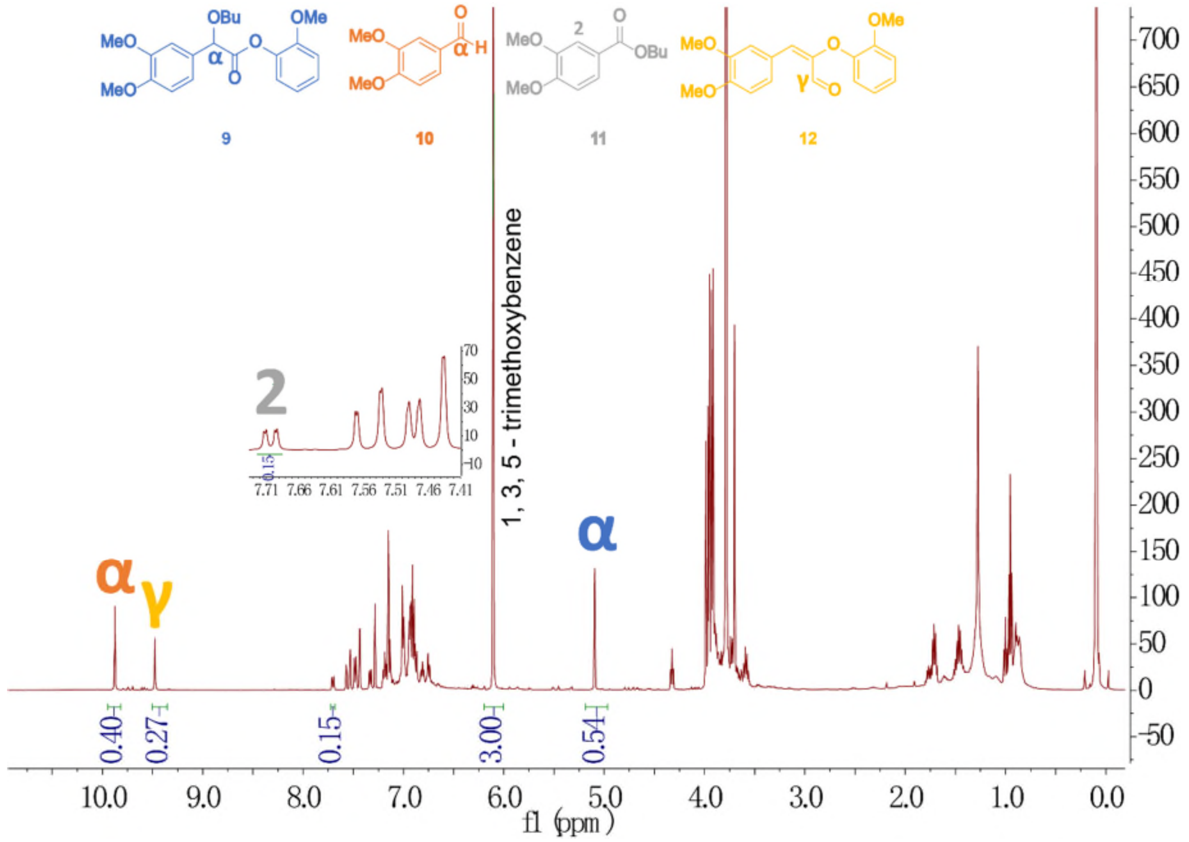

Figure S32: Quantitative ^{1}H NMR spectrum ($CDCl_3$) for negative control reaction for **8**: CuCl (0.1 eq.) under Ar in normal pyridine for 9 hours. (**Figure S25**, **entry 1**)

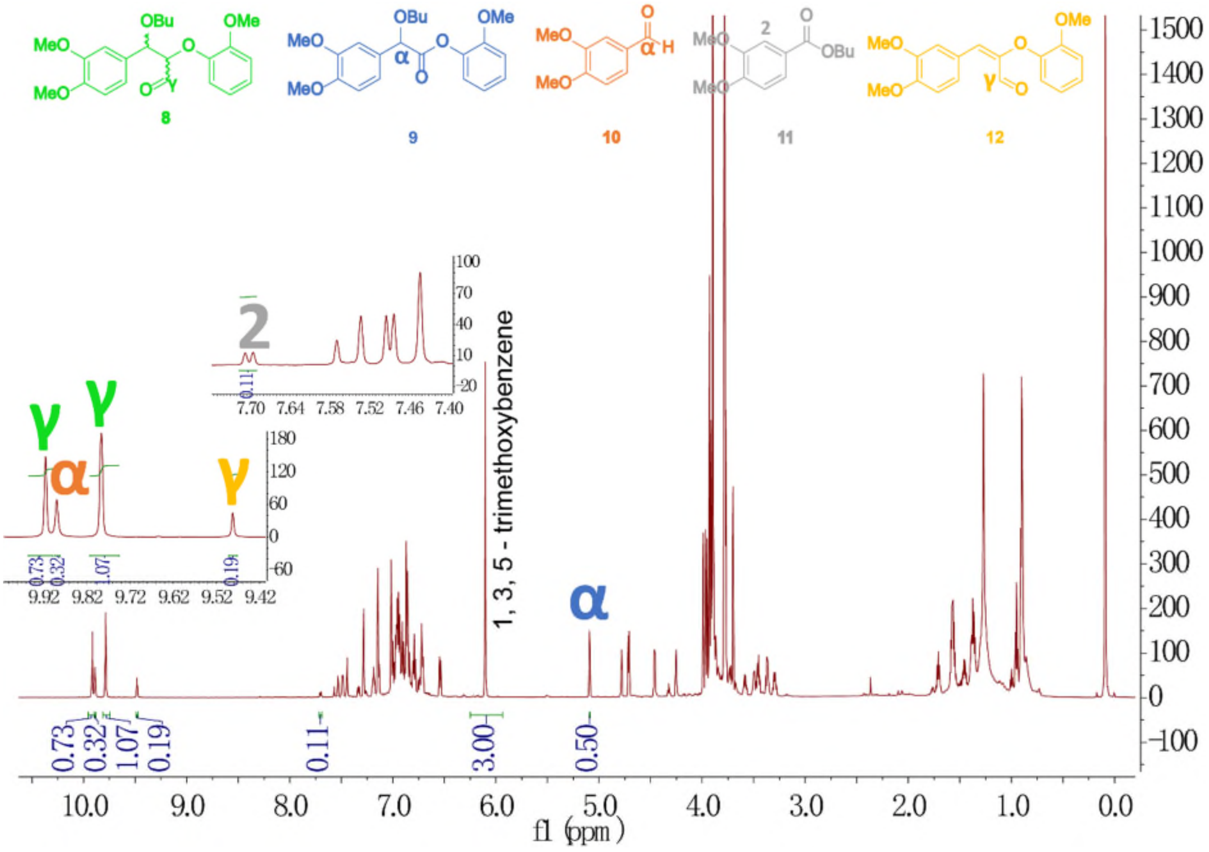

Figure S33: Quantitative ^{1}H NMR spectrum ($CDCl_3$) for negative control reaction for **8**: CuCl (0.1 eq.) under Ar in pyridine (freshly distilled and degassed) for 9 hours. (**Figure S25**, **entry 2**)

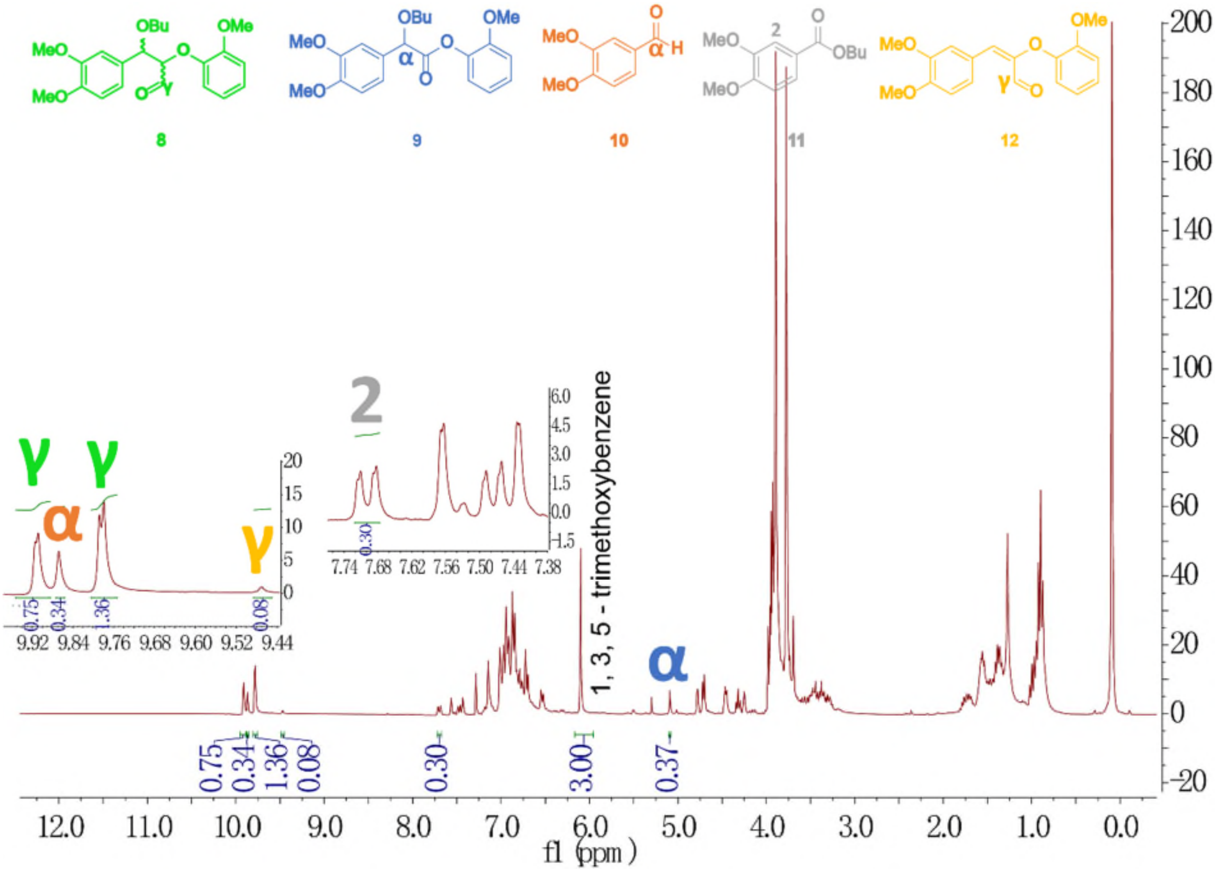

Figure S34: Quantitative ^{1}H NMR spectrum ($CDCl_3$) for negative control reaction for **8**: CuCl (0.1 eq.) under Ar in pyridine (freshly distilled and degassed) for 9 hours. (**Figure S25**, **entry 2**)

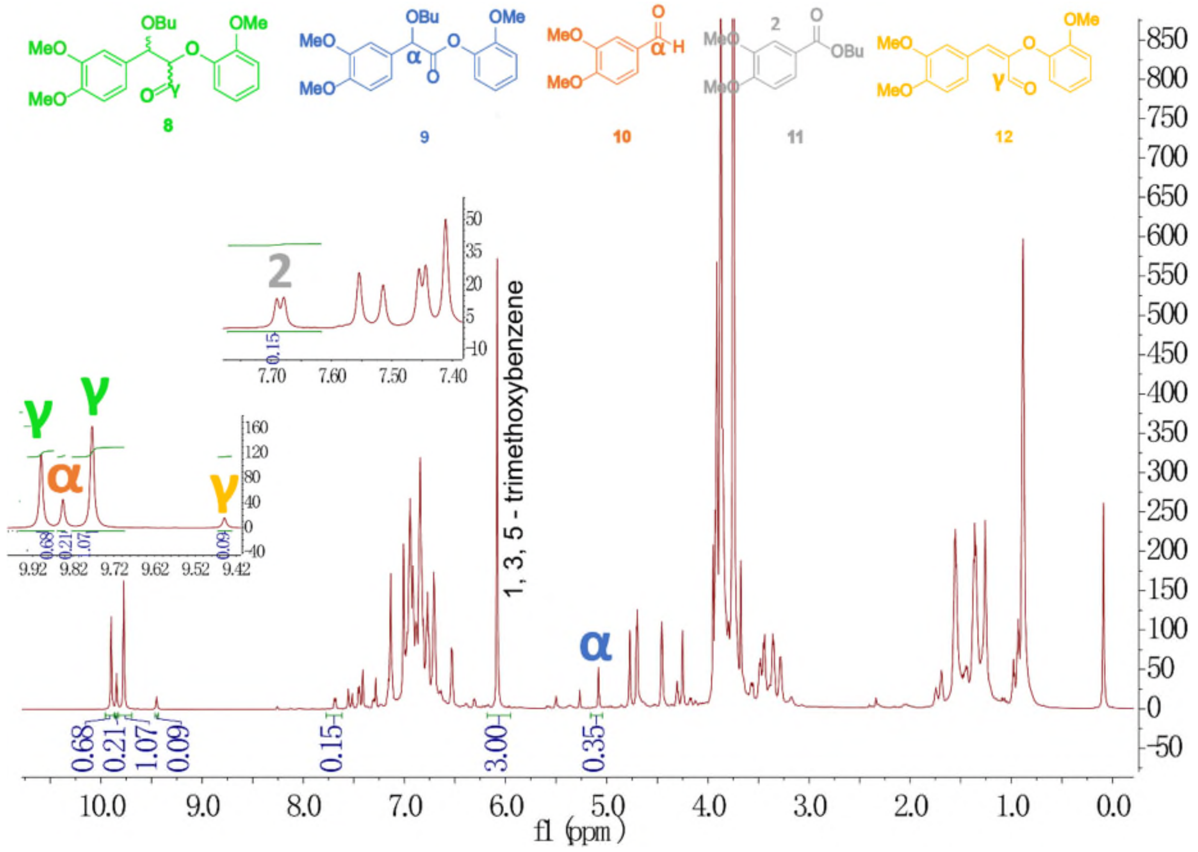

Figure S35: Quantitative ^{1}H NMR spectrum ($CDCl_3$) for negative control reaction for **8**: CuCl (0.1 eq.) under Ar in pyridine (freshly distilled and degassed) for 9 hours. (**Figure S25**, **entry 2**)

7.4. Reactions followed by isolating of TEMPO-adduct 22

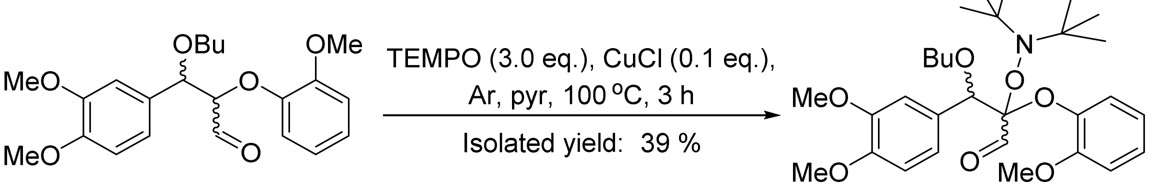

8 **22**

To a 25 mL flame dried Schlenk flask under an argon atmosphere was added CuCl (4 mg, 0.039 mmol, 10 mol%). The flask was then sealed with a septum and connected to the high vacuum for 5 min to remove any remaining air, after which the flask was filled with Ar using an Ar balloon. The lignin model compound **8** (150mg, 0.39 mmol, 1.0 eq.) and TEMPO (182 mg, 1.17 mmol, 3.0 eq.) in pyridine (4 mL) were added via syringe [Caution: pyridine should be freshly distilled and degassed]. The reaction was heated at 100 °C for 3 hours. After cooling to room temperature, the solvent was then removed *in vacuo*. The residue was subjected to column chromatography (10% EtOAc/Hex) to produce **22** (81.5 mg, 0.15 mmol, 39%) as a colourless oil.

3-butoxy-3-(3,4-dimethoxyphenyl)-2-(2-methoxyphenoxy)-2-((2,2,6,6-tetramethylpiperidin-1-yl)oxy) propanal (22): **HRMS** (ESI) calculated for $C_{31}H_{45}NO_7Na$ 566.3094, $[M + Na]^+$, 566.3070. **IR** (thin film): 3524, 2930, 2862, 1741, 1591, 1499, 1456, 1364, 1252, 1234, 1050, 843, 789 cm^{-1}. *Major:* ^{1}**H NMR** (500 MHz, $CDCl_3$) δ 9.95 (s, 1H, Hγ), 7.36 (d, J = 8.1 Hz, 1H, H12), 6.89 (dd, J = 7.9, 7.9 Hz, 1H, H10), 6.76 (dd, J = 7.9, 7.9 Hz, 1H, H11), 6.75 (d, J = 8.3 Hz, 6.8 Hz, 1H, H9), 6.72 – 6.64 (m, 2H, H5 and H6), 6.67 (s, 1H, H2), 5.21 (s, 1H, Hα), 3.72 (s, 3H, OC**H₃**), 3.59 (s, 3H, OC**H₃**), 3.57 (s, 3H, OC**H₃**), 3.32 – 3.20 (m, 2H, OC**H₂**CH₂), 1.64 – 1.27 (m, 10H, OCH₂C**H₂**, C**H₂**CH₃, 2 × H14, 2 × H15, 2 × H16), 1.28 – 1.04 (m, 12H, 4 × C**H₃**), 0.84 (t, J = 7.4 Hz, 3H, CH₂C**H₃**). ^{13}**C NMR** (126 MHz, $CDCl_3$) δ 193.6 (Cγ), 150.3 (C8), 148.3 (C3), 147.9 (C4), 144.3 (C7), 129.5 (C1), 122.3 (C10), 121.6 (C6), 120.9 (C12), 120.2 (C11), 113.3 (C9), 111.4 (C2), 110.0 (C5), 105.5 (Cβ), 81.5 (Cα), 69.5 (O**C**H₂CH₂), 62.4 (C13 or C17), 62.2 (C13 or C17), 56.2 (O**C**H₃), 55.8 (O**C**H₃), 55.4 (O**C**H₃), 41.2 (C14 and C16), 31.6 (OCH₂**C**H₂), 21.2 (4 × **C**H₃), 19.2 (**C**H₂CH₃), 16.9 (C15), 13.9 (CH₂**C**H₃). *Minor:* ^{1}**H NMR** (500 MHz, $CDCl_3$) δ 9.67 (s, 1H, Hγ), 7.82 (dd, J = 8.1 Hz, 1.6 Hz, 1H, H12), 6.96 (td, J = 7.7 Hz, 1.6 Hz, 1H, H11), 6.89 (dd, J = 7.9, 7.9 Hz, 1H, H10), 6.82 (dd, J = 8.0 Hz, 1.6 Hz, 1H, H9), 6.72 – 6.64 (m, 2H, H5 and H6), 6.67 (s, 1H, H2), 5.21 (s, 1H, Hα), 3.85 (s, 3H, OC**H₃**), 3.84 (s, 3H, OC**H₃**), 3.71 (s, 3H, OC**H₃**), 3.20 – 3.15 (m, 2H, OC**H₂**CH₂), 1.64 – 1.27 (m, 10H, OCH₂C**H₂**, C**H₂**CH₃, 2 × H14, 2 × H15, 2 × H16), 1.28 – 1.04 (m, 12H, 4 × CH₃), 0.88 (t, J = 7.4 Hz, 3H, CH₂C**H₃**). ^{13}**C NMR** (126 MHz, $CDCl_3$) δ 194.8 (Cγ), 150.6 (C8), 148.2 (C3), 147.9 (C4), 144.4 (C7), 129.3 (C1), 122.3 (C10), 120.9 (C6), 120.2 (C11), 119.4 (C12), 113.0 (C9), 112.3 (C2), 109.7 (C5), 105.3 (Cβ), 83.6 (Cα), 68.8 (O**C**H₂CH₂), 62.2 (C13 or C17), 61.6 (C13 or C17), 56.2 (O**C**H₃), 55.8 (O**C**H₃), 55.3 (O**C**H₃), 41.5 (C14 and C16), 32.1 (OCH₂**C**H₂), 21.4 (4 × **C**H₃), 19.5 (**C**H₂CH₃), 16.9 (C15), 14.0 (CH₂**C**H₃).

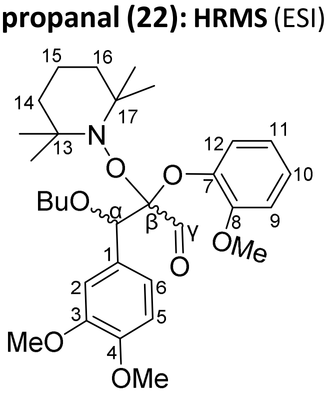

7.5. Time course

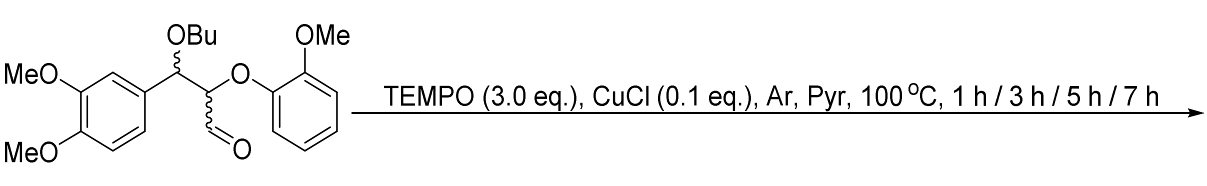

8

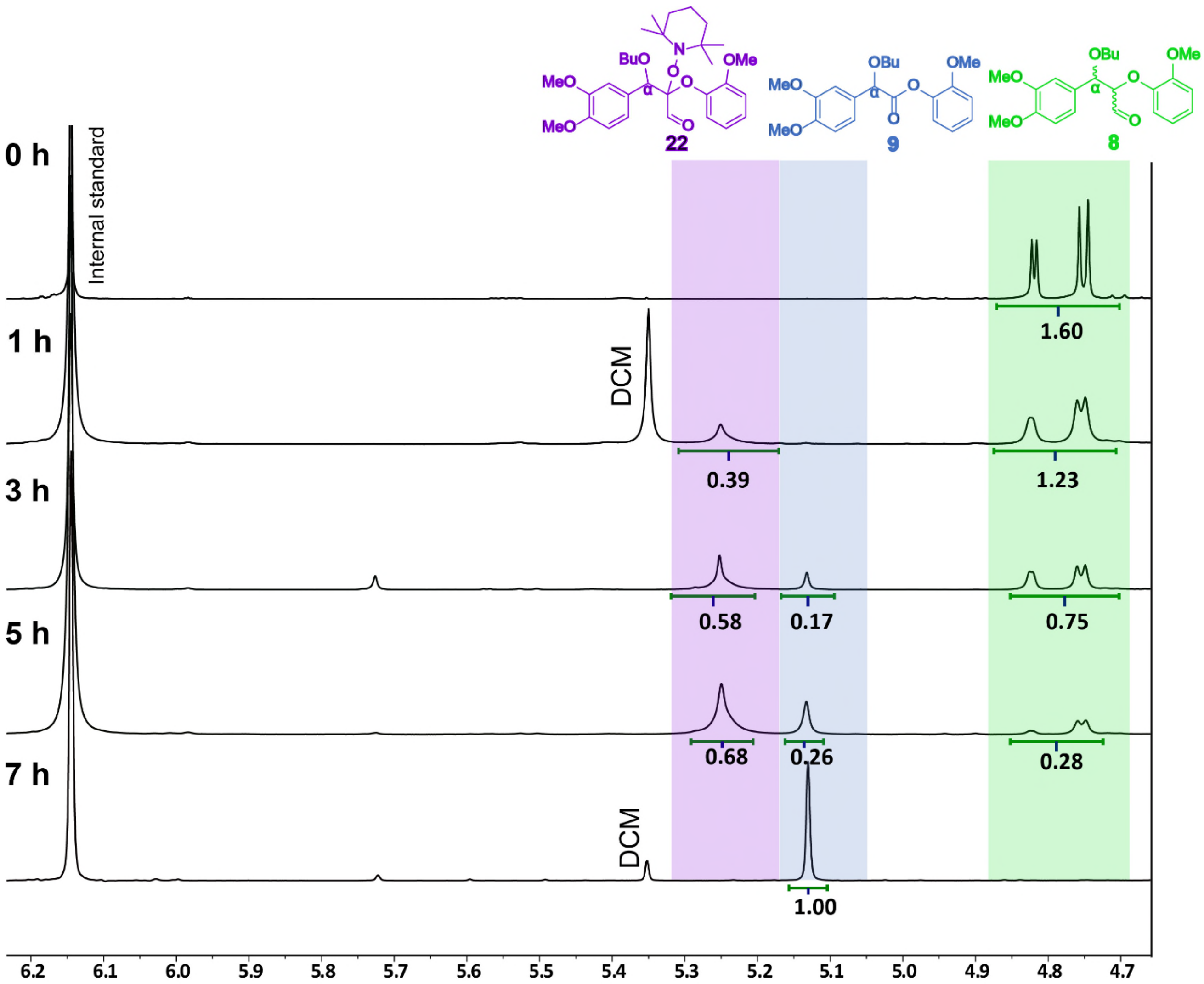

Figure S36: Quantitative ^{1}H NMR analysis of the crude mixture shown above at the stated time points. Peak at 6.10 ppm corresponds to the signal for the internal standard 1,3,5-trimethoxybenzene; peak at 5.21 ppm corresponds to the α proton for TEMPO-adduct **22**; peak at 5.10 ppm corresponds to the α-proton for aryl ester **9** and peak at 4.81 – 4.70 ppm corresponds to α-proton for each diastereomer of the starting material **8**. As reaction time increased, the starting material **8** was consumed, the TEMPO-adduct **22** appeared and then disappeared, and the yield of final ester product **9** increased gradually. Reactions were conducted on a 0.1 mmol scale following the general procedure **7.1**. Yields were determined by ^{1}H NMR by using 1,3,5-trimethoxybenzene as the internal standard. Full spectra are shown in **Figures S37-S40.**

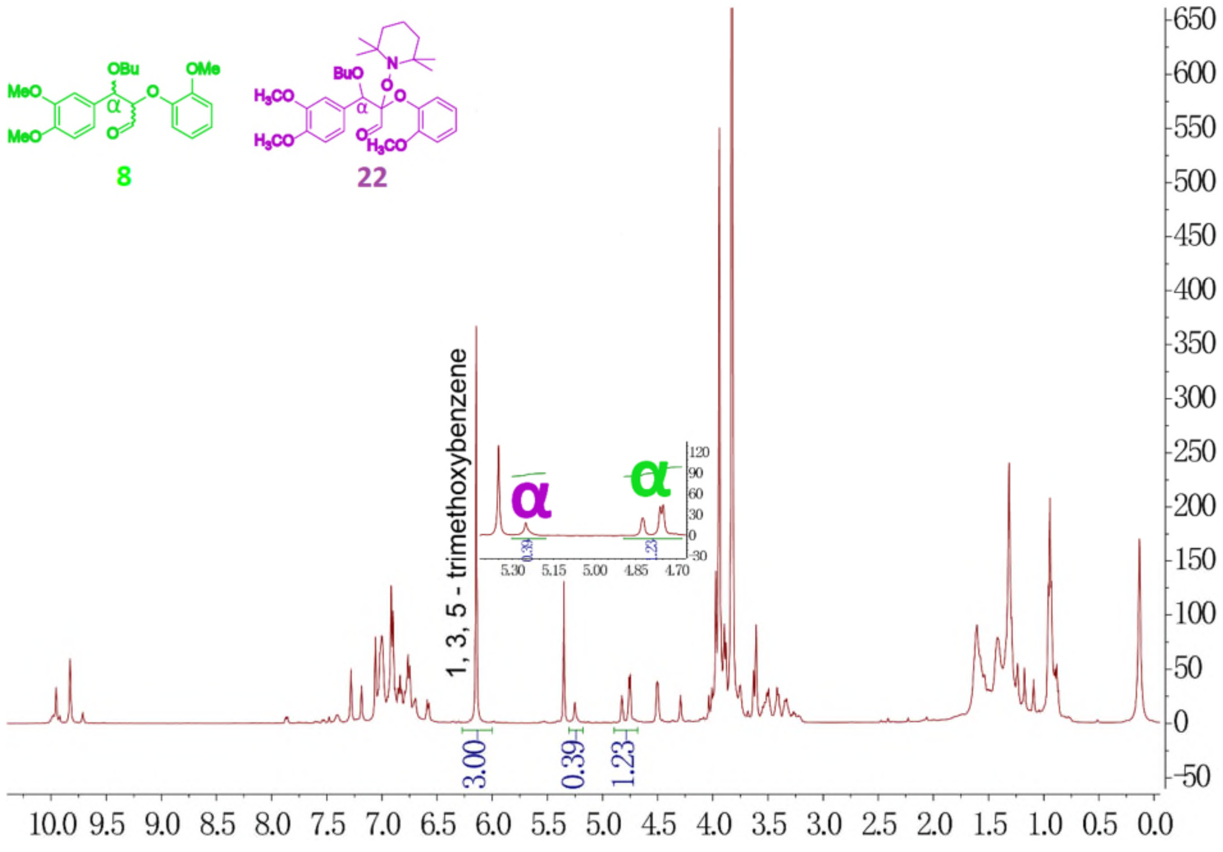

Figure S37: Quantitative ^{1}H NMR spectrum ($CDCl_3$) for time course: **8** with TEMPO (3.0 eq.), CuCl (0.1 eq.) under Ar in pyridine for 1 hours. (**Scheme 3B** in manuscript, **Figure S36**)

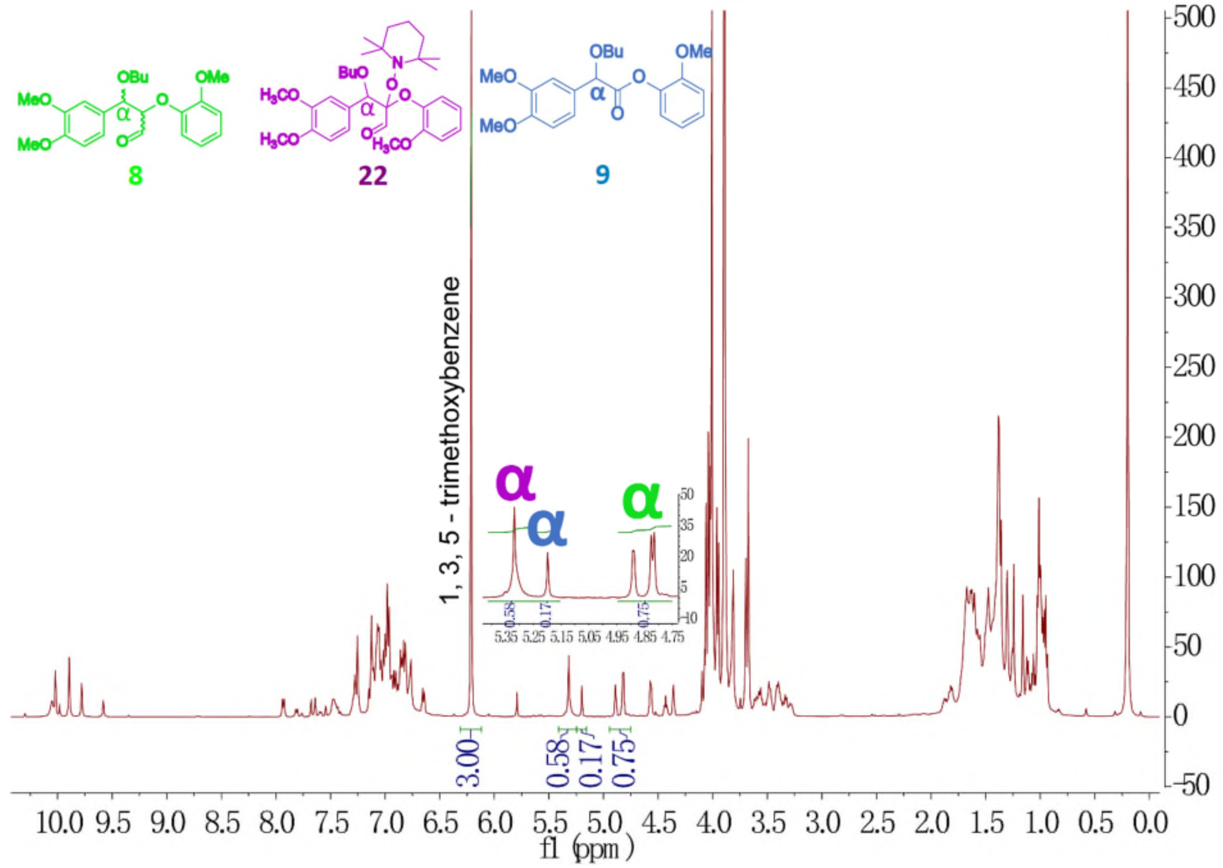

Figure S38: Quantitative ^{1}H NMR spectrum ($CDCl_3$) for time course: **8** with TEMPO (3.0 eq.), CuCl (0.1 eq.) under Ar in pyridine for 3 hours. (**Scheme 3B** in manuscript, **Figure S36**)

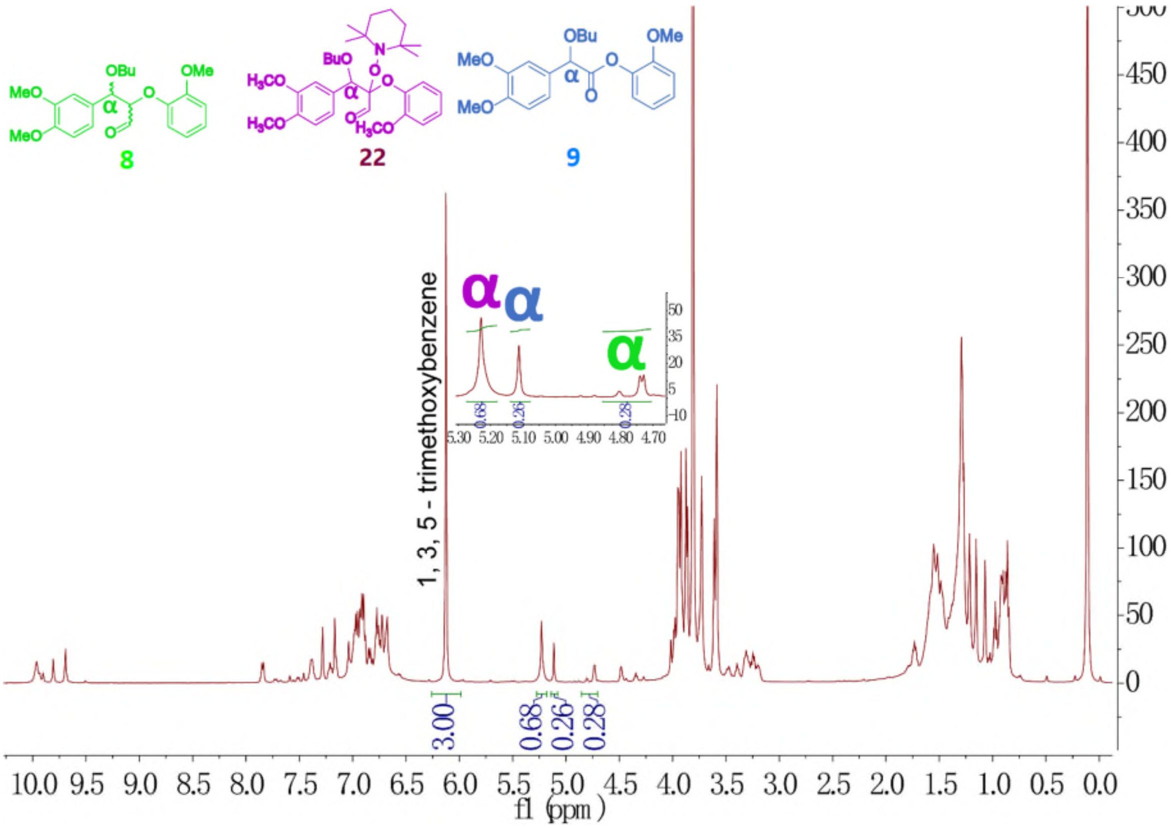

Figure S39: Quantitative ^{1}H NMR spectrum ($CDCl_3$) for time course: **8** with TEMPO (3.0 eq.), CuCl (0.1 eq.) under Ar in pyridine for 5 hours. (**Scheme 3B** in manuscript, **Figure S36**)

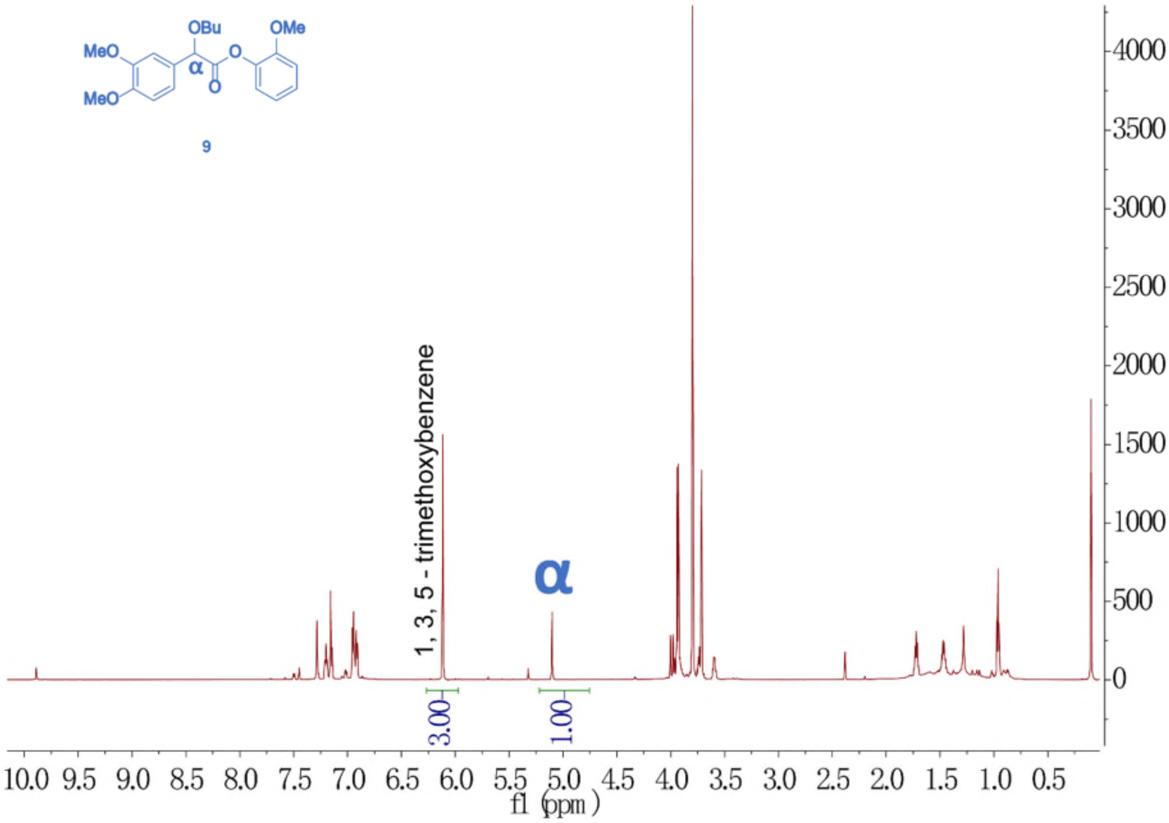

Figure S40: Quantitative ^{1}H NMR spectrum ($CDCl_3$) for time course: **8** with TEMPO (3.0 eq.), CuCl (0.1 eq.) under Ar in pyridine for 7 hours. (**Scheme 3B** in manuscript, **Figure S36**)

sasas8. Hydrolysis of aryl ester 9

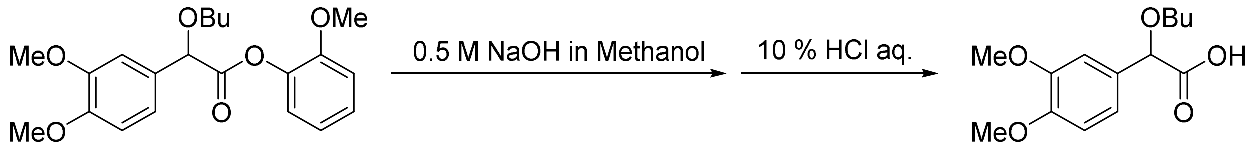

9 **S7**

The ester **9** (100 mg, 0.27 mmol) was dissolved in a solution of NaOH in methanol (0.5 M, 2 mL) and stirred for 3 hours at room temperature. The mixture was acidified with 10 % aqueous HCl (5 mL) and then extracted with ethyl acetate (3 x 5 mL). The organic extracts were combined, washed with brine (1 x 5 mL), dried (Na_2SO_4) and concentrated *in vacuo*. The acid product **S7** was obtained as a light-yellow oil in quantitative yield.

2-Butoxy-2-(3,4-dimethoxyphenyl) acetic acid (S7):

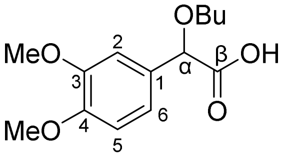

HRMS (ESI) calculated for $C_{14}H_{19}O_5$ 267.1232, $[M-H]^-$, found 267.1237. **IR** (thin film): 2962, 1707, 1421, 1210, 893, 817, 760 cm^{-1}. **^{1}H NMR:** ^{1}H NMR (500 MHz, $CDCl_3$) δ 9.31 (s, 1H, OH), 7.02 – 6.94 (m, 2H, H2, H6), 6.84 (d, J = 8.1 Hz, 1H, H5), 4.81 (s, 1H, Hα), 3.87 (s, 3H, OCH_3), 3.87 (s, 3H, OCH_3), 3.52 (dt, J = 9.0, 6.5 Hz, 1H, 1 x O**CH₂**CH₂), 3.49 – 3.41 (m, 1H, 1 x O**CH₂**CH₂), 1.63 (q, J = 7.1 Hz, 2H, OCH₂**CH₂**), 1.43 – 1.34 (m, 2H, **CH₂**CH₃), 0.93 – 0.83 (m, 3H, CH₂**CH₃**); **^{13}C NMR** (126 MHz, $CDCl_3$) δ 175.7 (Cβ), 149.2 (C3), 149.2 (C4), 128.4 (C1), 120.1 (C6), 110.9 (C5), 109.8 (C2) , 80.3 (Cα), 69.5 (O**C**H₂CH₂), 55.9 (O**C**H₃), 55.9 (O**C**H₃), 31.5 (OCH₂**C**H₂), 19.2 (**C**H₂CH₃), 13.8 (CH₂**C**H₃).

9. Synthesis of all-G β-O-4 γ-aldehyde model oligomers S9, 24-26

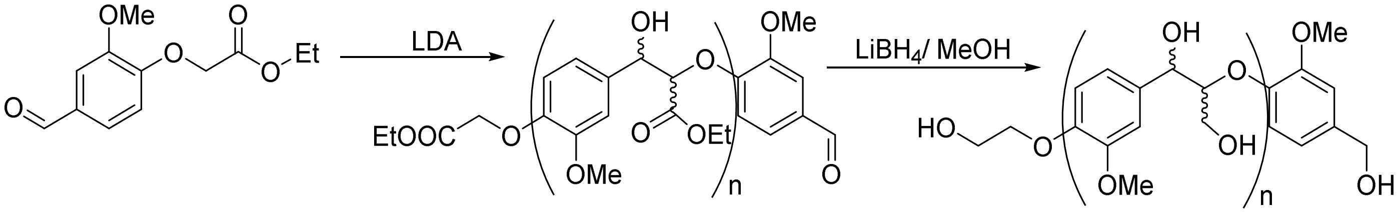

S8 All-G polymerized γ-ester **S9** All-G β-O-4 polymer **24**

9.1. Aldol polymerization

This was carried out as shown above in accordance with literature procedures[S11], all G polymer **S9** was prepared by using monomer **S8**.

9.1.1. Oligoester S9 analysis

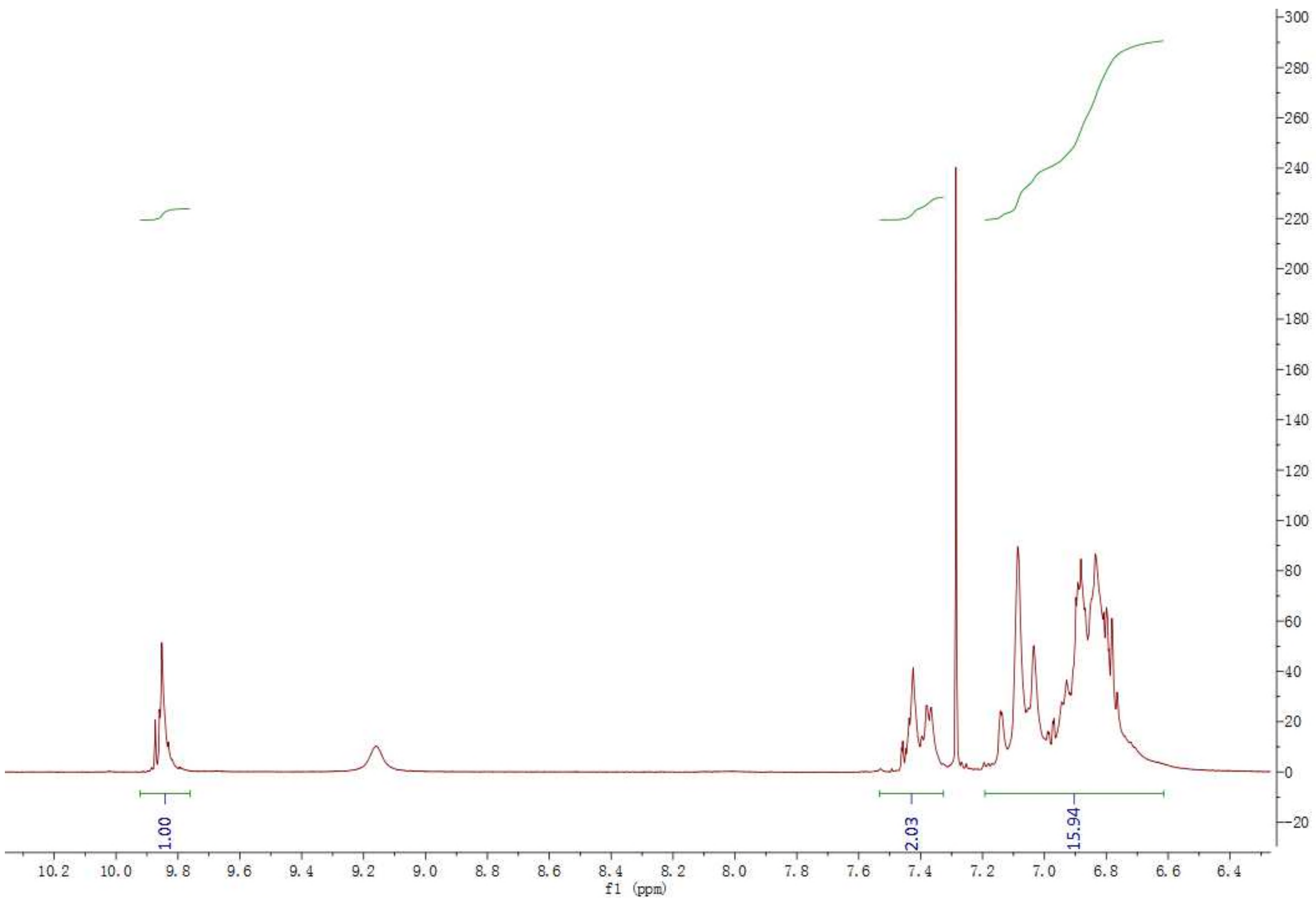

Figure S41: Quantitative ^{1}H NMR (700 MHz, $CDCl_3$) of all G oligoester **S9** after aldol condensation.

The degree of polymerization after the aldol reaction was calculated based on a literature method[S11]:
The integral of the aldehyde region (end group): 1
The integrals of all aromatic region: 2.03 + 15.94 = 17.97
Calculation is as below:
DP_n = (17.97/3)/1 = 5.9 (the result is similar to the value in the literature[S11])
There are 3 protons per aromatic ring therefore the sum of aromatic integrals is divided by 3.

9.2. The reduction of S9 to form native β-O-4 all-G oligomer 24

9.2.1. General procedure for the reduction

The native β-O-4 oligomer **24** was prepared according to literature precedent.[S11-13] It should be noted that in previous studies complete reduction of the ester groups in a related β-O-4 S-G oligomer was achieved. This was not found to be the case during this attempt to prepare **24**. The crude product **S9** (16 g, 65 mmol, 1.0 eq.) from the previous step was dissolved in THF (400 mL). To this solution was added lithium borohydride (7.0 g, 325 mmol, 5.0 eq.) and the reaction mixture was heated to 60 °C. Methanol (13 mL, 325 mmol, 5.0 eq) was then added dropwise over 15 mins and the reaction was heated at 60 °C overnight. The reaction mixture was then concentrated and water (400 mL) was added to the residue. The crude oligomer was precipitated by acidification with conc. HCl. The product was collected as a light-yellow gum and dried *in vacuo*. The crude oligomer was then taken up in acetone/methanol (9:1, 20 mL), filtered and precipitated by dropwise addition into diethyl ether (300 mL). The oligomer **24** was collected by filtration and dried *in vacuo* to give a beige powder (~2.5 g, ~15 %). A full 2D HSQC spectra is shown in **Figure S42**.

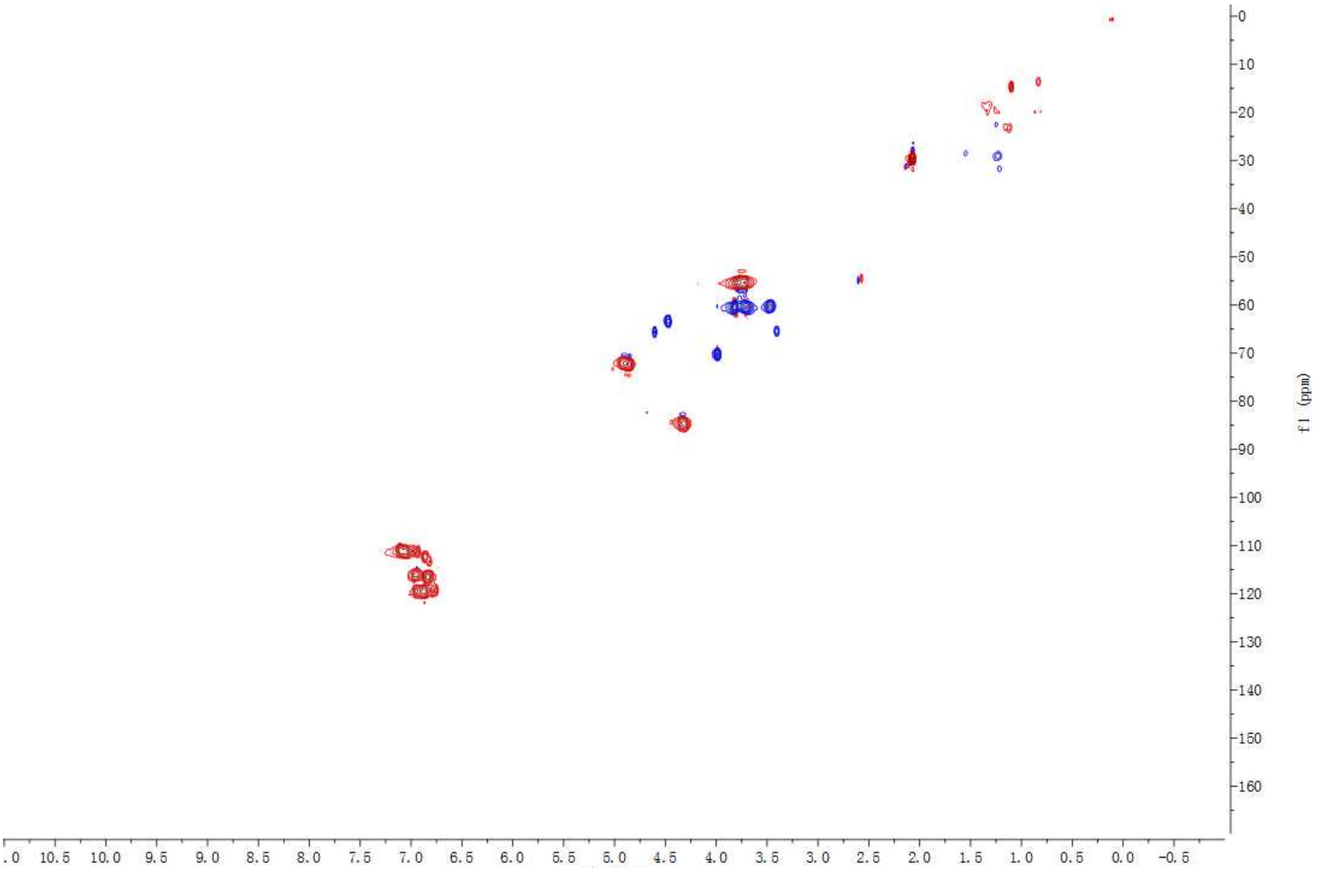

Figure S42: Full 2D HSQC NMR of native all-G β-O-4 oligomer **24.**

9.2.2. The determination of DP_n of 24 using 2D HSQC NMR analysis after reduction

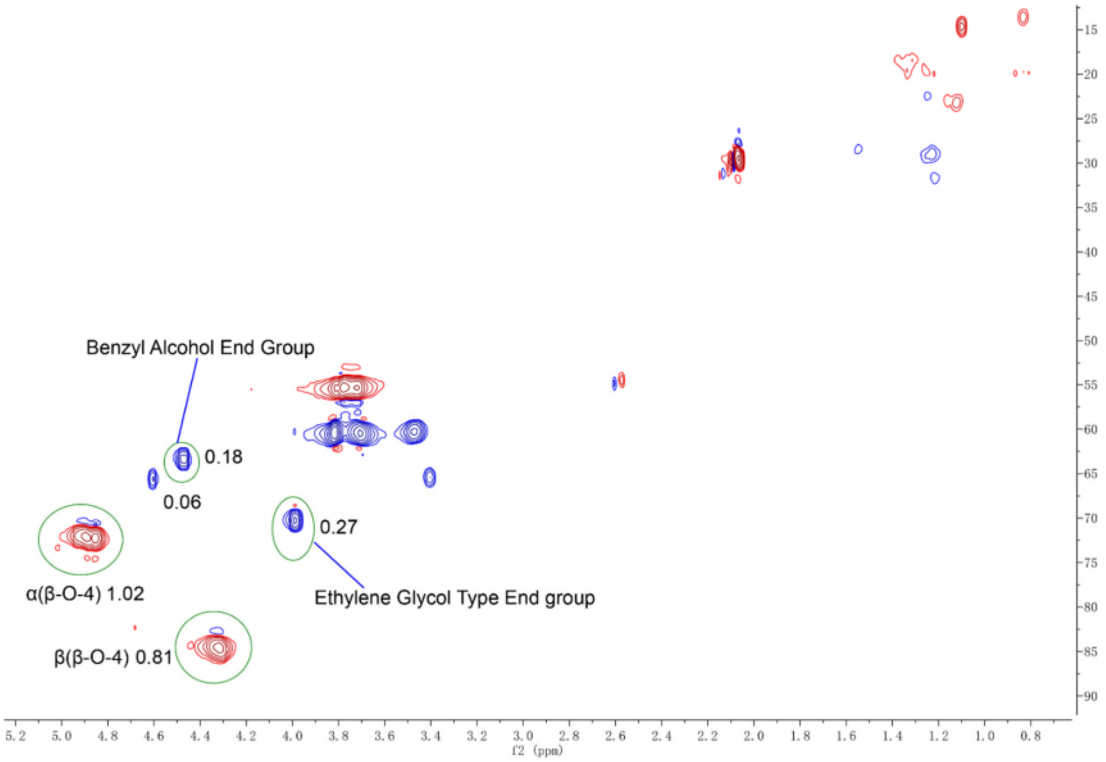

Figure S43: A part (β-O-4 linkage region and aliphatic region) of the 2D HSQC spectra of **24** with integration values that were used in the calculation of the DP_n of **24**. (Full spectrum is shown in **Figure S42**)

The degree of polymerization after reduction was calculated based on literature method[13]:

Calculation is as below: $DP_n = [(0.81+1.02)/2] / [(0.18 + 0.27)/4] = 8.13$

Two end groups are present per oligomer chain and each one is a CH_2 group therefore the sum of the end group integrals is divided by 4.

The calculated value of DP_n after reduction (8.1) is higher than that after the aldol condensation reaction (5.9). One possible explanation is that the lower molecular weight oligoester chains in **S9** when reduced to the lower molecular weight oligomer chains in **24**, were soluble in the aqueous solution after acidic workup and failed to precipitate. In other words, the fraction of **24** that precipitated contained only relatively large oligomer chains.

9.2.3. Discussion about the presence of non-reducible ester units in 24

Even though the yield of the reduction step in the dimer study was quantitative[S12], the reduction to form **24** from oligoester **S9** in this study could not be driven to completion. This was identified based on the 2D HSQC analysis of **24** (**Figure S44**). The presence of ester units in **24** was confirmed by overlapping the 2D HSQC spectrum of **24** with that of the known compound **S10**[S12].

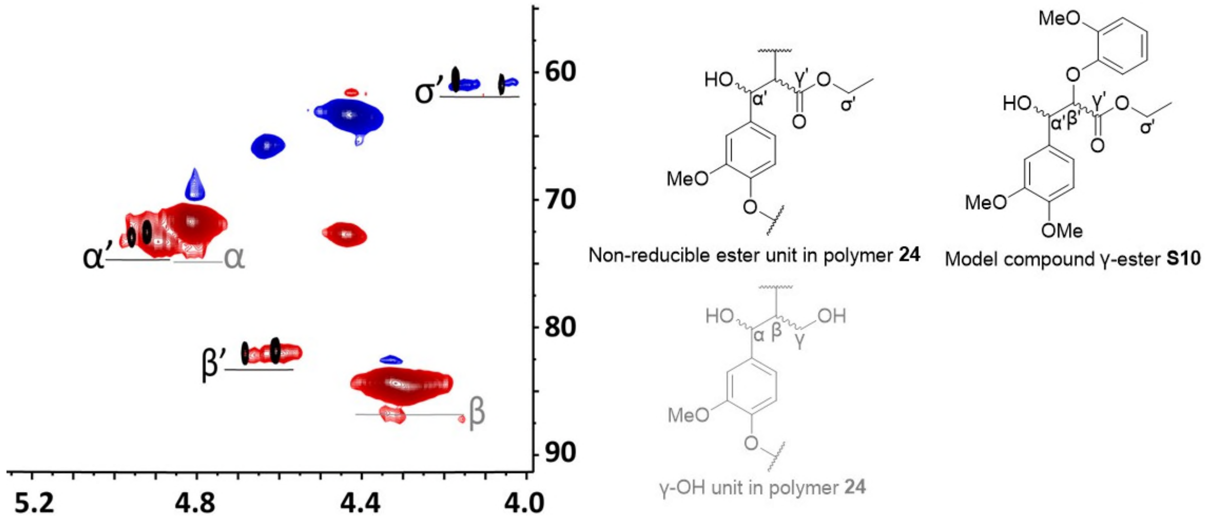

Figure S44: A part (β-O-4 linkage) of the 2D HSQC analysis of **24**, showing the presence of the remaining ester unit in **24**. (Full spectrum shown in **Figure S42**)

Efforts were made to reduce all the ester units in **24** but to date have been unsuccessful in this study. A stronger reducing system of $LiBH_4$- MeOH-Et_2O[S14] was applied instead of the reducing system of the $NaBH_4$-MeOH-EtOH in the literature[S11-13]. There were some improvements, but full reduction was not achieved (data not shown). One possible explanation is that some certain positions in the oligomer chain are sterically inaccessible to the alkoxyhydride formed from $LiBH_4$ or $NaBH_4$.

9.3. Butanosolv pretreatment of oligomer 24

9.3.1. General procedure for butanosolv reaction

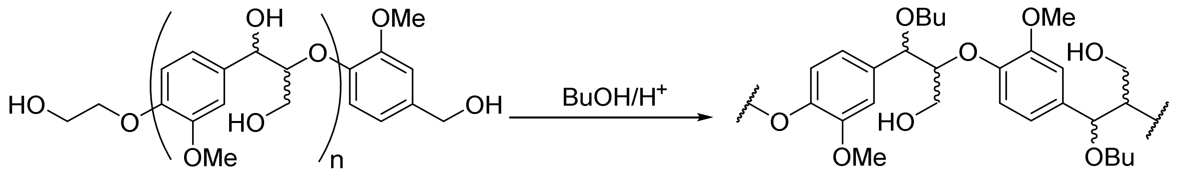

All-G β-O-4 polymer **24**

All-G butanated β-O-4 polymer **25**

To a mixture of **24** (1.37 g, 6.5 mmol) in n-butanol (100 mL) was added 12 N hydrochloric acid (3 mL). The reaction mixture was then heated at reflux for 1.5 h. The mixture was quenched with sat. aqueous $NaHCO_3$ (5 mL) and extracted with EtOAc (3 x 15 mL). The combined organic layers were washed with sat. aqueous NaCl (15 mL), dried with Na_2SO_4 and concentrated *in vacuo* to give **25** (1.75 g, ~100 %) as a brown powder. Full 2D HSQC analysis is shown in **Figure S46.**

9.3.2. The assignment of non-reducible ester unit and γ-OH in oligomer 25.

To assign any residual non-reduced ester units in oligomer **25,** the model compound **S11** (**Figure S45**) was synthesized as follows: to a mixture of **S10**[S12] (0.15 g, 0.40 mmol, 1.0 eq.) in n-butanol (5 mL) was added 12 M hydrochloric acid (0.5 mL). The reaction mixture was then heated at reflux for 1.5 h. The mixture was quenched with sat. aqueous $NaHCO_3$ (2 mL) and extracted with EtOAc (5 mL). The combined organic layers were washed with sat. aqueous NaCl (5 mL), dried with Na_2SO_4 and concentrated *in vacuo*. Purification was achieved by column chromatography eluting with ethyl acetate: petroleum ether (20 %) to give **S11** as a diastereomeric mixture as a colorless oil (86.4 mg, 0.20 mmol, 50 %, *major*: *minor* = 1.89: 1). **HRMS** (ESI) calculated for $C_{26}H_{36}O_7Na$ 483.2359, $[M + Na]^+$ found 483.2344. **IR** (thin film): 2953, 1748, 1593, 1497, 1125, 1024, 883, 762 cm^{-1}. *Major:* **^{1}H NMR** (400 MHz, $CDCl_3$) δ 7.09 (d, J = 1.9 Hz, 1H), 7.05 (dd, J = 8.2 Hz, 1.9 Hz, 1H), 6.93 (t = 8.2 Hz, 1H), 6.88 – 6.80 (m, 2H), 6.74 (td, J = 7.8 Hz, 1.6 Hz, 1H), 6.63 (dd, J = 7.8 Hz, 1.6 Hz, 1H), 4.68 (d, J = 8.0 Hz, 1H), 4.55 (d, J = 8.0 Hz, 1H), 4.26 – 4.16 (m, 2H), 3.90 (s, 3H), 3.89 (s, 3H), 3.73 (s, 3H), 3.42 – 3.30 (m, 2H), 1.60 – 1.46 (m, 4H), 1.40 – 1.32 (m, 4H), 0.95 – 0.84 (m, 6H). ^{13}C NMR (101 MHz, $CDCl_3$) δ 170.6, 150.4, 148.9, 148.8, 147.4, 131.1, 123.0, 120.8, 120.4, 117.1, 112.1, 110.7, 110.6, 82.7, 81.5, 69.1, 65.0, 55.9, 55.9, 55.8, 31.7, 30.7, 19.3, 19.1, 13.9, 13.7. *Minor*: ^{1}H NMR (400 MHz, $CDCl_3$) δ 7.13 (d, J = 1.9 Hz, 1H), 6.94 (t, J = 8.2 Hz, 1H), 6.89 – 6.79 (m, 5H), 4.79 (d, J = 5.1 Hz, 1H), 4.69 (d, J = 5.1 Hz, 1H), 4.09 – 4.00 (m, 2H), 3.91 (s, 3H), 3.88 (s, 3H), 3.77 (s, 3H), 3.52 – 3.44 (m, 2H), 1.72 – 1.62 (m, 4H), 1.30 – 1.20 (m, 4H), 0.95 – 0.84 (m, 6H); ^{13}C NMR (101 MHz, CDCl3) δ 169.8, 150.1, 148.9, 148.8, 147.9, 130.1, 122.5, 120.6, 120.3, 116.6, 112.5, 111.0, 110.4, 83.0, 82.1, 69.4, 65.0, 55.9, 55.9, 55.9, 31.6, 30.7, 19.3, 18.9, 13.9, 13.6.

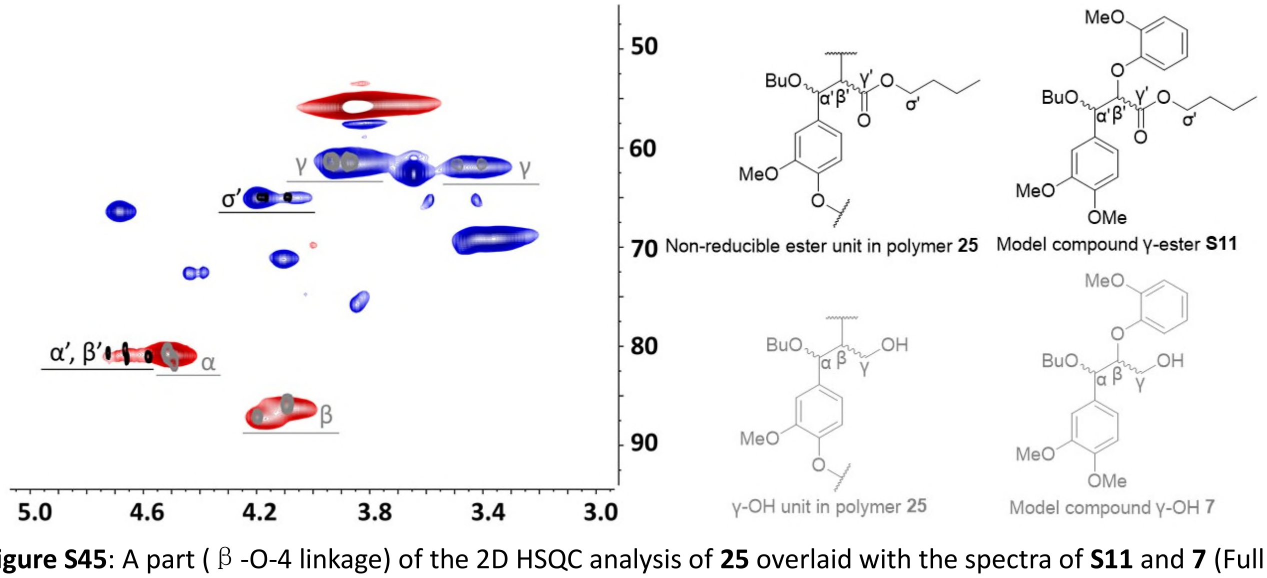

Figure S45: A part (β-O-4 linkage) of the 2D HSQC analysis of **25** overlaid with the spectra of **S11** and **7** (Full spectrum shown in **Figure S46**)

The assignment of a', β' and σ' positions of the non-reduced ester units in oligomer **25** was determined by overlaying the 2D HSQC spectrum of **25** with that of the model compound **S11**. The assignment of a, β and γ positions of γ-OH units in **25** was determined by overlaying the 2D HSQC spectrum of the known compound **7**[S15] (**Figure S45**).

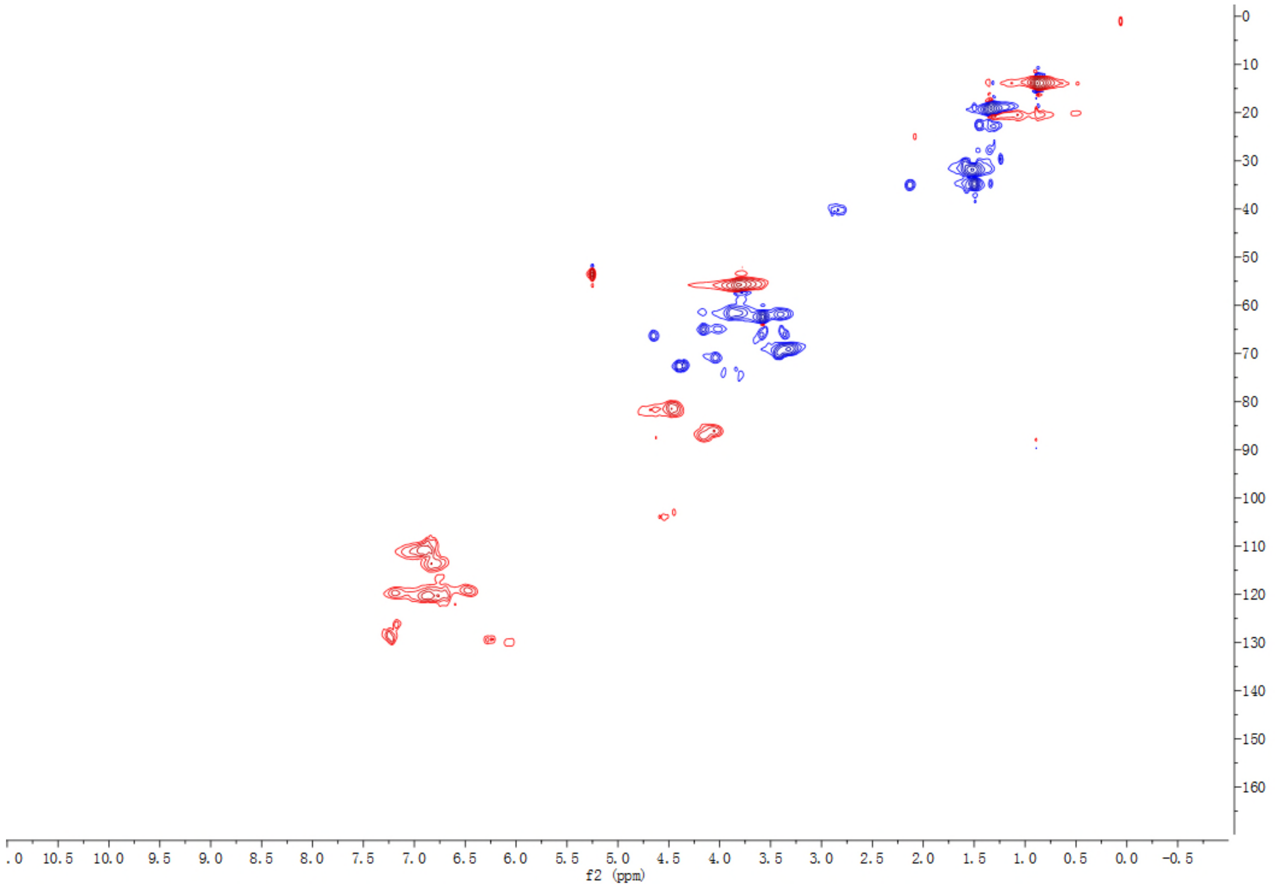

Figure S46: 2D HSQC NMR of butylated all-G β-O-4 Polymer **25**. (**Figure 4A** in manuscript, **Figure S45**)

9.4. DMP γ-Oxidation of oligomer 25

9.4.1. General procedure for the oxidation of 25

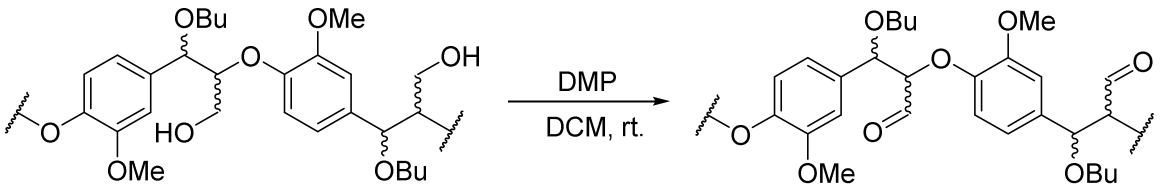

(a section of the whole structure)
All-G butanated β-O-4 polymer: **25**

(a section of the whole structure)
All-G butanated β-O-4 polymer: **26**

To a solution of **25** (1.2 g, 4.6 mmol, 1 eq.) in DCM (15 mL) was added Dess-Martin periodinane (2.3 g, 5.5 mmol, 1.2 eq.) and the reaction was stirred at room temperature for 4 hours. A mixture of sat. aqueous $Na_2S_2O_3$ solution (20 mL) and sat. aqueous $NaHCO_3$ solution (20 mL) were added and the solution was stirred rapidly for 4 hours. The organic and aqueous layers were partitioned and the aqueous layer was extracted with ethyl acetate (3 x 20 mL). The organic layers were combined, dried with Na_2SO_4 and concentrated *in vacuo*. Removal of excess DMP was achieved by column chromatography eluting with ethyl acetate: petroleum ether (10 %) and the recovery of the oligomer was achieved by washing the column with acetone to give **26** as a brown solid (900 mg, 75 %). Full 2D HSQC spectrum is shown in **Figure**

S47. Please note the aldehyde signals are folded as an extended ^{13}C dimension was not used in this and several other experiments below. See below (Figures **S50**) for a more detailed discussion of this spectrum.

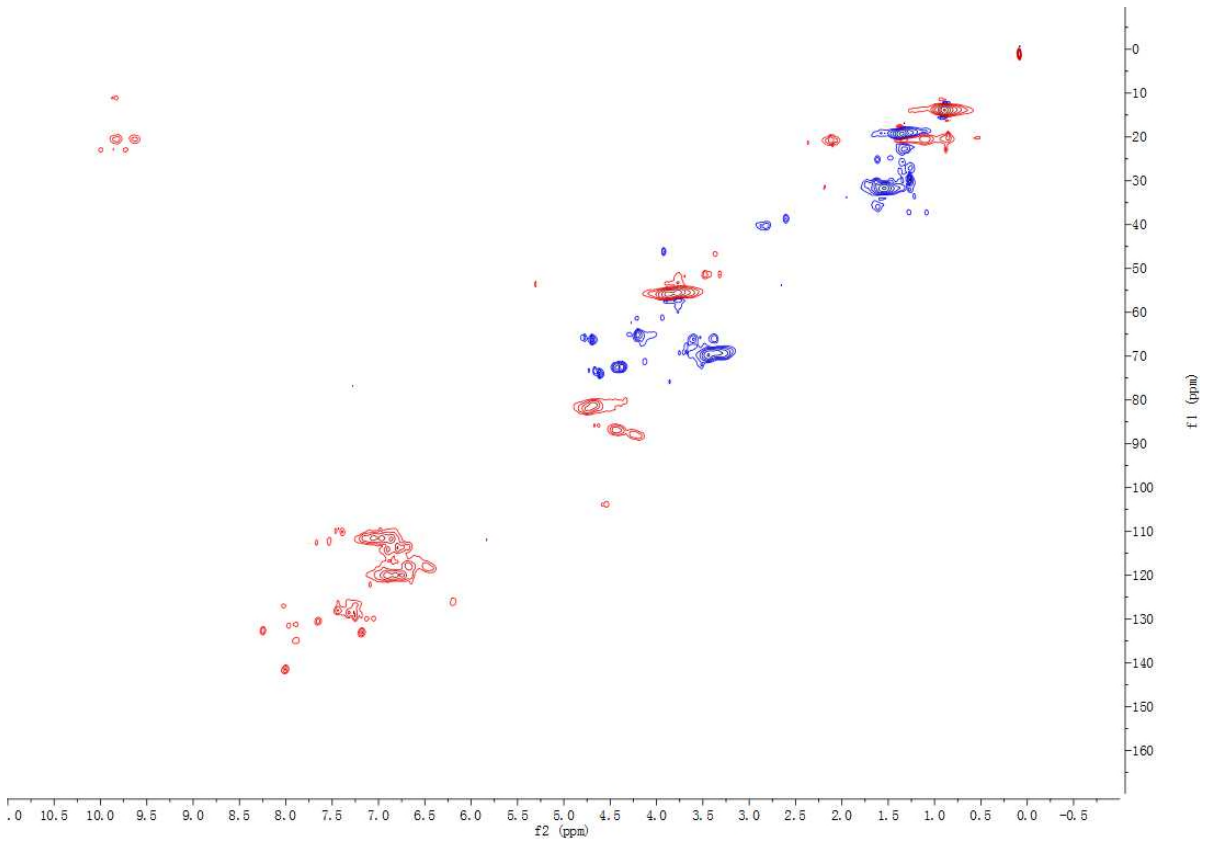

Figure S47: 2D HSQC NMR of butylated all-G β-O-4 γ-aldehyde oligomer **26**. (**Figure 4B** in manuscript and **Figure S50A/B**)

10. TEMPO oxidation of Y-CHO oligomer 26

10.1. General procedure for the TEMPO oxidation of oligomer 27

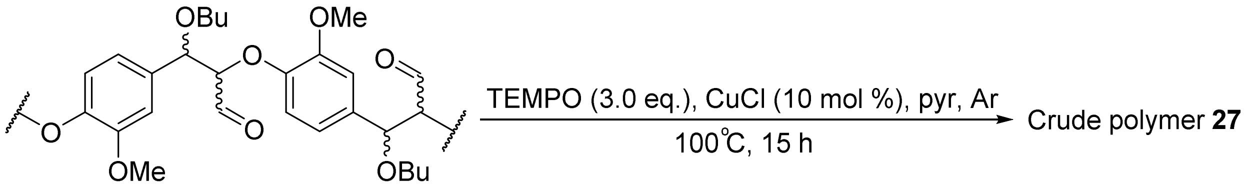

(a section of the whole structure)
All-G γ-oxidized β-O-4 polymer **26**

To a flame dried Schlenk flask under an argon atmosphere was added CuCl (10 mg, 0.075 mmol, 10 mol%). The flask was then sealed with a septum and connected to the high vacuum for 5 min to remove any remaining air, after which the flask was filled with Ar using an Ar balloon. The γ-oxidized β-O-4 oligomer **26** (200mg, 0.75 mmol, 1.0 eq.) and TEMPO (351 mg, 2.25 mmol, 3.0 eq.) in pyridine (5 mL) were added via syringe [Caution: pyridine should be freshly distilled and degassed]. The reaction was heated at 100 °C for 15 hours. After cooling to room temperature, the solvent was then removed *in vacuo*. The residue was subjected to column chromatography (10% EtOAc/Hex) to remove the excess TEMPO and the recovery of oligomer was achieved by washing the column with acetone to give oxidized oligomer **27** (210 mg, ~100 %) as a brown solid. Full 2D HSQC and 2D HMBC spectra are shown in **Figures S48** and **S49**.

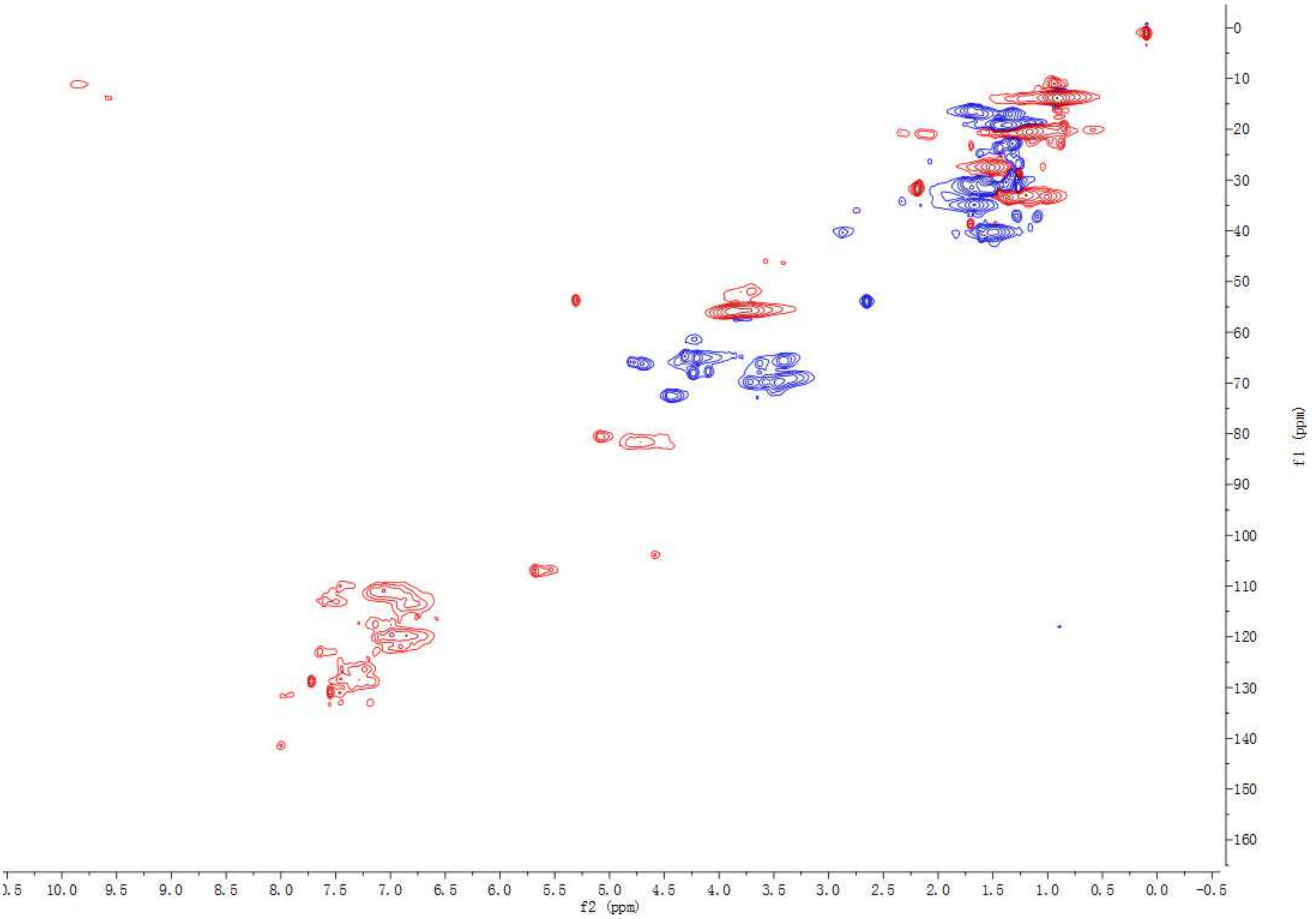

Figure S48: 2D HSQC NMR analysis of polymer **27**: CuCl (10 mol%), TEMPO (3.0 eq.), Py, Ar, 15 h (**Figure 4C** in manuscript, **Figure S50G/H** and **Figure S57B**)

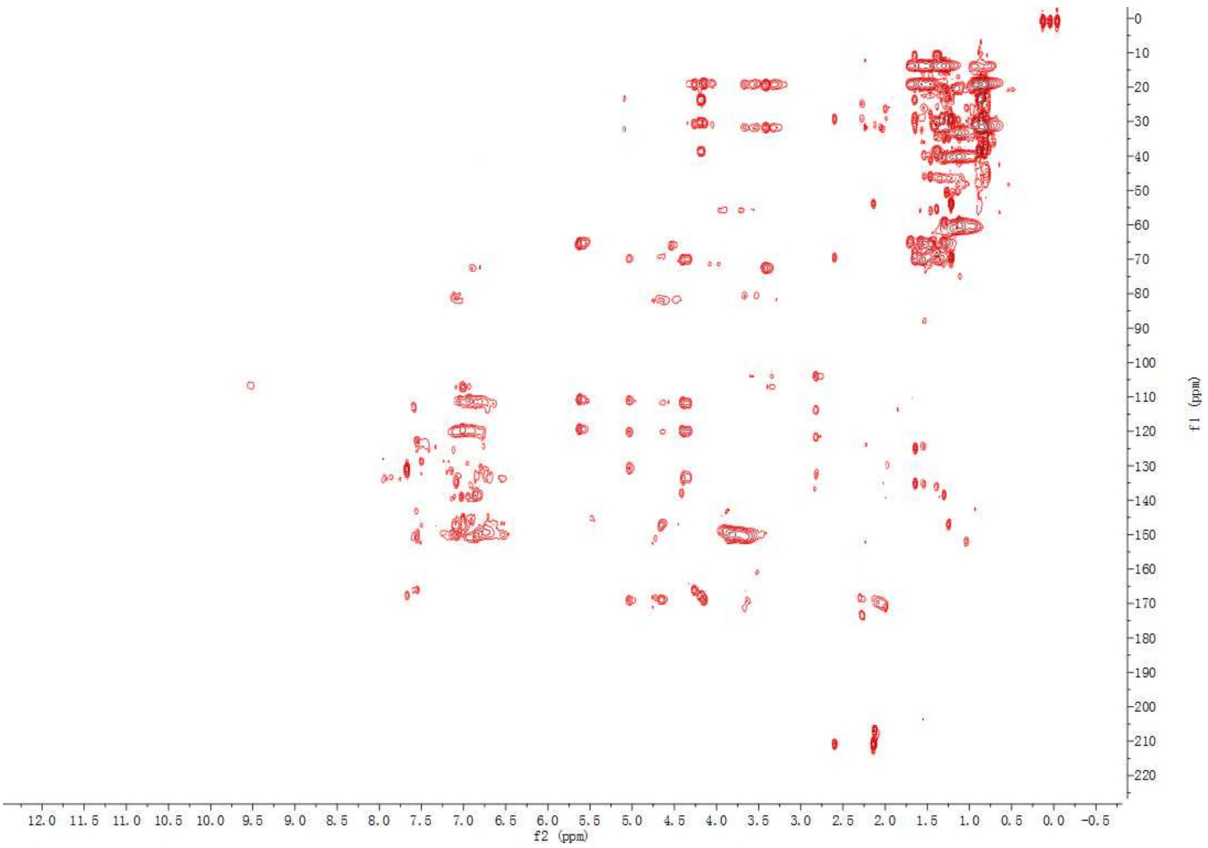

Figure S49: HMBC NMR analysis of the oligomer **27**: CuCl (10 mol%), TEMPO (3.0 eq.), Py, Ar, 15 h (**Figure S51D**).

10.2. Reaction time screening and HSQC/HMBC analysis of oligomer 27

After a reaction time of 7 and up to 10 hours, the cross-peaks corresponding to the β- (^{1}H/^{13}C δ 4.36 – 4.51 ppm/85.8 – 87.9 ppm) and γ- (^{1}H/^{13}C δ 9.56 – 9.72 ppm/199.4 – 201.3 ppm and ^{1}H/^{13}C δ 9.76 – 9.92 ppm/199.3 – 201.4 ppm) positions in starting material **26** decreased dramatically, which indicated that most of the starting material **26** had been consumed (*c.f.* **Figure S50, entry A** with **entries C** and **D**). The appearance of a new cross-peak at ^{1}H/^{13}C δ 4.98 – 5.35 ppm/ 79.5 – 84.2 ppm was particularly distinctive (**Figure S50, entry C**). This new cross-peak was assigned by overlaying with the spectra of the TEMPO-adduct model compound **22** (α-position - ^{1}H/δ^{13}C δ 5.08 – 5.30 ppm/ 82.8 – 85.1 ppm, minor diastereomer, and ^{1}H/ ^{13}C δ 5.16 – 5.23 ppm/ 80.3 – 82.5 ppm, major diastereomer, **entries B** and **C**) and the aryl ester model compound **9** (α-position: ^{1}H/^{13}C δ 5.06 – 5.12 ppm/ 79.5 – 82.0 ppm, **entry C**). However, it was difficult to tell whether the newly formed cross peak belonged to **22** or **9** or both using HSQC analysis. Further insight came from HMBC analysis of this reaction. The HMBC spectrum of the TEMPO-adduct model compound **22 (Figure S51, entry A)** showed that the α proton displayed strong ^2J and ^3J correlations to C13 (^{1}H/ ^{13}C δ 5.21 ppm/ 69.5 ppm), Cβ (^{1}H/^{13}C δ 5.21 ppm/ 105.3 ppm), C2 (^{1}H/^{13}C δ 5.21 ppm/ 111.5 ppm), C6 (^{1}H/^{13}C δ 5.21 ppm/ 122.1 ppm) and C1 (^{1}H/^{13}C δ 5.21 ppm/ 130.0 ppm), while the HMBC of the model compound aryl ester **9 (Figure S51, entry A)** showed that the α proton displayed strong ^2J and ^3J correlations to C13 (^{1}H/ ^{13}C δ 5.10 ppm/ 70.0 ppm), C2 (^{1}H/^{13}C δ 5.10 ppm/ 110.6 ppm), C6 (^{1}H/^{13}C δ 5.10 ppm/ 120.8 ppm), C1 (^{1}H/^{13}C δ 5.10 ppm/ 129.1 ppm) and Cβ (^{1}H/^{13}C δ 5.10 ppm/ 169.7 ppm). After 7 hours the HMBC analysis displayed excellent overlap of all the HMBC correlations in TEMPO-adduct **22** with the signals from **27** (**Figure S51, entry B**). In further support of this assignment, the HSQC analysis the aldehyde region (**Figure S50**, **entry D**) showed a dominant signal for the γ-position of the TEMPO-adduct (**TA**) unit (^{1}H/^{13}C δ 9.42 – 9.58 ppm/ 193.0 – 194.6 ppm). In summary, at 7 hours the TEMPO-adduct (**TA**) β-O-4 units were the major products, which was different from the dimer study results.

After 10 hours, the signals for the β- and γ-positions from the starting material **26** had completely disappeared and the new cross-peak at ^{1}H/^{13}C δ 4.93 – 5.32 ppm/ 79.3 – 84.4 ppm was becoming more dominant (**Figure S50**, **entries E**). From the HMBC analysis (**Figure S51, entry C**), two sets of signals corresponding to TEMPO-adduct units (overlay with **22**)and aryl ester units (overlay with **9**) were present, confirming that aryl ester (**AE**) units had been formed and that TEMPO-adduct (**TA**) units had not yet been fully transformed to the aryl ester (**AE**) units.

After 15 hours, the signal for the γ-position (^{1}H/^{13}C δ 9.42 – 9.58 ppm/ 193.0 – 194.6 ppm) of the TEMPO-adduct **TA** units was very weak in the 2D HSQC aldehyde region (**Figure S50, entry H** *c.f.* **entry F**) and HMBC analysis (**Figure S51, entry D**) showed that TEMPO-adduct **TA** units were apparently not present with aryl ester units dominating the spectrum (overlay with aryl ester **9**). This confirmed that nearly all the TEMPO-adduct **TA** units have been converted to the aryl ester **AE** units by this time.

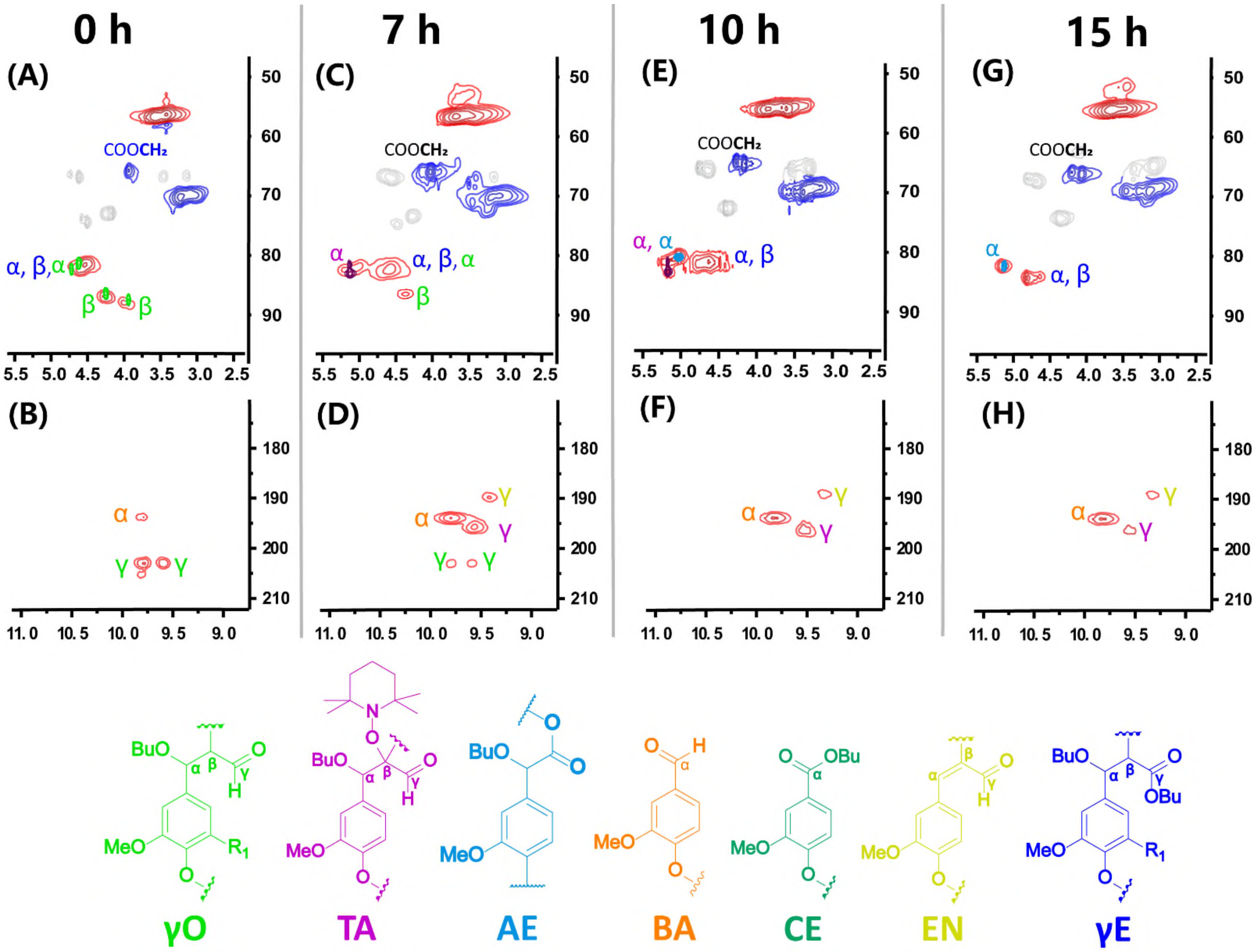

Figure S50: 2D HSQC NMR analysis of linkage (^{1}H/ ^{13}C δ 2.50 – 5.50 / 50.0 – 90.0 ppm) and aldehyde region (^{1}H/ ^{13}C δ 9.0 – 10.0 / 180.0 – 210.0 ppm) of oligomer **27** at different time points on treatment with CuCl (10 mol%), TEMPO (3.0 eq.), Py under an argon atmosphere. Full spectra shown in **Figures S47**, **S48, S53 and S55.**

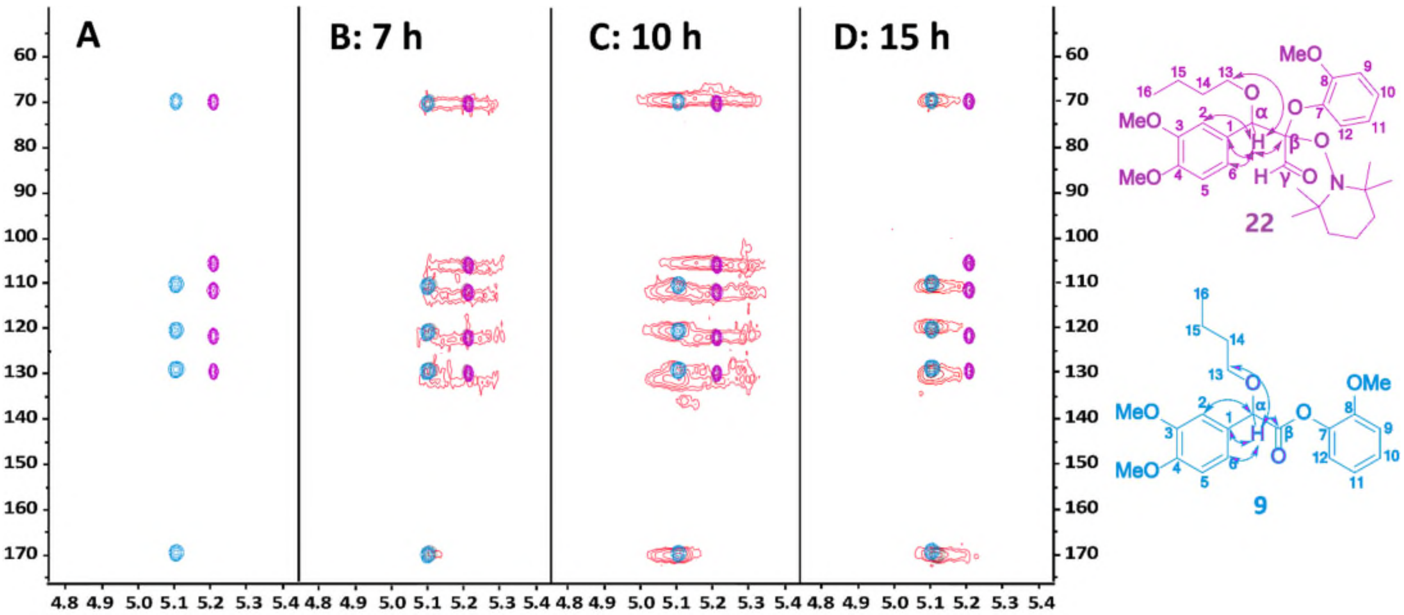

Figure S51: Selected regions of the following HMBC analysis of: (**A**) ^{13}C correlations to the α-proton of TEMPO-adduct model compound **22** (purple) and the α-proton of aryl ester model compound **9** (blue); (**B**, **C** and **D**) TEMPO-oxidized oligomer **27** at different time points overlaid with spectra of models **9** and **22**. Full spectra are shown in **Figures S49**, **S54** and **S56**. The time course shows that with increasing time TEMPO-adduct units (modelled by **22**) in **27** are converted with time to the corresponding esters (modelled by **9**).

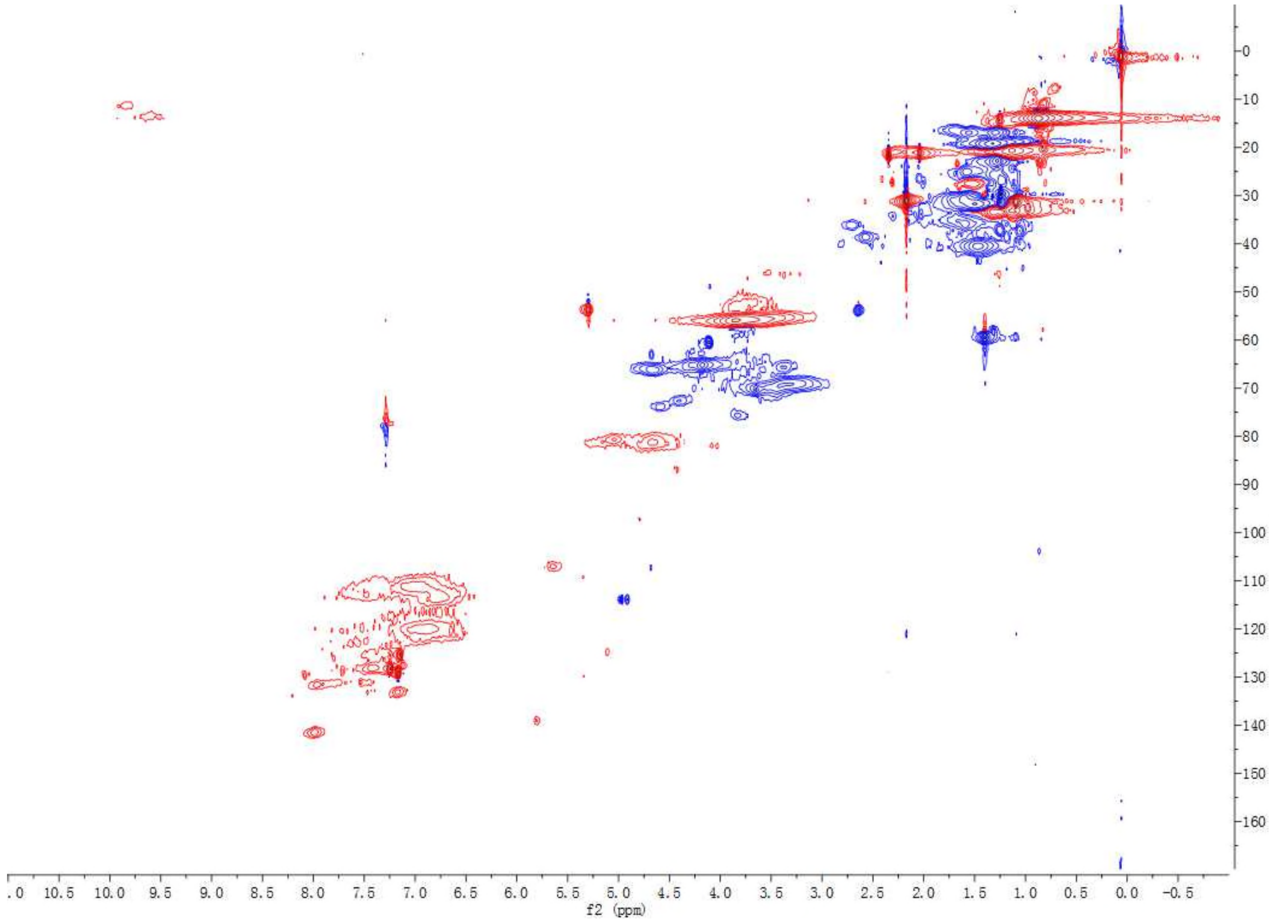

Figure S52: HSQC analysis of **27** after TEMPO oxidation: CuCl (10 mol%), TEMPO (2.0 eq.), Py, Ar, 7 h.

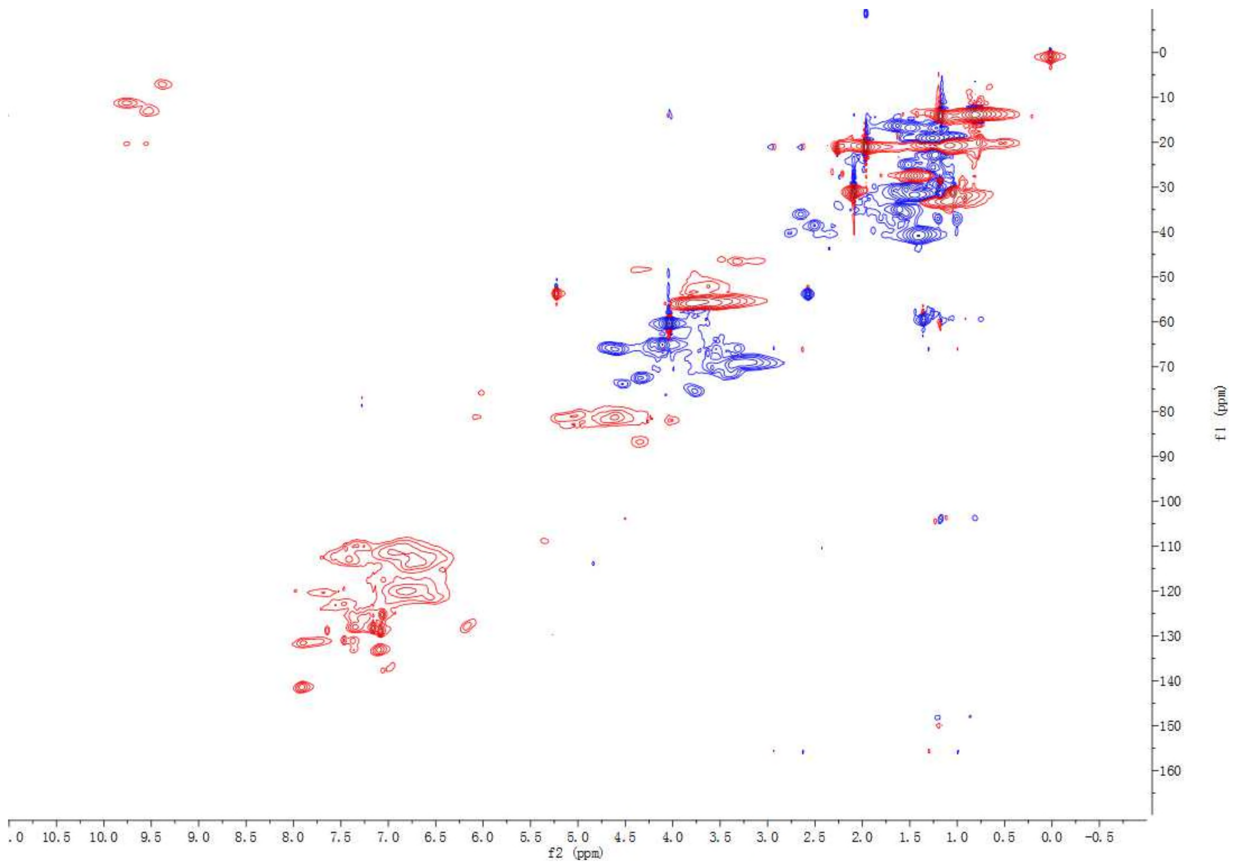

Figure S53: HSQC analysis of the oligomer **27**: CuCl (10 mol%), TEMPO (3.0 eq.), Py, Ar, 7 h: (**Figure S50**, **entries C/D**)

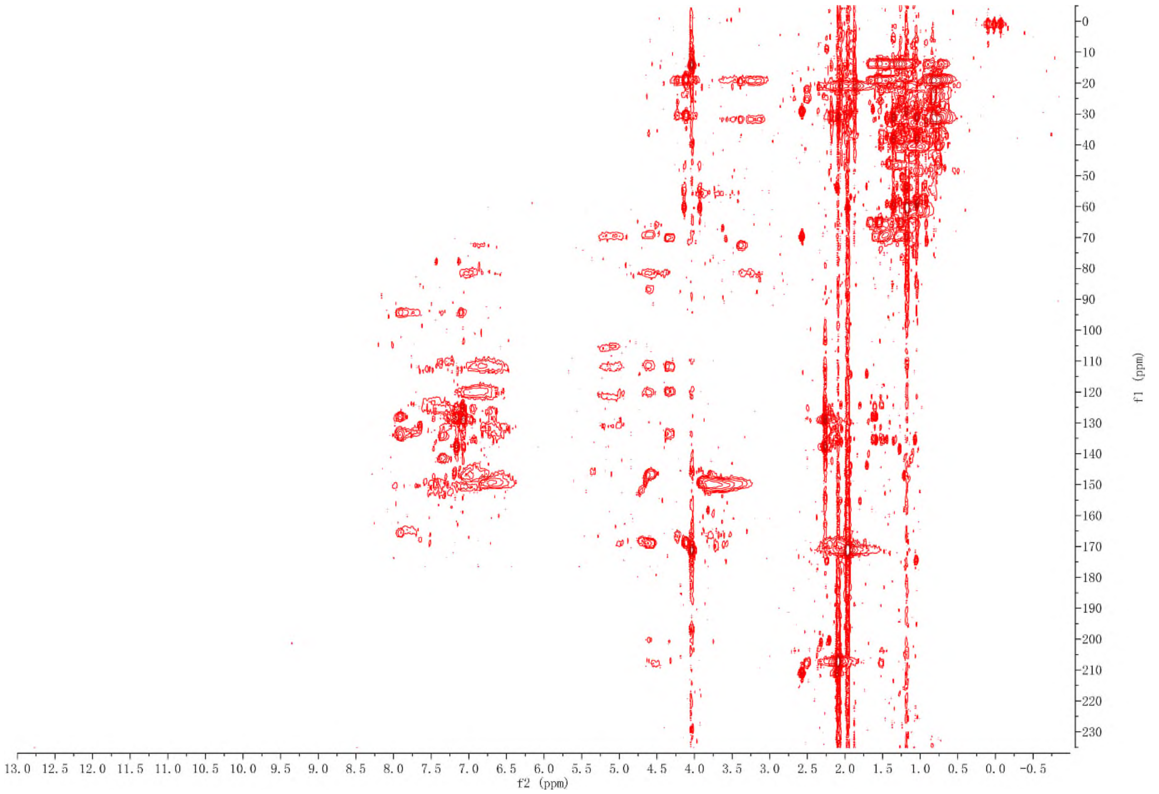

Figure S54: HMBC analysis of **27** after TEMPO oxidation: CuCl (10 mol%), TEMPO (3.0 eq.), Py, Ar, 7 h: (**Figure S51, entry B**)

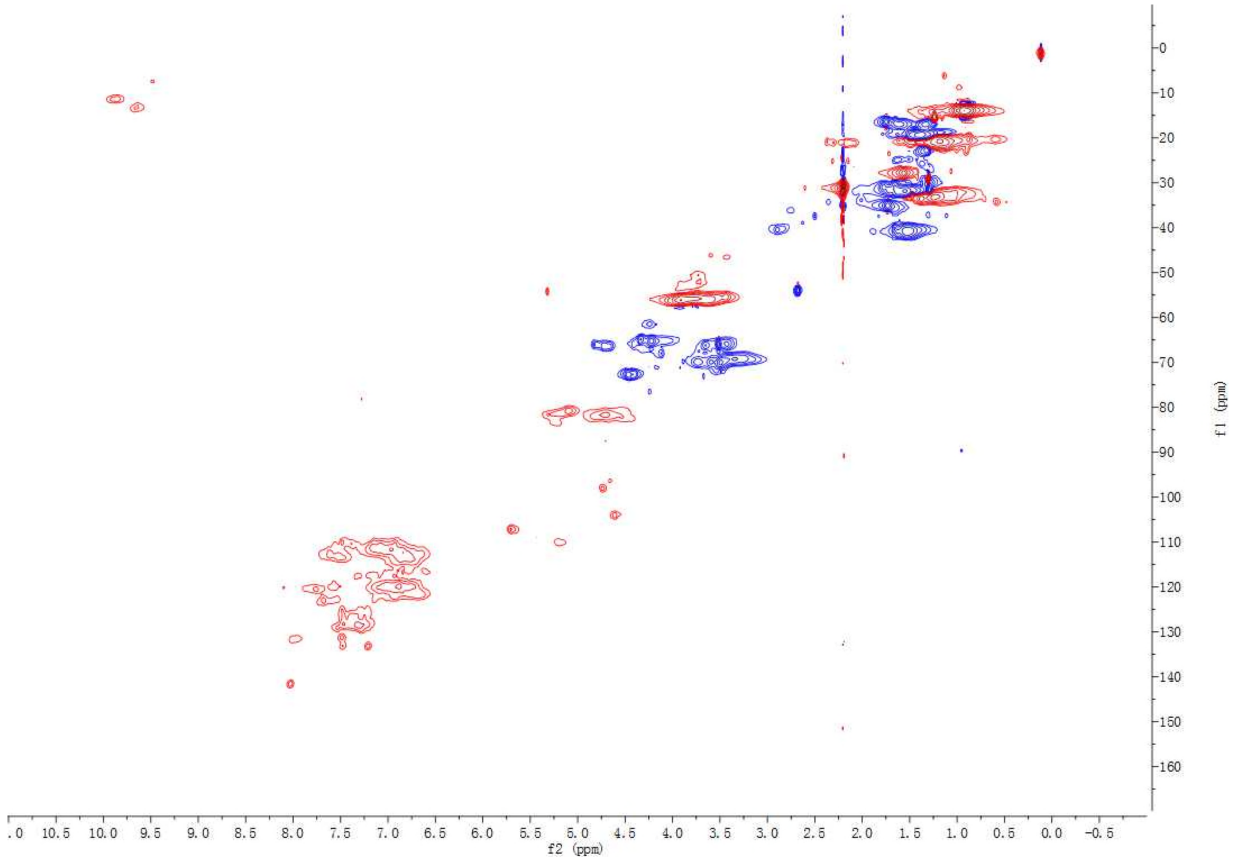

Figure S55: HSQC analysis of **27** after TEMPO oxidation: CuCl (10 mol%), TEMPO (3.0 eq.), Py, Ar, 10 h (**Figure S50, entries E/F**).

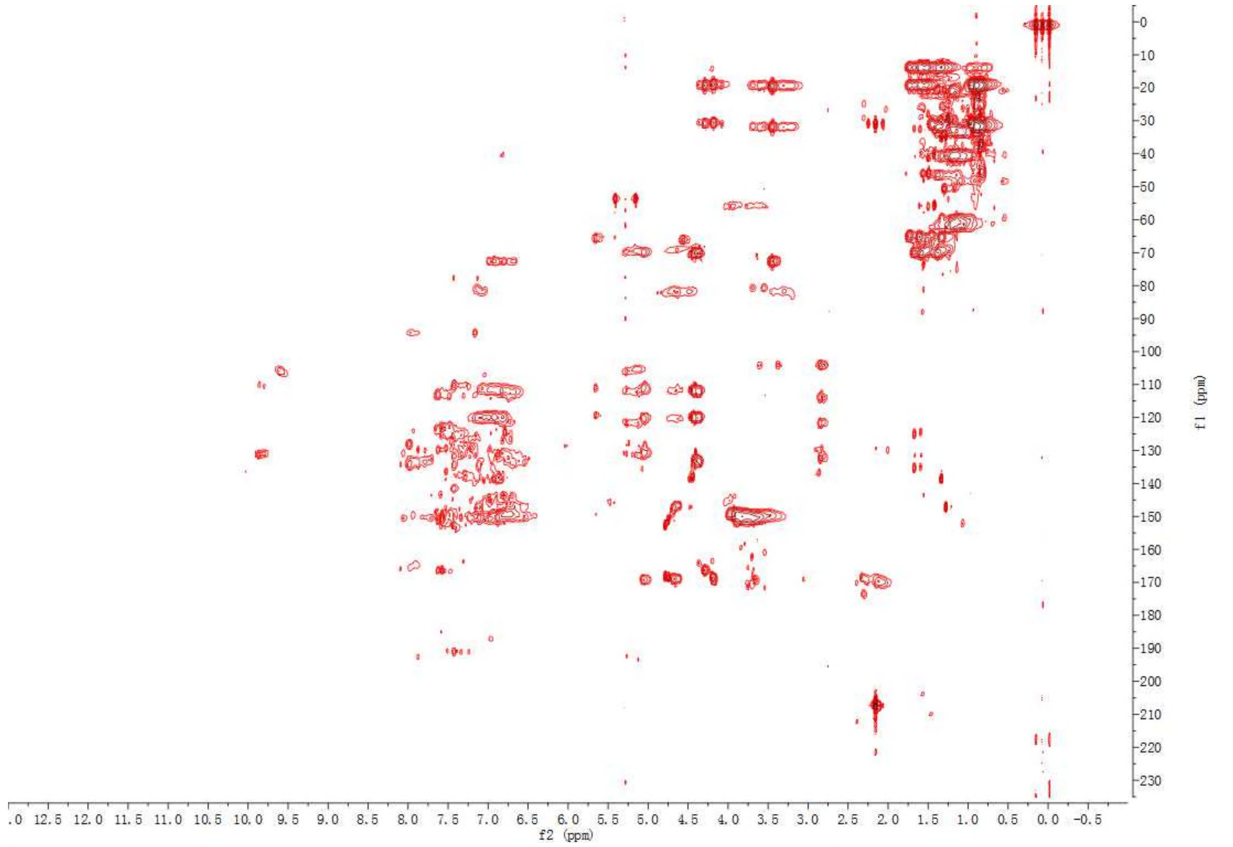

Figure S56: HMBC analysis of **27** after TEMPO oxidation: CuCl (10 mol%), TEMPO (3.0 eq.), Py, Ar, 10 h. (**Figure S51C**)

10.3. Screening the amounts of TEMPO used to form oligomer 27 form 26

Apart from the aryl ester **AE** units, there were several other fragments formed during the TEMPO oxidation process of **26**. HSQC analysis of **27** (**Figure S57**) had distinct cross-peak at: (1) $^{1}H/ ^{13}C$ δ 9.33 – 9.57/ 186.1 – 188.3 ppm corresponding to the γ-position of enal **EN** units; (2) $^{1}H/ ^{13}C$ δ 7.60 – 7.67 ppm/ 122.1 – 123.8 ppm corresponding to the H6 of cleaved butyl ester **CE** units; (3) $^{1}H/ ^{13}C$ δ 9.69 – 9.99 ppm/ 190.1 – 192.2 ppm corresponding to the α-position of benzyl aldehyde **BA** units formed from either cleaved benzyl aldehyde units or the end groups formed in the DMP-oxidation step.

In the study using dimer **8**, the formation of aryl ester **9** (**Figure 3C** in manuscript) was proposed to require 2.0 eq. TEMPO, and an increase in the amount of TEMPO from 2.0 eq. to 3.0 eq. only led to a slight increase in the yield. However, these results were not fully consistent with those found in the oligomer study. Semi-quantitative integral analysis of the relevant regions in the HSQC gave an approximated 1: 1.37: 1.97: 1.45 ratio of aryl ester **AE**: benzyl aldehyde **BA**: butyl ester **CE**: enal **EN** units in **27** when 2.0 eq. TEMPO was used (**Figure S57, entry A**); When 3.0 eq. TEMPO was used the observed ratio was 1: 0.34: 0.29 of aryl ester **AE**: benzyl aldehyde **BA**: butyl ester **CE** with no enal **EN** units being detectable (**entry B**). An increase in the amount of TEMPO to 3.0 eq. therefore leads to a significant increase in the amount of aryl ester **AE** present in **27**. Considering the presence of γ-ethyl ester **γE,** the percentage of aryl ester **AE** in **27** was estimated to be 40 % (100 % × 1 / (1 + 0.29 + 0.34 + 0.9) = 40 %).

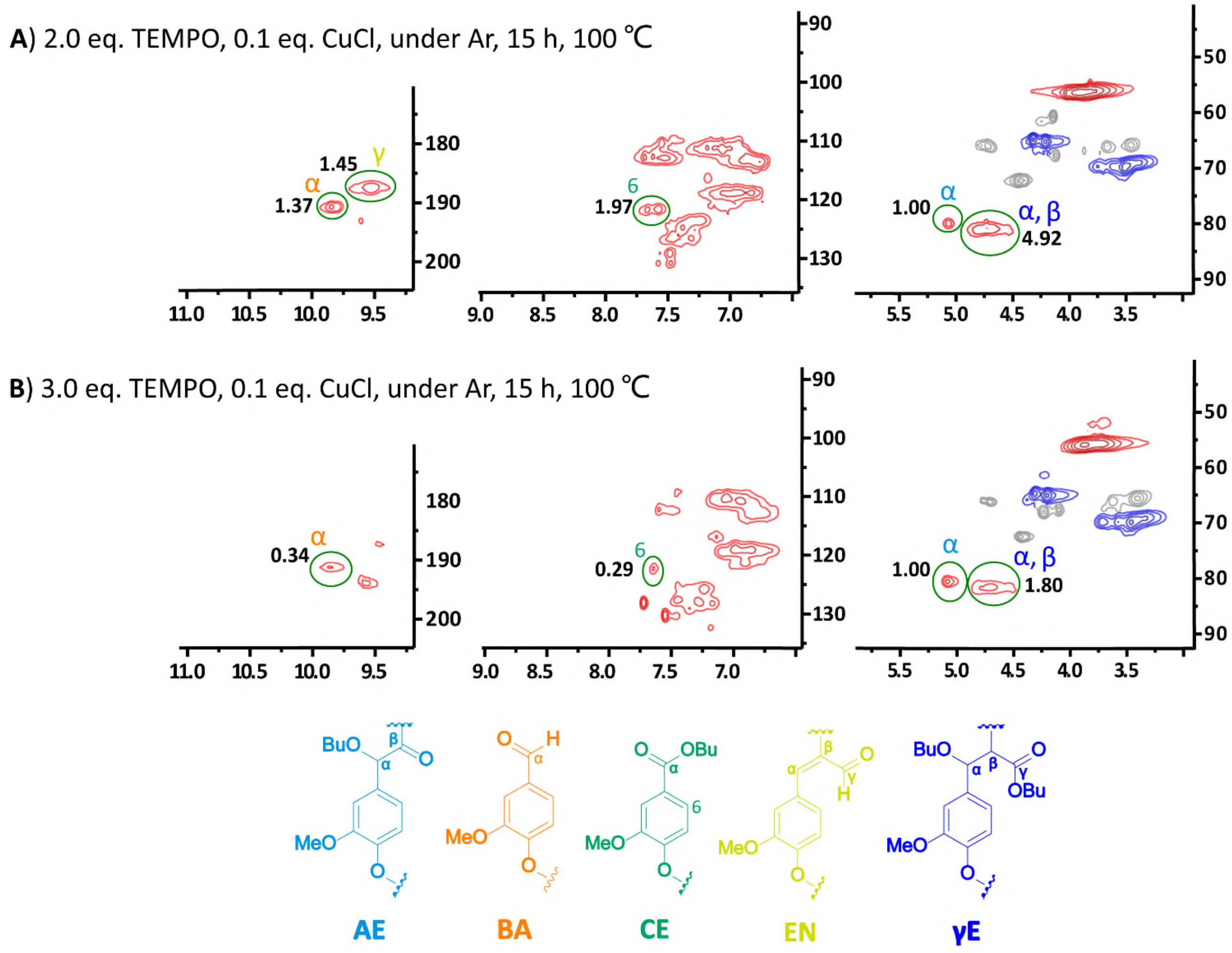

Figure S57: HSQC analysis of **27** after oxidation with: (**A** 2.0 eq. TEMPO, 0.1 eq. CuCl, under argon atmosphere, 15 h; (**B**) 3.0 eq. TEMPO, 0.1 eq. CuCl, under argon atmosphere, 15 h. Full spectra shown in **Figures S48** and **S58.**

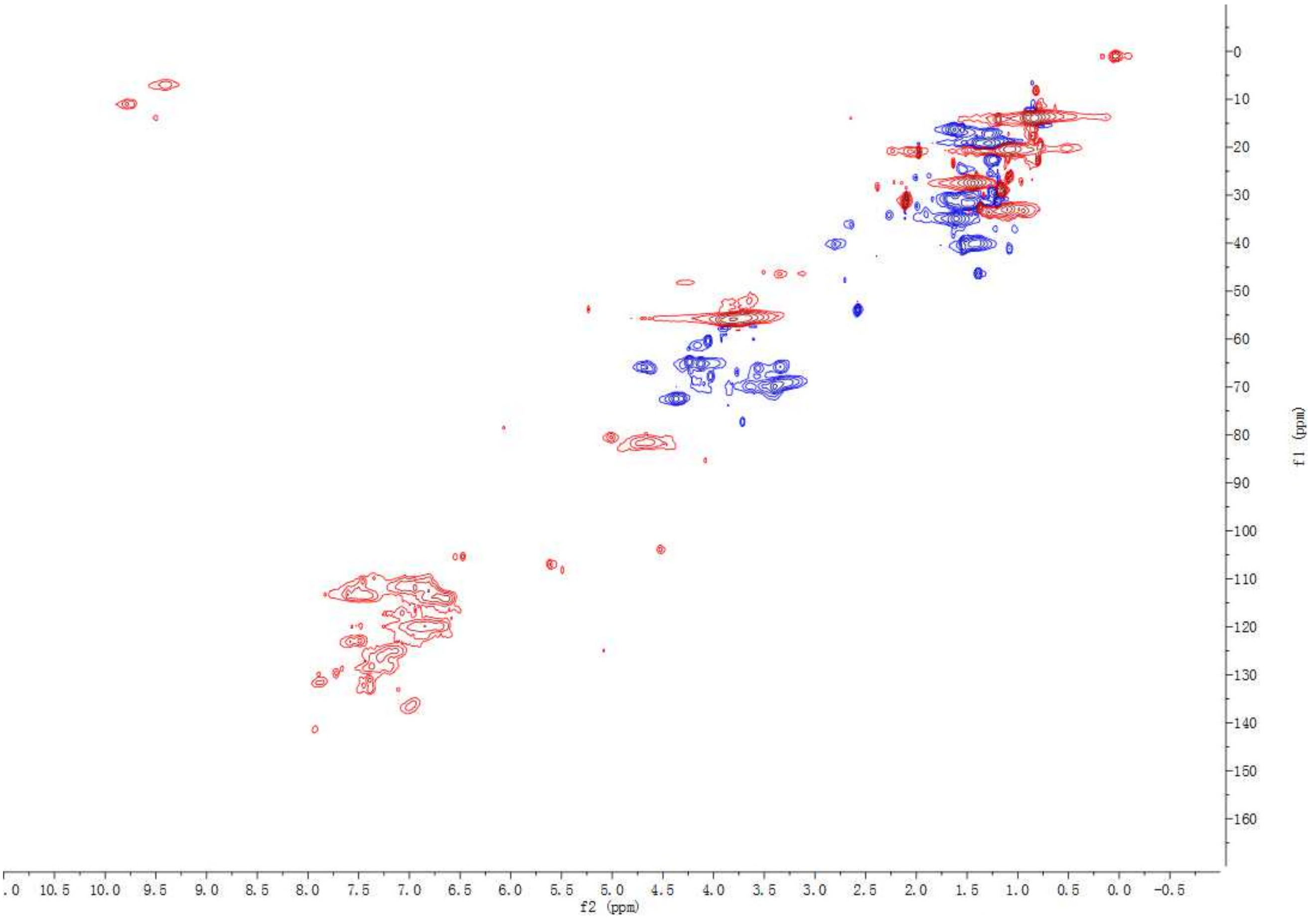

Figure S58: HSQC analysis of **27** after TEMPO oxidation: CuCl (10 mol%), TEMPO (2.0 eq.), Py, Ar, 15 h (**Figure S57A**)

11. Hydrolysis of oxidized polymer 27

11.1. General procedure

The oligomer **27** (200 mg) was dissolved in a solution of NaOH in methanol (0.5 M, 5 mL) and stirred overnight at room temperature. The mixture was acidified with 10 % aqueous HCl solution (5 mL) and then extracted with ethyl acetate (3 x 10 mL). The organic extracts were combined, washed with brine, dried (Na_2SO_4) and concentrated *in vacuo*. Full 2D HSQC analysis is shown in **Figure S60**. Purification was achieved by column chromatography (10% EtOAc/Hex. to 100% actone). Three main fractions were obtained.

Fraction 1:

4-Hydroxy-3-methoxybenzaldehyde (S10)[S15]**:** 6 mg, 3.0 wt %, a white solid. **m.p**. 78 - 81 °C (lit.[S15] 82 - 83 °C). ^{1}H NMR (500 MHz, $CDCl_3$) δ 9.85 (s, 1H, C**H**O), 7.49 – 7.42 (m, 2H, H5 and H6), 7.09 – 7.03 (m, 1H, H2), 6.27 (s, 1H, O**H**), 3.99 (s, 3H, OC**H₃**). Analytical data was in accordance with that previously reported.[S16]

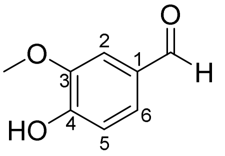

S10

Fraction 2:

2-Butoxy-2-(4-hydroxy-3-methoxyphenyl) acetic acid (28): 14 mg, 7.0 wt %, a colourless oil. **HRMS** (ESI) calculated for $C_{13}H_{17}O_5$ 253.1076, $[M-H]^-$, found 253.1078; **IR** (thin film) 2961, 1719, 1603, 1492, 1275, 908, 816, 780 cm^{-1}; **^{1}H NMR** (500 MHz, $CDCl_3$) δ 7.85 (s, 1H, COO**H**), 6.99 – 6.89 (m, 2H, H2 and H6), 6.90 (d, J = 8.1 Hz, 1H, H5), 5.31 (s, 1H, O**H**), 4.80 (s, 1H, Hα), 3.87 (s, 3H, OC**H$_3$**), 3.58 – 3.42 (m, 2H, OC**H$_2$**CH$_2$), 1.68 – 1.56 (m, 2H, OCH$_2$C**H$_2$**), 1.43 – 1.34 (m, 2H, C**H$_2$**CH$_3$), 0.91 (t, J = 7.3 Hz, 3H, CH$_2$C**H$_3$**). **^{13}C NMR** (126 MHz, $CDCl_3$) δ 175.7 (Cβ), 146.9 (C3), 146.2 (C4), 128.0

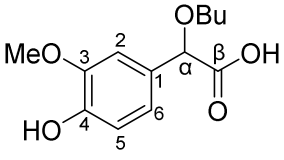

28

(C1), 120.9 (C6), 114.7 (C5), 109.4 (C2), 80.4 (Cα), 69.4 (O**C**H_2CH_2), 55.9 (O**C**H_3), 31.4 (OCH_2**C**H_2), 19.6 (**C**H_2CH_3), 13.8 (CH_2**C**H_3).

Fraction 3:
inseparable mixture, 25 mg, 13.0 wt %. HRMS analysis (**Figure S59**) showed the existence of two dimers tentatively assigned to structures **S11** and **S12**.

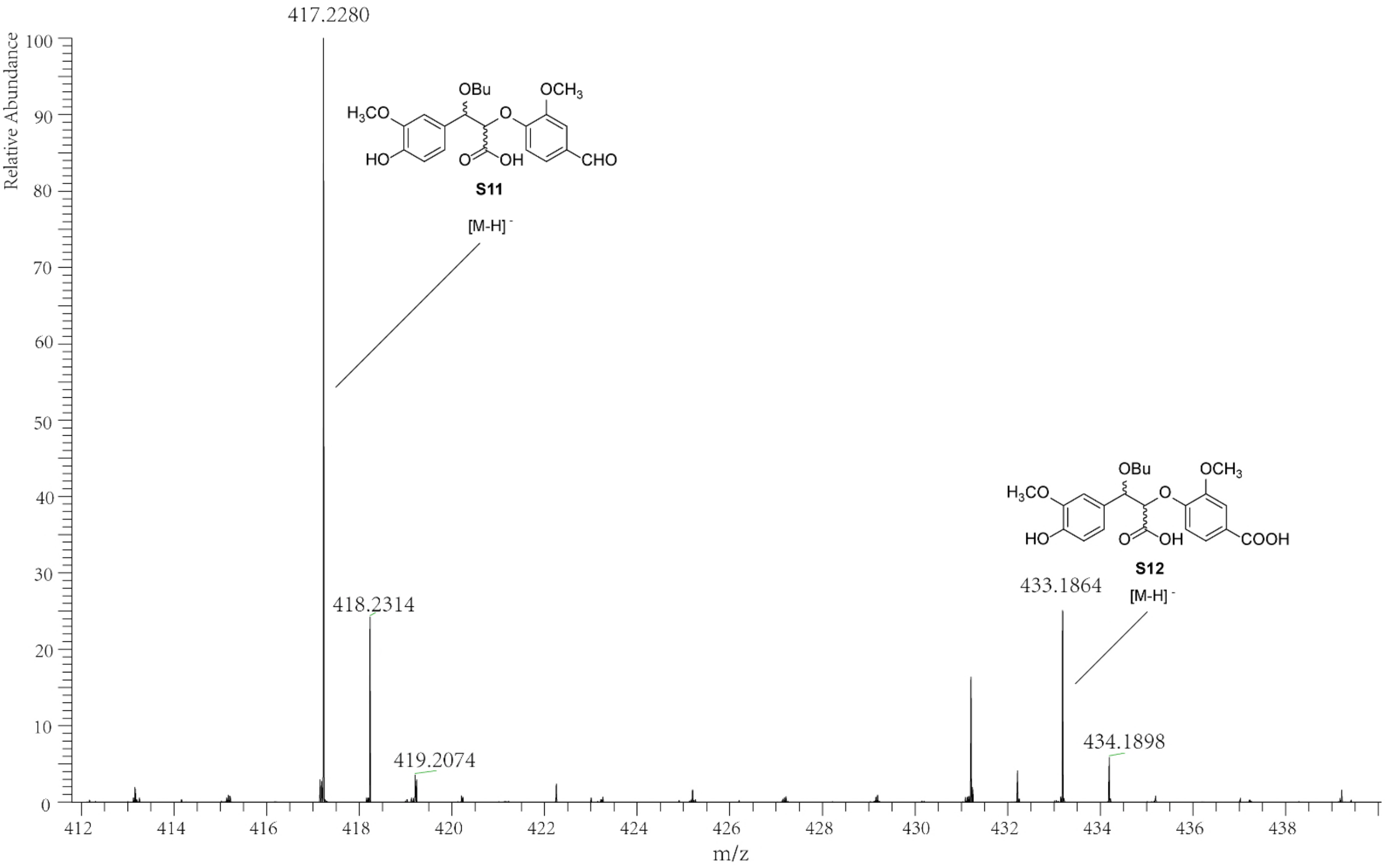

Figure S59: HRMS analysis of **Fraction 3**.

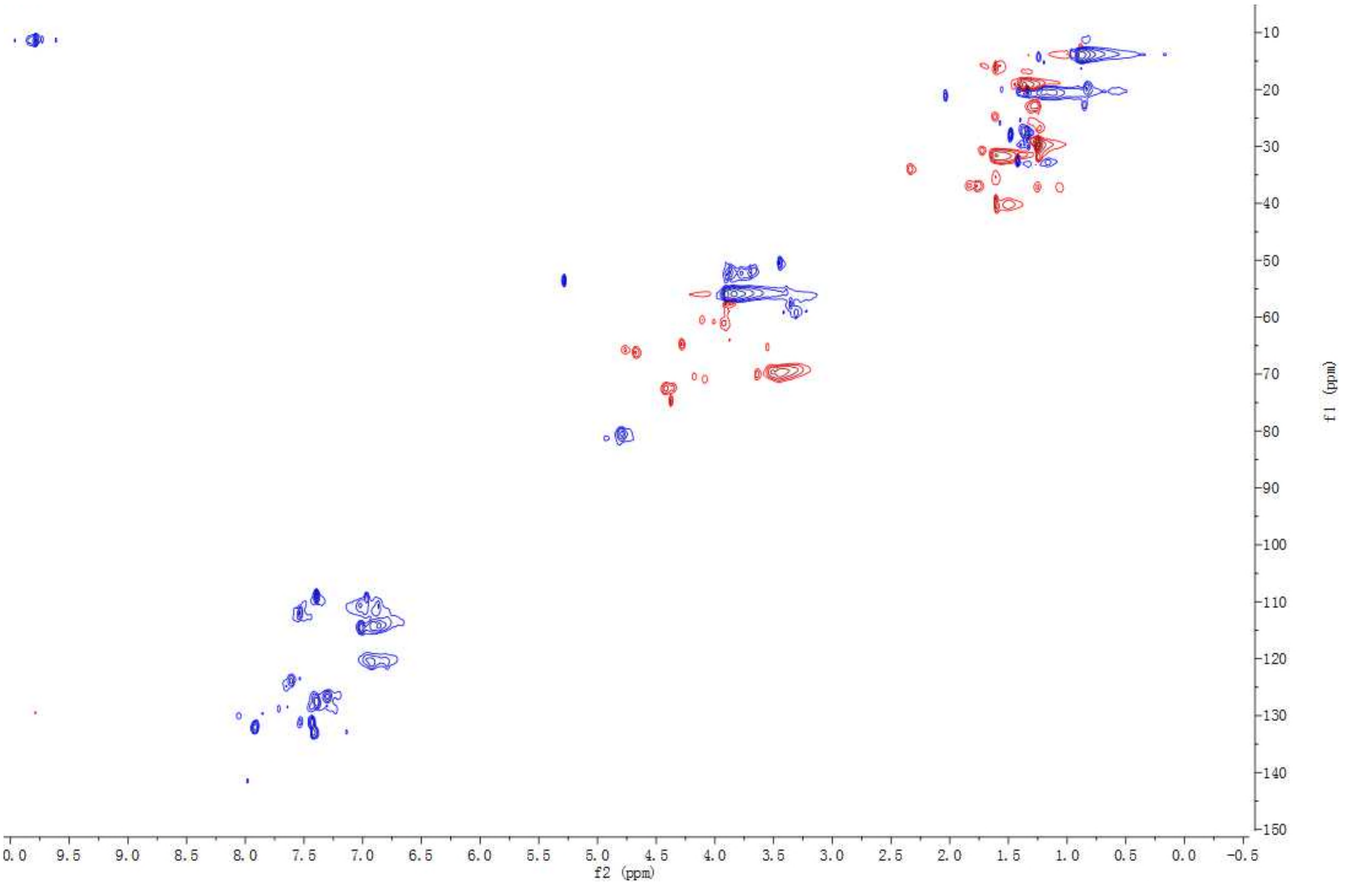

Figure S60: 2D HSQC NMR analysis of crude mixture after hydrolysis of oligomer **27** (**Figure 4D** in manuscript).

11.2. Rationalization of the depolymerization process to obtain monomers and dimers.

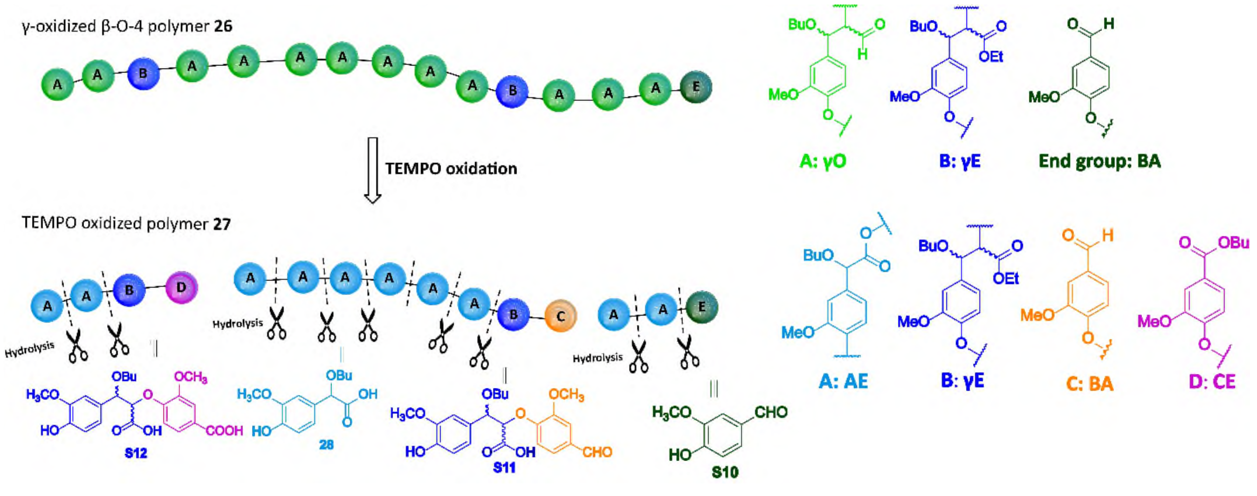

Figure S61: After the TEMPO oxidation, most of the γ-aldehyde units γ **O** have been converted to the ester units **AE**, while the parallel cleavage reactions lead to the depolymerization with **BA** and **CE** being the possible end groups. After the hydrolysis, a monomer **S10** can be released when, for example, an **AE** unit and a benzyl aldehyde unit (**BA** or the end group formed during DMP γ-oxidation) are next to each other; a dimer such as **S11** can be released when a **AE** unit, a **γE** unit and a benzyl aldehyde unit (**BA** or the end group formed during DMP γ-oxidation) are next to each other and the ethyl ester group in the **γE** unit has been hydrolysed; a dimer **S12** can be released when a **AE** unit, a **γE** unit and a **CE** unit are next to each other and the ethyl and butyl ester groups in the **γE** and **CE** units respectively have been hydrolysed.

The release of a monomer **28** requires 2 ester units **AE** to be contiguous. Based on the calculation in **Section 10.3**, the possibility of 2 **AE** units being contiguous is 16% (40% x 40% = 16%). As the released monomers from oligomer **27** have the same level of molecular weight as a unit in the oligomer chain[S12], a maximal yield of 16 wt% of **28** would be expected. Thus the 7.0 wt % yield of isolated product **28** given the theoretical yield 16.0 wt % of **28** could be stated as a 44% yield.

12. NMR spectra of synthesized compounds

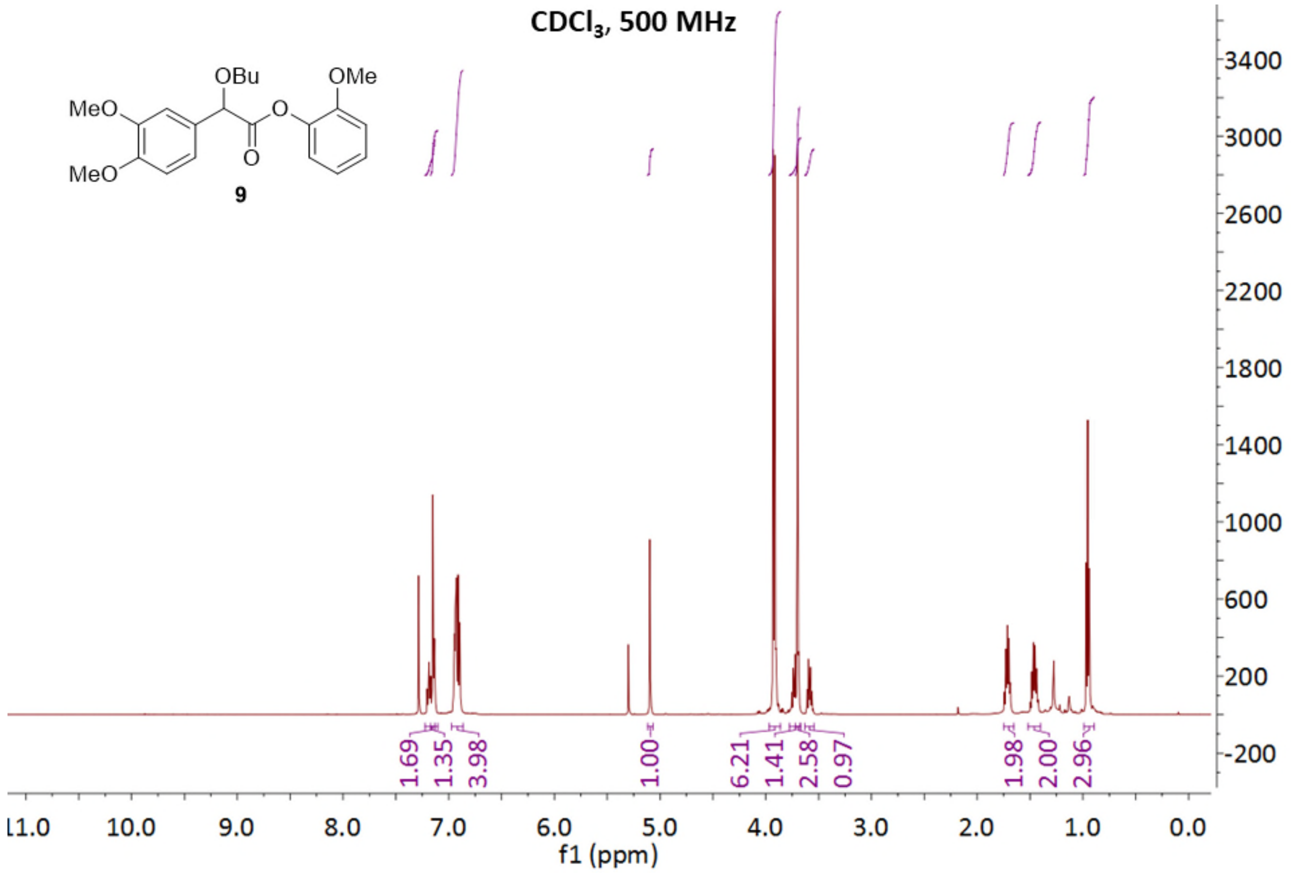

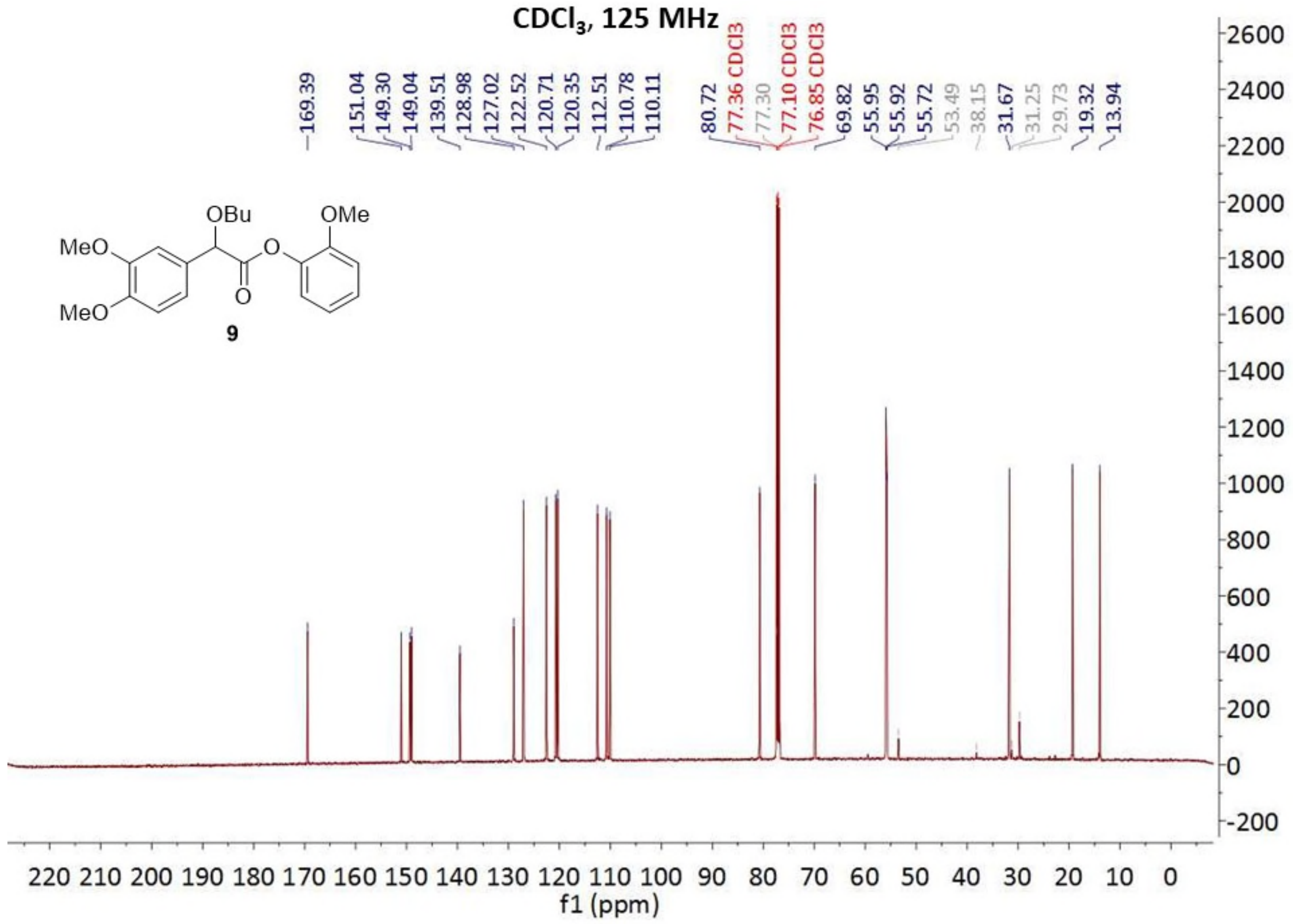

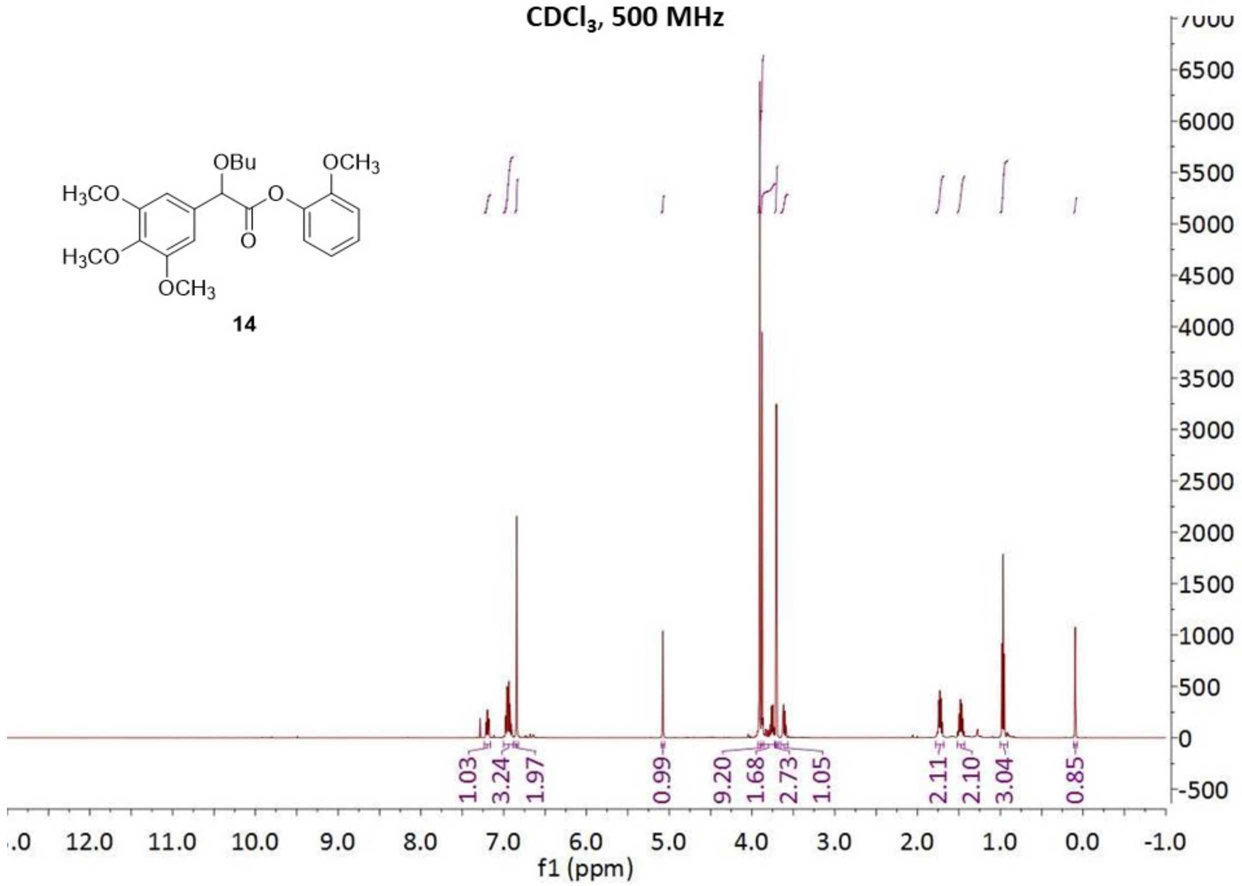
CDCl3, 500 MHz
OBu
OCH3
H3CO
H3CO
OCH3
O
14
1.03
3.24
1.97
0.99
9.20
1.68
2.73
1.05
2.11
2.10
3.04
0.85
f1 (ppm)

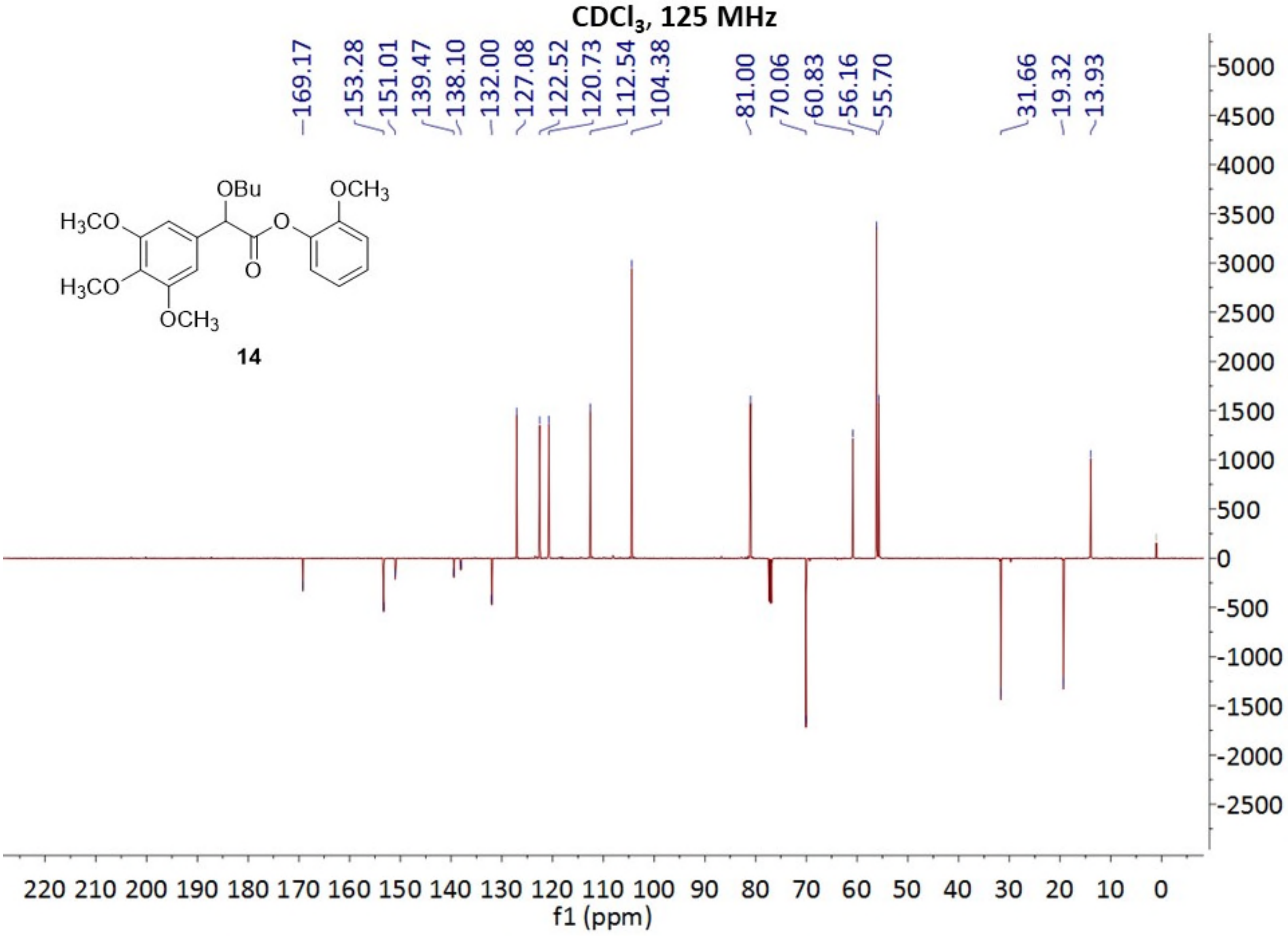
CDCl3, 125 MHz
169.17
153.28
151.01
139.47
138.10
132.00
127.08
122.52
120.73
112.54
104.38
81.00
70.06
60.83
56.16
55.70
31.66
19.32
13.93
OBu
OCH3
H3CO
H3CO
OCH3
O
14
f1 (ppm)

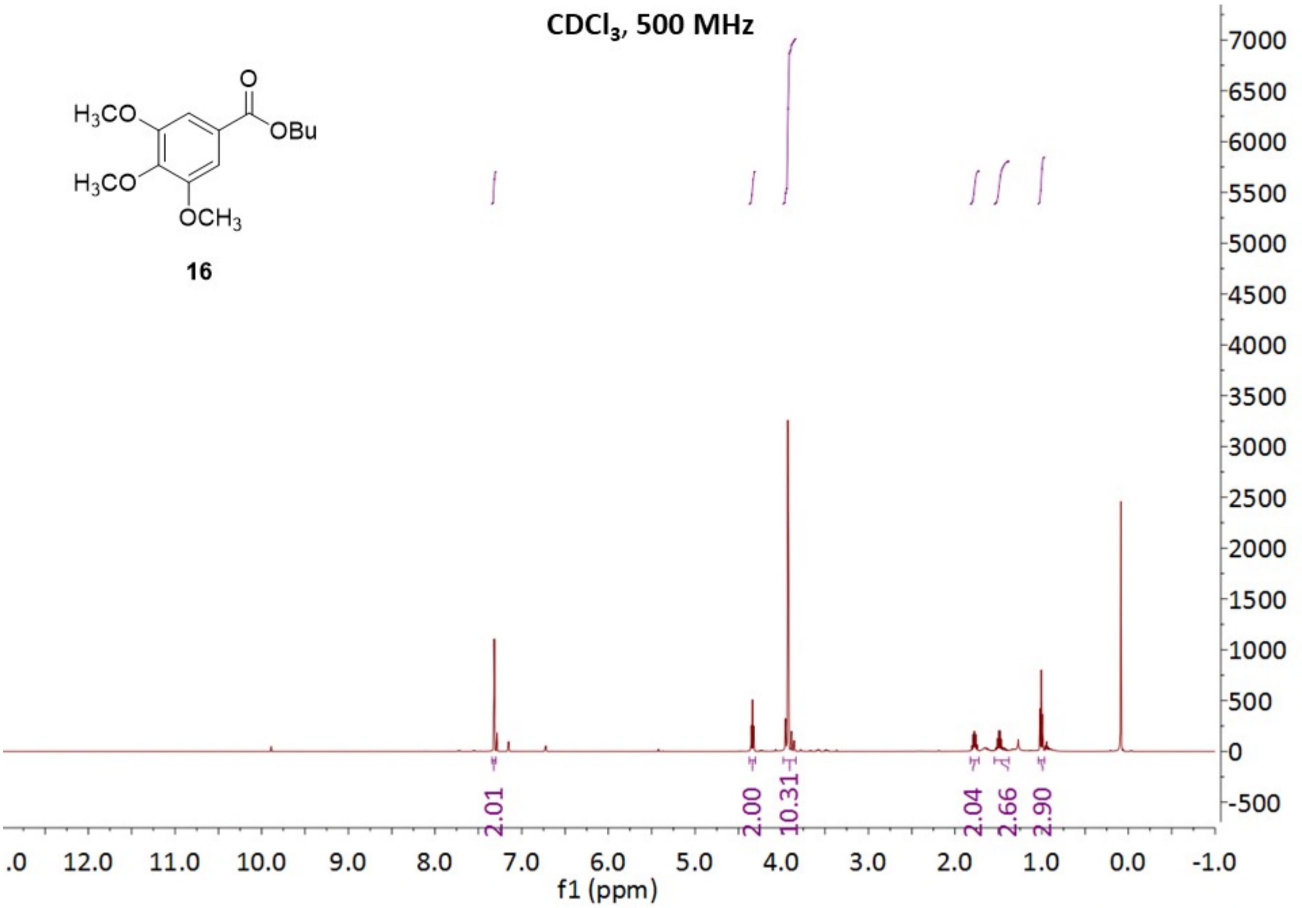
CDCl3, 500 MHz
H3CO
H3CO
OCH3
OBu
O
16
2.01
2.00
10.31
2.04
2.66
2.90
f1 (ppm)

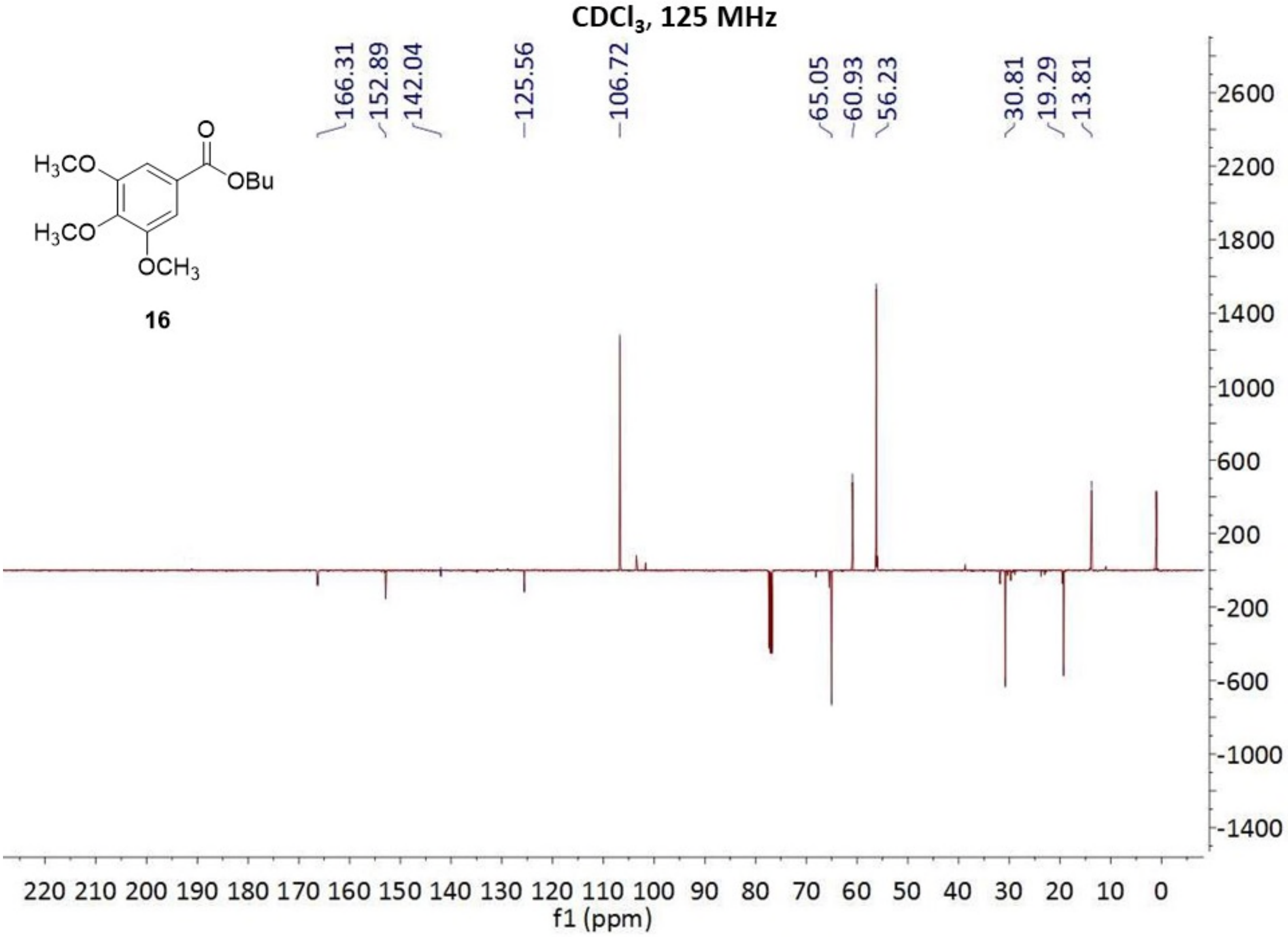
CDCl3, 125 MHz
166.31
152.89
142.04
125.56
106.72
65.05
60.93
56.23
30.81
19.29
13.81
H3CO
H3CO
OCH3
OBu
O
16
f1 (ppm)

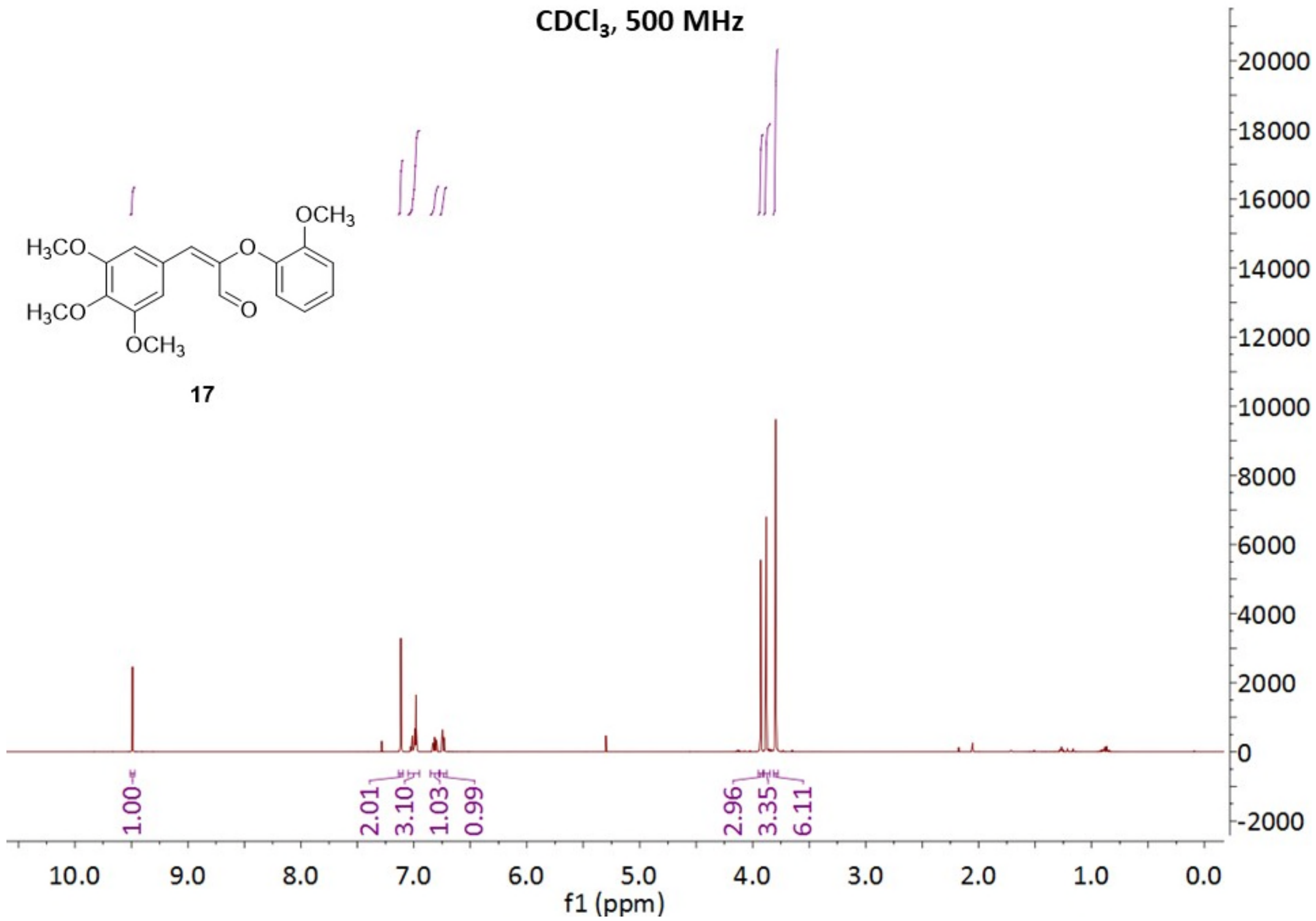
CDCl3, 500 MHz
OCH3
H3CO
H3CO
OCH3
O
O
17
1.00
2.01
3.10
1.03
0.99
2.96
3.35
6.11
f1 (ppm)

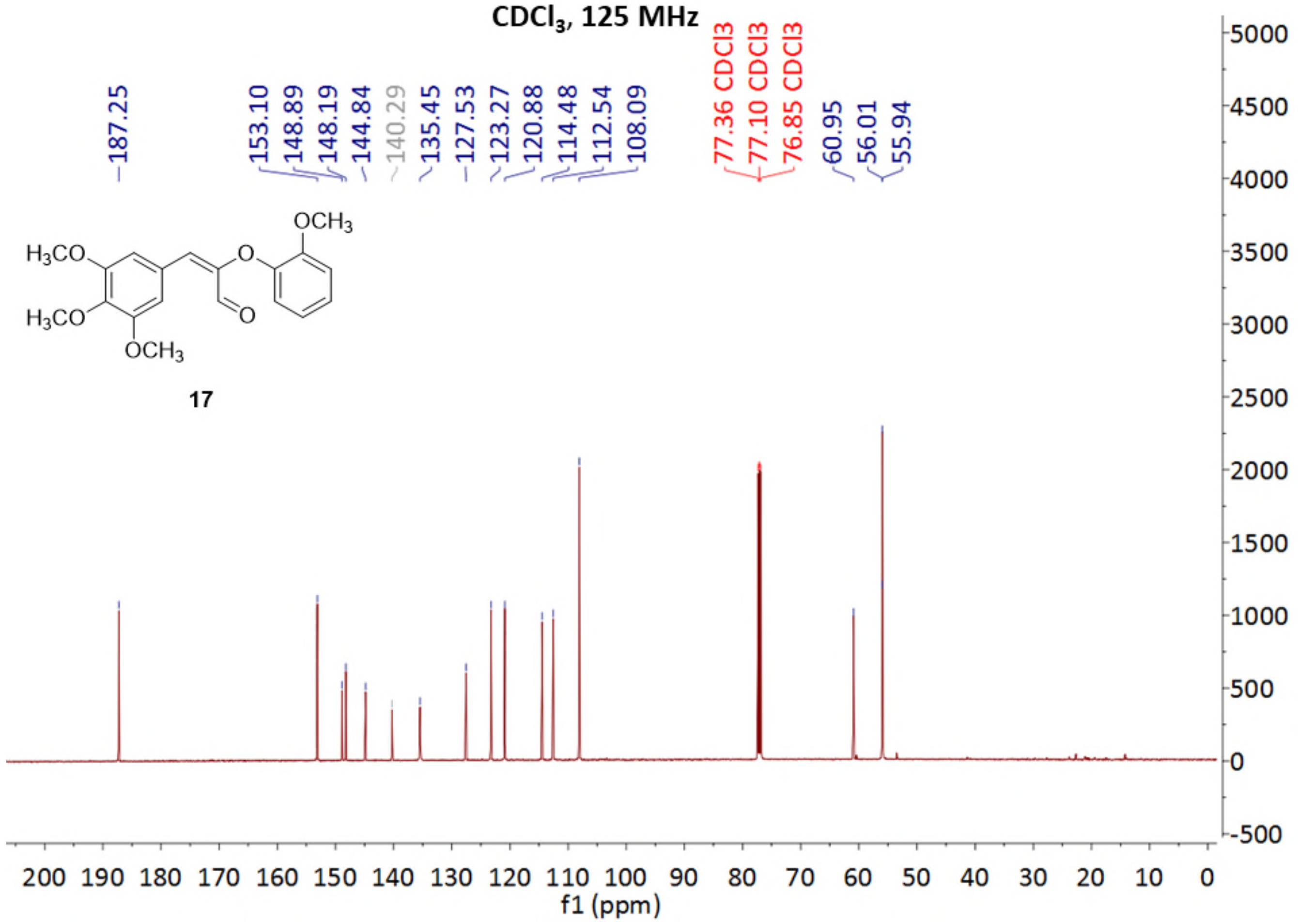
CDCl3, 125 MHz
187.25
153.10
148.89
148.19
144.84
140.29
135.45
127.53
123.27
120.88
114.48
112.54
108.09
77.36 CDCl3
77.10 CDCl3
76.85 CDCl3
60.95
56.01
55.94
OCH3
H3CO
H3CO
OCH3
O
O
17
f1 (ppm)

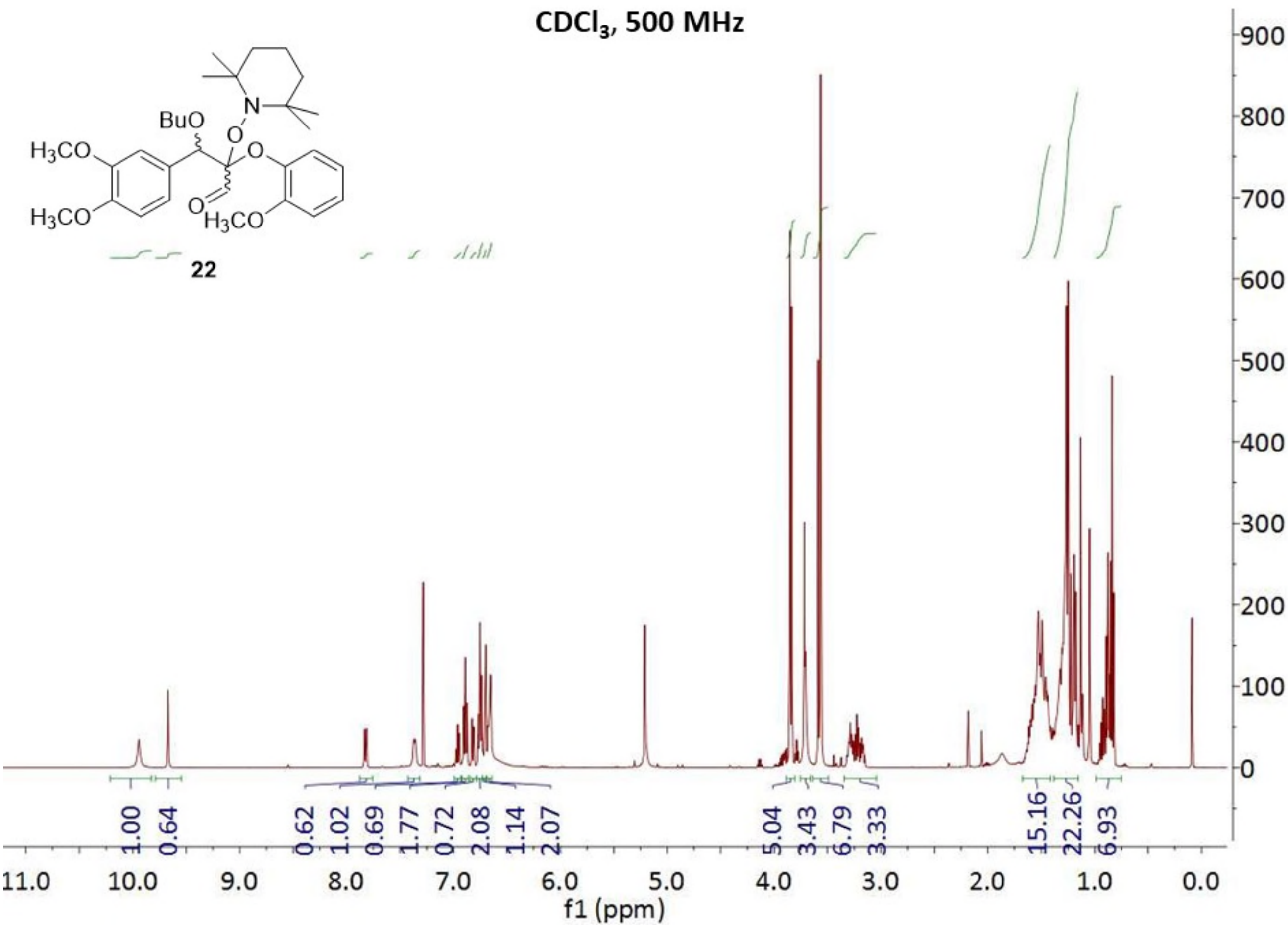
CDCl3, 500 MHz
BuO
H3CO
H3CO
O
H3CO
N
22
1.00
0.64
0.62
1.02
0.69
1.77
0.72
2.08
1.14
2.07
5.04
3.43
6.79
3.33
15.16
22.26
6.93
f1 (ppm)

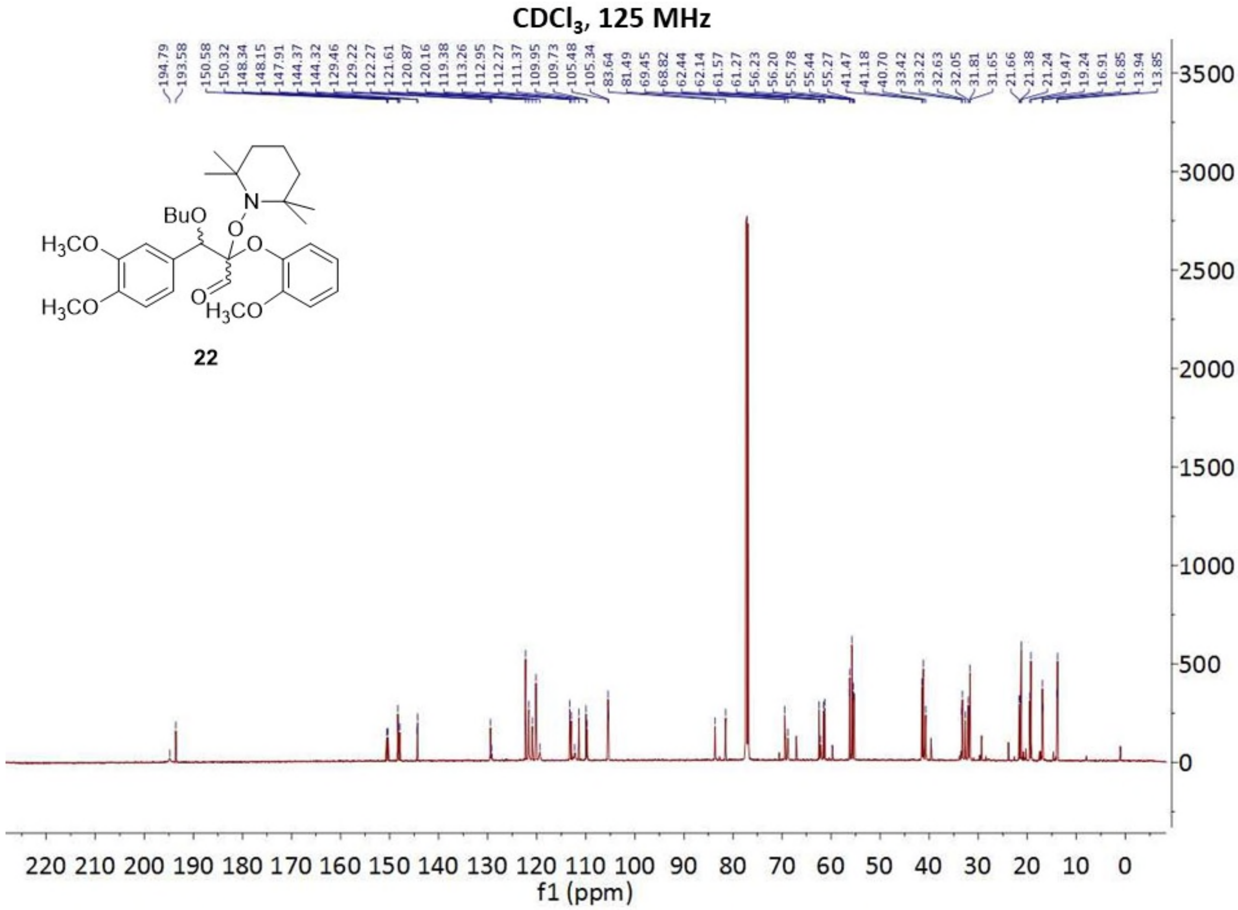
CDCl3, 125 MHz
194.79
193.58
150.58
150.32
148.34
148.15
147.91
144.37
144.32
129.46
129.22
122.27
121.61
120.87
120.16
119.38
113.26
112.95
112.27
111.37
109.95
109.73
105.48
105.34
83.64
81.49
69.45
68.82
62.44
62.14
61.57
61.27
56.23
56.20
55.78
55.44
55.27
41.47
41.18
40.70
33.42
33.22
32.63
32.05
31.81
31.65
21.66
21.38
21.24
19.47
19.24
16.91
16.85
13.94
13.85
BuO
H3CO
H3CO
O
H3CO
N
22
f1 (ppm)

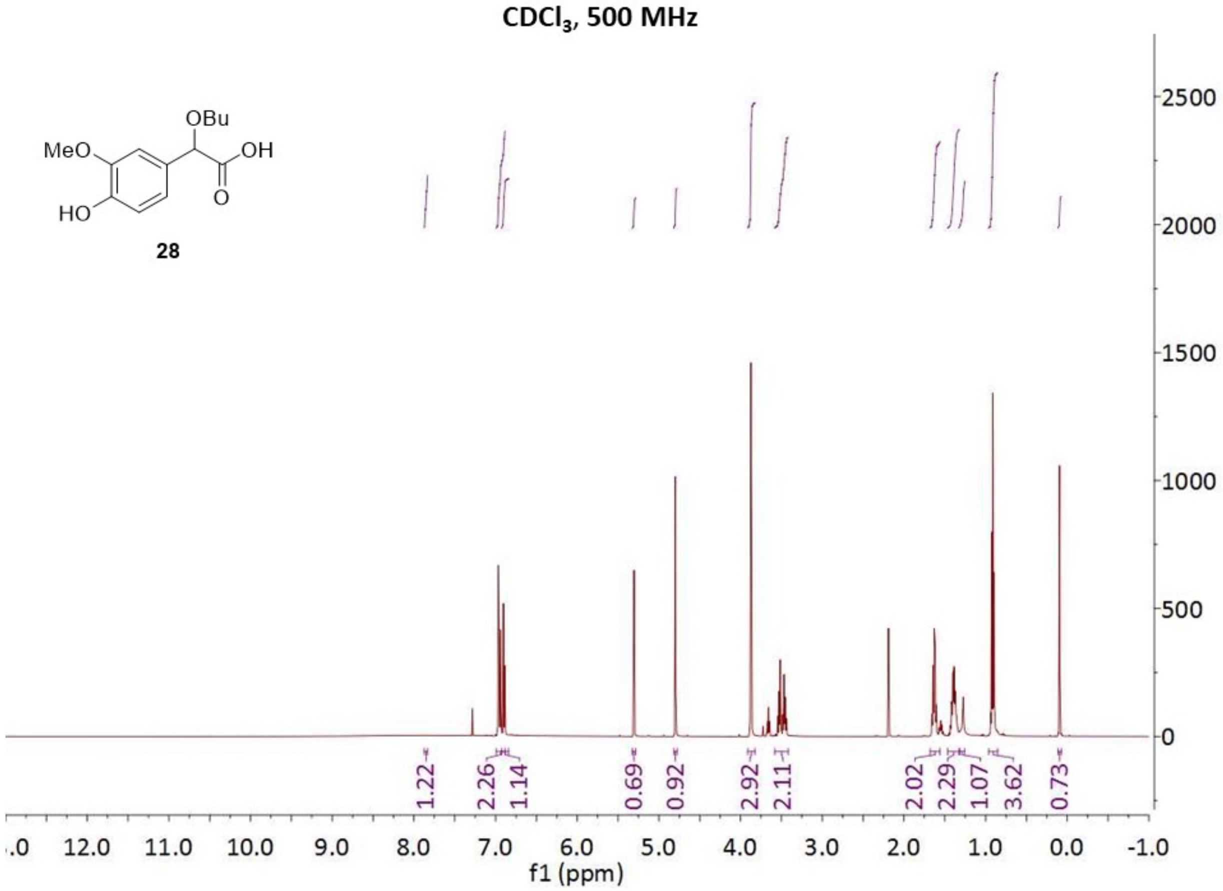
CDCl3, 500 MHz
OBu
MeO
OH
O
HO
28
1.22
2.26
1.14
0.69
0.92
2.92
2.11
2.02
2.29
1.07
3.62
0.73
f1 (ppm)

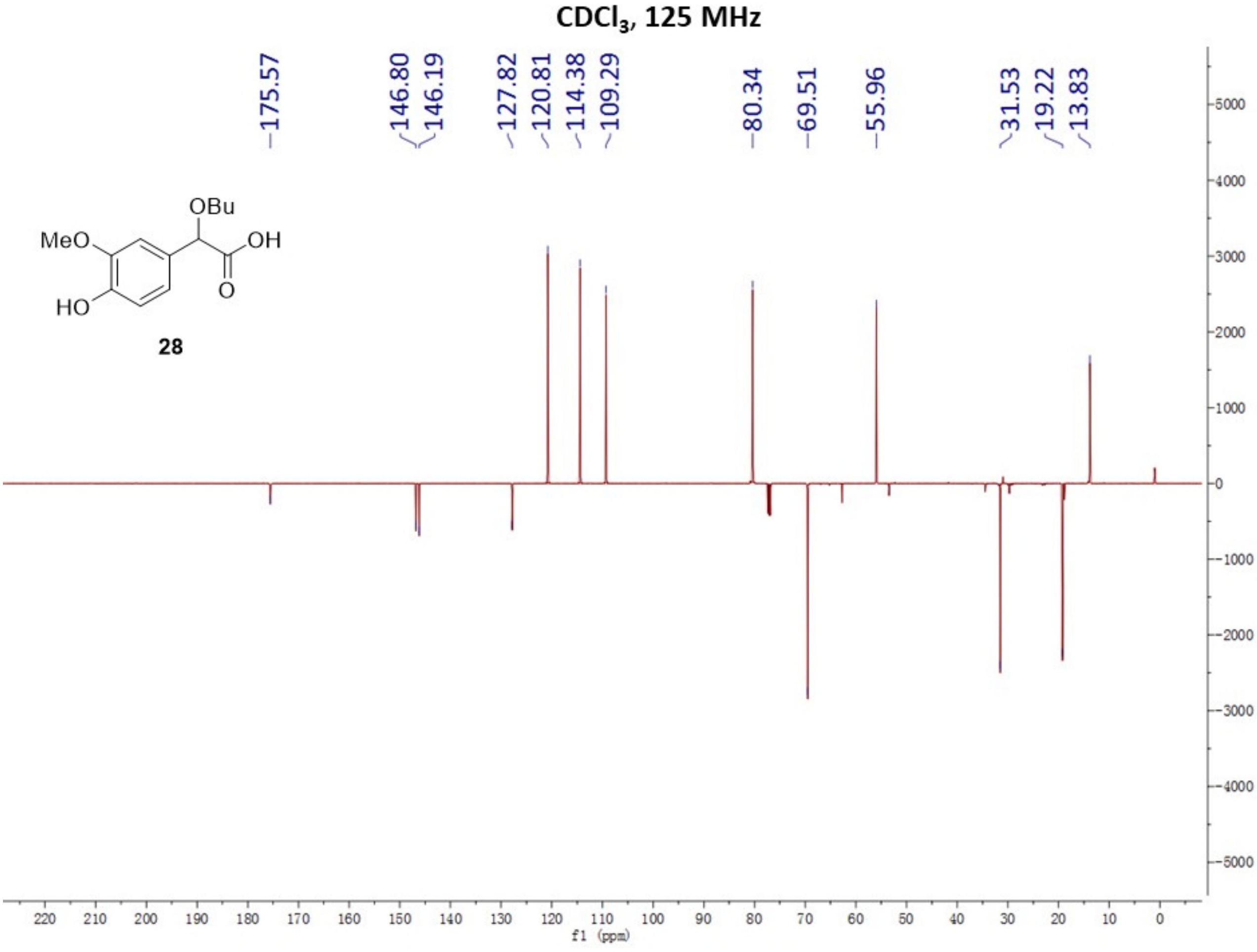
CDCl3, 125 MHz
175.57
146.80
146.19
127.82
120.81
114.38
109.29
80.34
69.51
55.96
31.53
19.22
13.83
OBu
MeO
OH
O
HO
28
f1 (ppm)

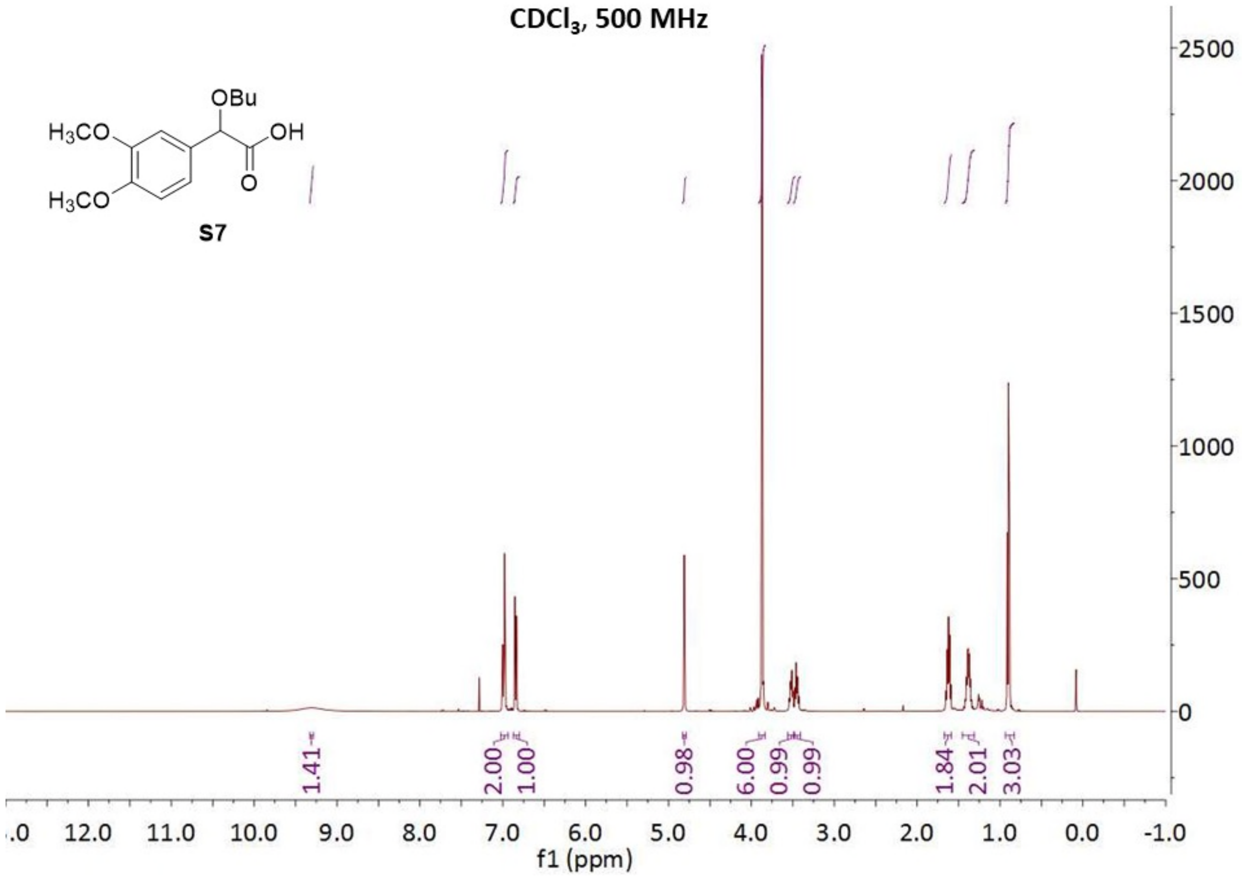
CDCl3, 500 MHz
OBu
H3CO
OH
H3CO
O
S7
1.41
2.00
1.00
0.98
6.00
0.99
0.99
1.84
2.01
3.03
f1 (ppm)

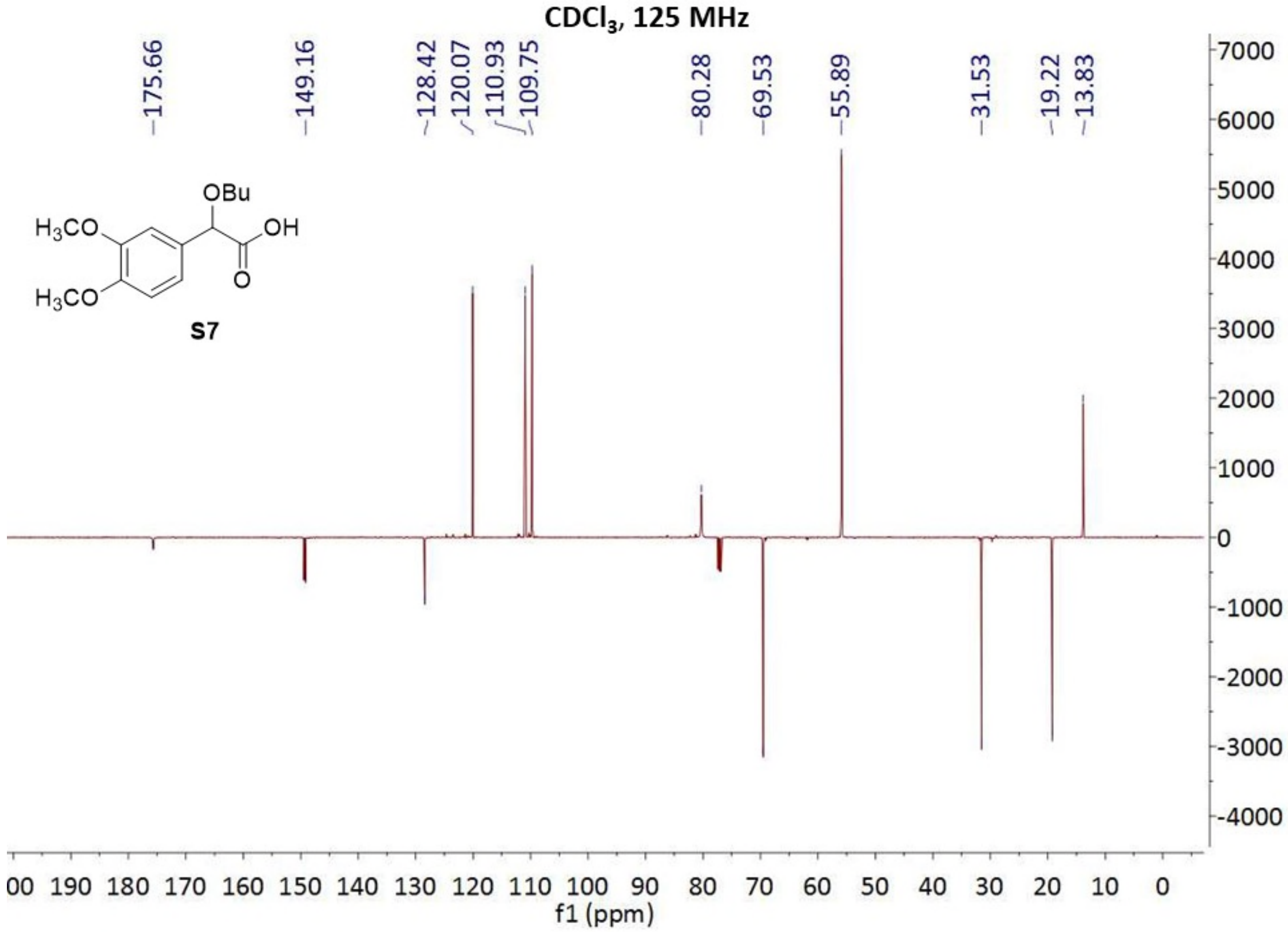
CDCl3, 125 MHz
175.66
149.16
128.42
120.07
110.93
109.75
80.28
69.53
55.89
31.53
19.22
13.83
OBu
H3CO
OH
H3CO
O
S7
f1 (ppm)

$CDCl_3$, 400 MHz

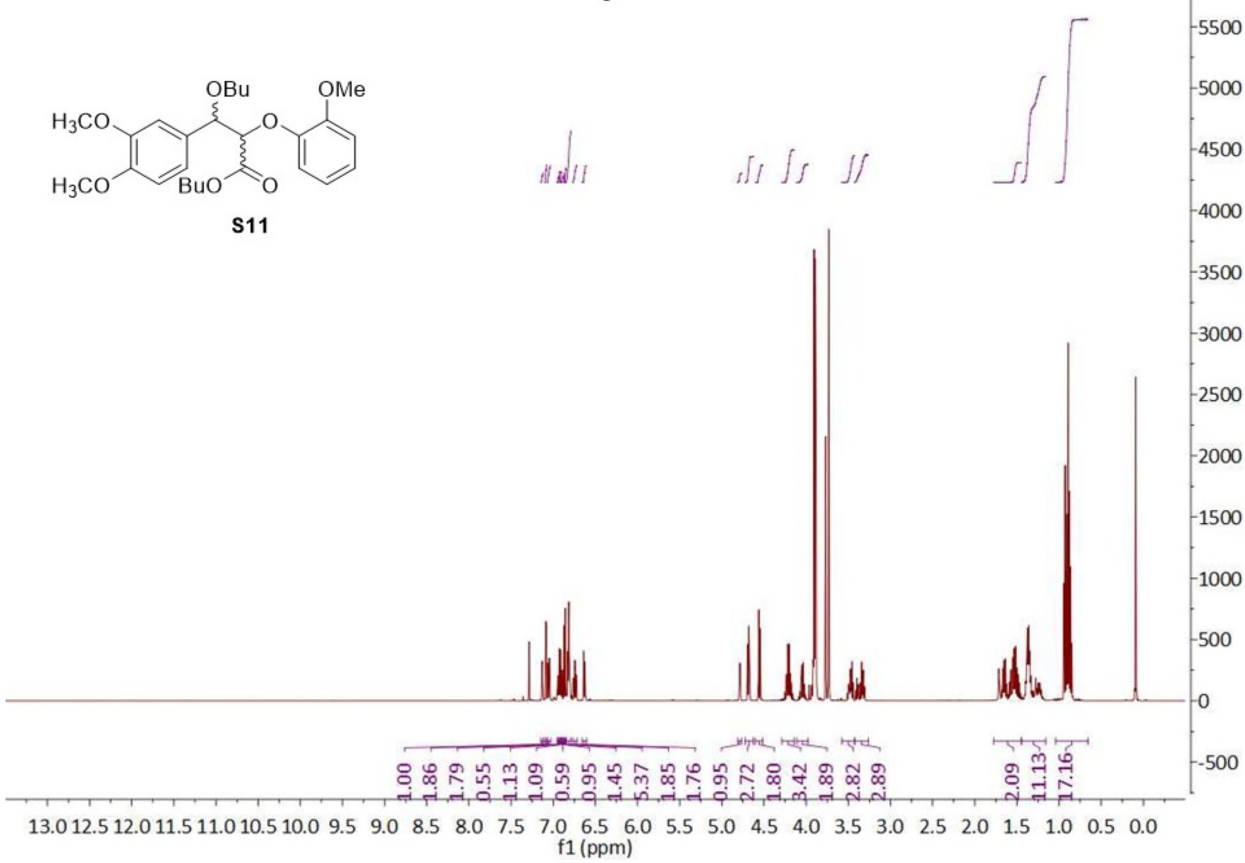

$CDCl_3$, 100 MHz

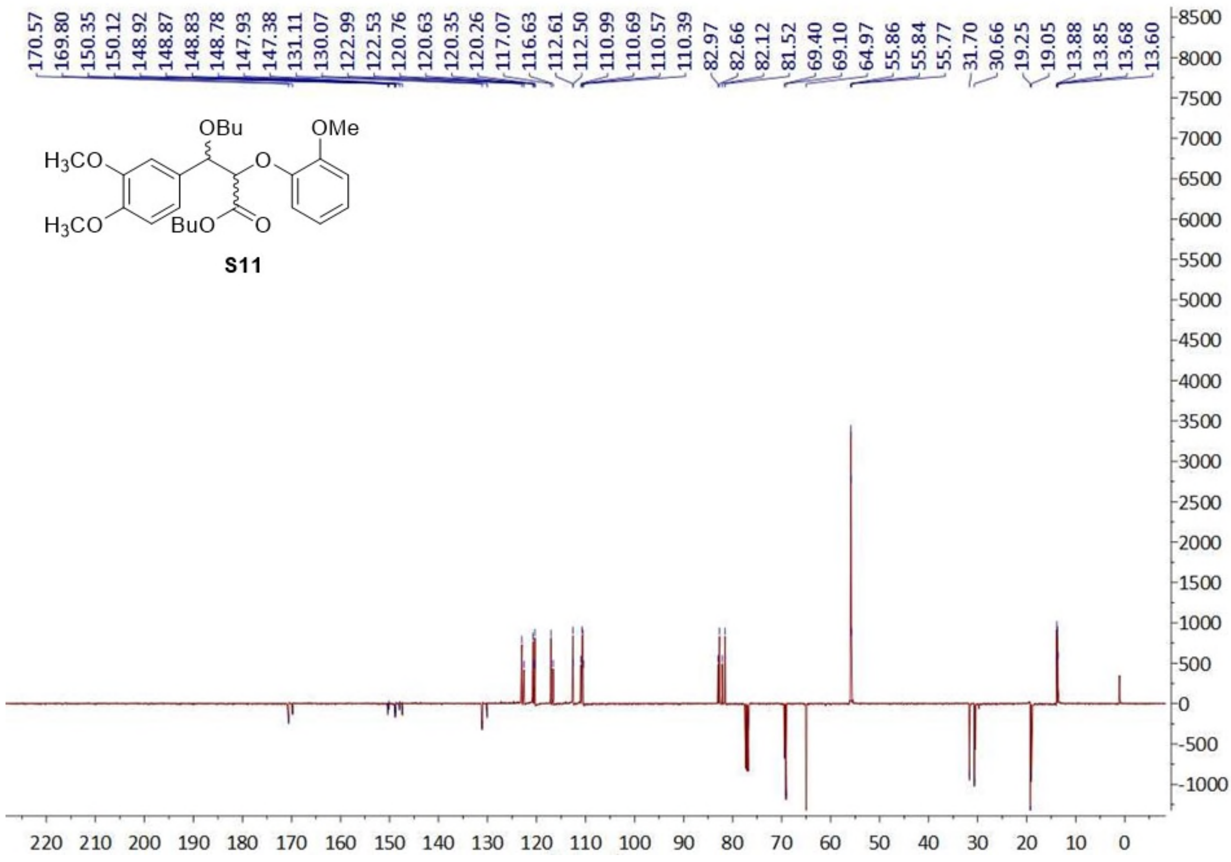

13. Reference

S1. G. Xiao, C. S. Lancefield and N. J. Westwood, *ChemCatChem*, 2019, **11**, 3182-3186.

S2. H. E. P. Salonen, C. P. Mecke, M. I. Karjomaa, P. M. Joensuu and A. M. Koskinen, *ChemistrySelect*, 2018, **3**, 12446-12454.

S3. B. Sedai, C. Díaz-Urrutia, R. T. Baker, R. Wu, L. P. Silks and S. K. Hanson, *ACS Catal*, 2011, **1**, 794-804.

S4. A. Hajipour, S. Mallakpour and S. Khoee, *Synth. Commun*, 2002, **32**, 9-15.

S5. J. Cheng, M. Zhu, C. Wang, J. Li, X. Jiang, Y. Wei, W. Tang, D. Xue and J. Xiao, *Chem. Sci*, 2016, **7**, 4428-4434.

S6. K. Lee and R. E. Maleczka, *Org. Lett*, 2006, **8**, 1887-1888.

S7. N. Kornblum and H. E. DeLaMare, *J. Am. Chem. Soc*, 1951, **73**, 880-881.

S8. J. D. Nguyen, B. S. Matsuura and C. R. Stephenson, *J. Am. Chem. Soc*, 2014, **136**, 1218-1221.

S9. E. O. Pentsak, D. B. Eremin, E. G. Gordeev and V. P. Ananikov, *ACS Catal*, 2019, **9**, 3070-3081.

S10. A. C. Lindsay, S. Kudo and J. Sperry, *Org. Biomol. Chem*, 2019, **17**, 7408-7415.

S11. J. R. Montgomery, C. S. Lancefield, D. M. Miles-Barrett, K. Ackermann, B. E. Bode, N. J. Westwood and T. Lebl, *ACS Omega*, 2017, **2**, 8466-8474.

S12. C. S. Lancefield, O. S. Ojo, F. Tran and N. J. Westwood, *Angew. Chemie - Int. Ed*, 2015, **54**, 258-262.

S13. C. S. Lancefield and N. J. Westwood, *Green Chem*, 2015, **17**, 4980-4990.

S14. K. Soai and A. Ookawa, *J. Org. Chem*, 1986, **51**, 4000-4005.

S15. I. Panovic, C. S. Lancefield, D. Phillips, M. J. Gronnow and N. J. Westwood, *ChemSusChem*, 2019, **12**, 542-548.

S16. J.-A. Jiang, C. Chen, Y. Guo, D.-H. Liao, X.-D. Pan and Y.-F. Ji, *Green Chem*, 2014, **16**, 2807-2814.
